# A Chemically Fuelled Molecular Automaton Displaying Programmed Migration of Zn^2+^ Between Alternative Binding Sites

**DOI:** 10.1002/chem.202202247

**Published:** 2022-08-17

**Authors:** Matthew M. Wootten, Sofja Tshepelevitsh, Ivo Leito, Jonathan Clayden

**Affiliations:** ^1^ School of Chemistry University of Bristol Cantock's Close Bristol BS8 1TS UK; ^2^ Institute of Chemistry University of Tartu Ravila 14a Tartu 50411 Estonia

**Keywords:** chemical fuel, circular dichroism, host-guest systems, metal complexes, molecular automata

## Abstract

A molecular system comprising a cationic zinc complex and an amino acid‐derived ambident ligand having phosphate and carboxylate binding sites undergoes a series of rearrangements in which the metal cation migrates autonomously from one site to another. The location of the metal is identified by the circular dichroism spectrum of a ligated bis(2‐quinolylmethyl)‐(2‐pyridylmethyl)amine (BQPA) chromophore, which takes a characteristic shape at each binding site. Migration is fuelled by the decomposition of trichloroacetic acid to CO_2_ and CHCl_3_, which progressively neutralises the acidity of the system as a function of time, revealing in sequence binding sites of increasing basicity. The migration rate responds to control by variation of the temperature, water content and triethylamine concentration, while an excess of fuel controls the duration of an induction period before the migration event.

## Graphical abstract

**Programmed autonomous** migration of cations between alternative binding sites of a peptidomimetic ligand is driven by a fuelled change in acidity of the system. The rate of migration, and delays or resets in the execution of the program, may be controlled by the operating conditions of the molecular automaton.

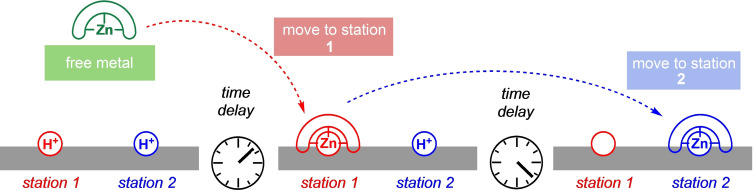

## Introduction

Life is sustained by a collection of simultaneously operating autonomous networks of molecular interactions, using chemical energy to drive cyclic, out‐of‐equilibrium reactions.^[1]^ Significant recent advances have been made in the development of autonomous artificial molecular machines,[^2^, ^3^, ^4^, ^5^, ^6^, ^7^] self‐assembling systems[^8^, ^9^, ^10^, ^11^] and membrane‐bound signal transducers[^12^, ^13^, ^14^] with the aim of developing synthetic systems with primitive lifelike behaviour.[^15^, ^16^, ^17^] In this paper we report a ‘molecular automaton’[^18^, ^19^, ^20^] in which movement of metal cations between the alternative ‘stations’ of a single ambident ligand is fuelled by the decomposition of trichloroacetic acid (TCAH).

**Figure 1:**
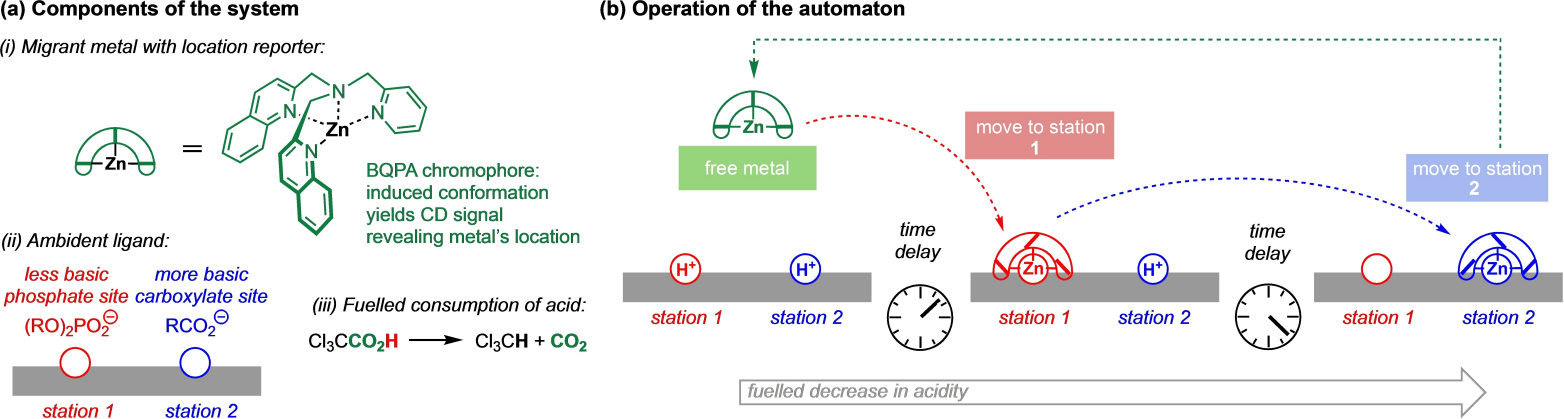
(a) Components of the system: (i) A Zn^2+^ cation tightly bound to a BQPA ligand whose induced CD spectrum reports the local chiral environment of the Zn^2+^; (ii) an ambident ligand with a phosphate and a carboxylate binding site, each in a local chiral environment; (iii) trichloroacetic acid as a fuel whose decomposition removes acidic species from the system. (b) Operation of the system. In the presence of acid, the [Zn(BQPA)]^2+^ complex remains in solution. As the acidity decreases according to a time regime determined by the consumption of the fuel, the metal moves first to the less basic (station 1) and then to the more basic site (station 2).

We have previously shown that the selective binding of a Zn^2+^ cation to a set of monoprotic ligands of differing p*K*_a_ may be controlled by addition of acid or base, modulating the protonation states of the various ligands in such a way that the Zn^2+^ cation associates chemoselectively with the most basic of the available free anions.^[21]^ Exchange between two equilibrium states, as one ligand was displaced by another, was detected by changes in the circular dichroism (CD) spectrum of a bis(2‐quinolylmethyl)‐(2‐pyridylmethyl)amine chromophore (BQPA, Figure 1a(i))[^22^, ^23^] also bound at the Zn^2+^ centre. We now show that regioselective metal‐ligand coordination may be controlled in an out‐of‐equilibrium system in which pH evolves autonomously as a result of a chemically fuelled deacidification process [Figure 1a(iii)]. When a single ligand with multiple binding sites [Figure 1a(ii)] is used instead of a set of monodentate ligands, we find that the Zn^2+^ complex migrates autonomously from one binding site to another as the deacidification unmasks in sequence one site after another in a stepwise manner (Figure 1b). The location of the complex is revealed by the BQPA chromophore whose CD signal is distinguishably different at the alternative binding sites of the ligand.

## Results and Discussion

The metabolite phosphothreonine **1** offers an archetype of the type of polyprotic, ambident ligand that is required,^[24]^ combining potential binding sites of differing acidity with chirality capable of inducing a detectable CD signal. With **1** as a model, we set about constructing suitable synthetic mimics that could later be combined into a polyprotic target ligand. The ability of the phosphate binding site to induce a CD signal was optimised by first varying the steric demand of the phosphate *O*‐substituent R^1^ in L ‐threonine analogues **2 a**–**c**, then varying the *N*‐acyl substituent R^2^ (Table 1), whilst ensuring that all groups tested were acid‐stable. Ligands **2 d**–**f** were derivatised as *n*‐butyl amides to mimic the local environment in a peptide ligand. The best performing analogue **2 f** features bulky *tert*‐butyl groups at the phosphate oxygen and α‐nitrogen atoms, which were both required for strong CD signal induction.

**Table 1:** Structure optimisation for phosphothreonine derivatives **2 a‐f**.

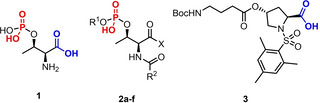				
Ligand	X	R^1^	R^2^	[θ]_239.5_/deg dm^2^ mol^−1[a]^
**2 a**	OH	Ph	CH_2_CH_2_Ph	+5,700
**2 b**	OH	CH_2_CH_2_Ph	CH_2_CH_2_Ph	+10,700
**2 c**	OH	CH_2_ *t*Bu	CH_2_CH_2_Ph	+14,100
**2 d**	NH*n*Bu	CH_2_ *t*Bu	CH_2_CH_2_Ph	+11,600
**2 e**	NH*n*Bu	CH_2_ *t*Bu	CH_3_	+12,500
**2 f**	NH*n*Bu	CH_2_ *t*Bu	*t*Bu	+15,200

[a] Molar ellipticity maximum at 239.5 nm induced upon addition of 1.0 equiv. NEt_3_ to Zn(BQPA) ⋅ 2ClO_4_ (*c*_o_=0.25 mM, MeCN) and **2 a**–**f** (1 equiv.). See Supporting Information for complete NEt_3_ titration data.

Carboxylic acid **3**, derived from L‐hydroxyproline (Hyp), displays (i) a mesitylsulfonyl group as a sterically demanding, acid‐stable nitrogen protecting group, (ii) a Boc‐γ‐aminobutyryl (GABA) side chain as a spacer unit and handle for further derivatisation, and (iii) a *trans* relationship between the carboxylate and γ‐oxygen to minimise steric interaction between the binding site and the side chain.

**Figure 2:**
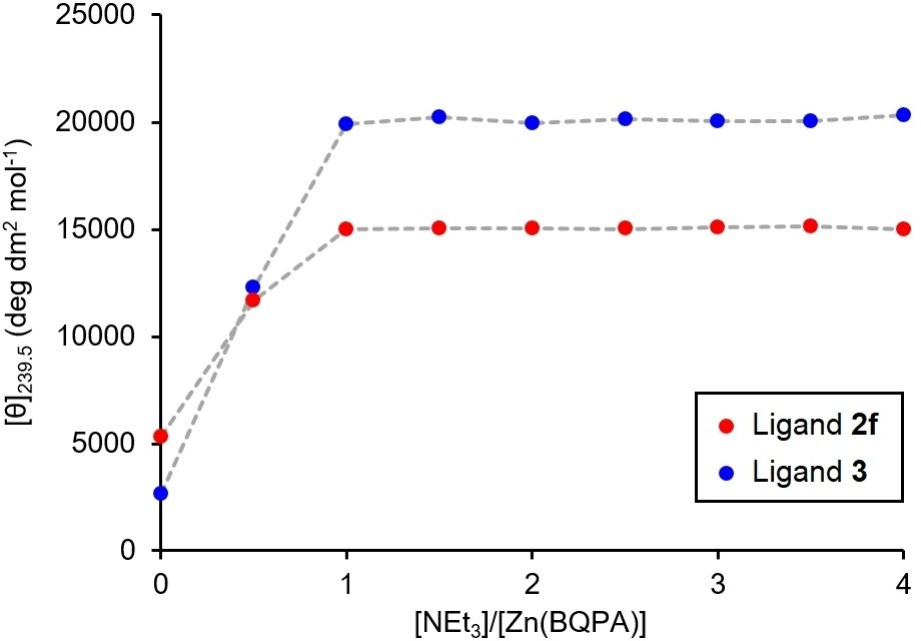
Molar ellipticities at 239.5 nm of Zn(BQPA) ⋅ 2ClO_4_ (c_0_=0.25 mM, MeCN) and **2 f** or **3** with addition of NEt_3_ (0.0→4.0 equiv.) in 0.5 equiv. increments.

Equimolar 0.25 mM solutions of Zn(BQPA) ⋅ 2ClO_4_ and either **2 f** or **3** in MeCN were titrated with triethylamine (Figure 2). Upon deprotonation, both **2 f** and **3** induced strong, positive CD signals at 239.5 nm from the BQPA ligand. Phosphate **2 f** reached a maximum of +15,200 deg dm^2^ mol^−1^, while carboxylate **3** reached a maximum of +20,000 deg dm^2^ mol^−1^ at 1 equivalent of base. Further addition of base led to no change in signal for either ligand, indicating complete deprotonation with 1 equiv. NEt_3_ in both cases.

The two optimised ligand structures **2 f** and **3** were merged to give the Thr‐GABA‐Hyp derived peptide **4**, which was synthesised from L‐Hyp in nine steps with an overall yield of 25 % (see Supporting Information for synthetic details). In order to track the movement of the Zn^2+^ cation between the binding sites by CD, the two binding sites must induce Cotton effects of opposite signs in the BQPA location reporter. We therefore used D‐Thr to invert the sign of the CD signal generated by binding at the phosphate station. The GABA spacer unit gives a total of 16 bonds between binding sites, reducing the risk that both sites will bind simultaneously to the Zn^2+^.

**Figure 3:**
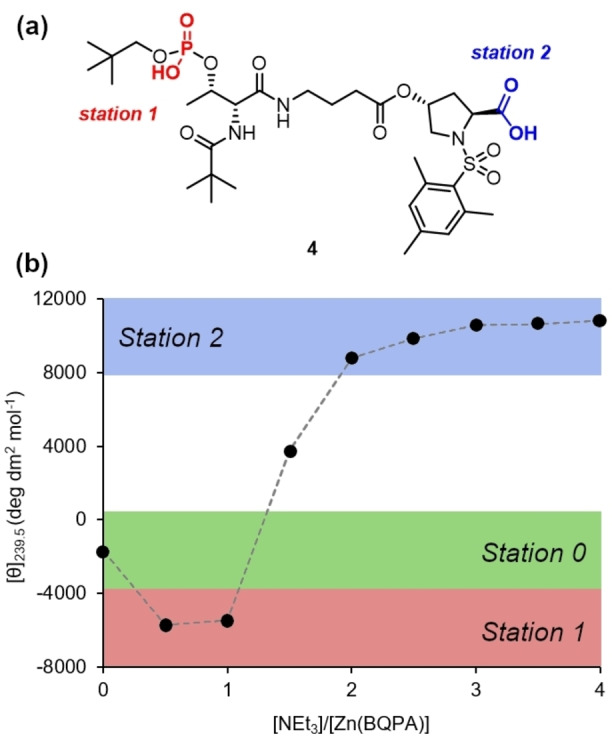
(a) Structure of peptide ligand **4**; (b) Molar ellipticity at 239.5 nm of Zn(BQPA) ⋅ 2ClO_4_ (c_0_=0.25 mM, MeCN) and **4** (1 equiv.) with addition of NEt_3_ (0.0→4.0 equiv.) in 0.5 equiv. increments. Coloured bands indicate the molar ellipticities characteristic of each station of the ligand.

Titration of an equimolar solution of Zn(BQPA) ⋅ 2ClO_4_ (0.25 mM, MeCN) and **4** with NEt_3_ showed the behaviour we were seeking. Initially, the neutral ligand does not bind appreciably to zinc, and only a weak CD response is measured (Figure 3b). As the first equivalent of base is added, the more acidic phosphate site (‘station 1’ of Figure 1) is revealed, and a negative CD signal develops at 239.5 nm as the [Zn(BQPA)]^2+^ complex migrates to that site. A second equivalent of base further deprotonates the ligand to form a dianion, revealing a carboxylate site (‘station 2’ of Figure 1) capable of binding Zn^2+^ more tightly than the phosphate does. As a result, Zn^2+^ migrates (either intra‐ or intermolecularly) from the phosphate to the carboxylate, and the BQPA reports the change in environment in the form of a switch to a positive CD signal at 239.5 nm, a response that is complete on addition of 3 equiv. of base.

Evidence that this binding selectivity arises from the difference in p*K*_a_ between the two stations was provided by calculations using the COSMO‐RS[^25^, ^26^, ^27^] method (see Supporting Information). Using this method, we estimate p*K*_a_ values in pure acetonitrile of 15.0±2.0 for station 1 and 21.8±1.3 for station 2. The difference between the p*K*_a_ values of the stations is most likely over 3 p*K*_a_ units, enough to provide almost complete pH‐dependent binding selectivity. We furthermore estimate, using weighted average positive sigma (WAPS) values as computational descriptors of charge delocalisation,^[28]^ that addition of 1–2 % water will not substantially alter this p*K*_a_ difference.

The binding constant of the carboxylate station was estimated to be *K*=1.19±0.15×10^6^ M^−1^ at 25 °C by titration of [Zn(BQPA)]^2+^ with an equimolar mixture of **3** and the phosphazene base *t*BuP_1_(NMe_2_)_3_ (see Supporting Information). **3** displayed 1 : 1 host:guest binding behaviour. The binding isotherm of **2 f** was more complex (see Supporting Information).

Autonomous migration of Zn^2+^ must be powered by a fuel, for which we chose TCAH, which decomposes under basic conditions by decarboxylation of its conjugate base TCA^−^. The kinetics of this reaction have been extensively studied in water, organic solvents, and solvent mixtures.[^29^, ^30^, ^31^, ^32^] Since the decomposition of TCAH produces non‐acidic products CO_2_ and CHCl_3_, it is accompanied by an overall loss of acidity. The consequent time‐dependent increase in pH has been exploited to fuel molecular motion in supramolecular assemblies including rotaxane shuttling,^[33]^ rotary motors,^[34]^ catalyst activation,^[35]^ crystallisation^[36]^ and gelation.^[37]^ α‐Cyano carboxylic acids have similarly been used as a chemical fuel to control the pH‐dependent conformations of catenanes^[38]^ and calixarenes,[^39^, ^40^] and also to temporarily release Zn^2+^ from an aza‐crown ether host in a self‐sorting multicomponent system.^[41]^

**Figure 4:**
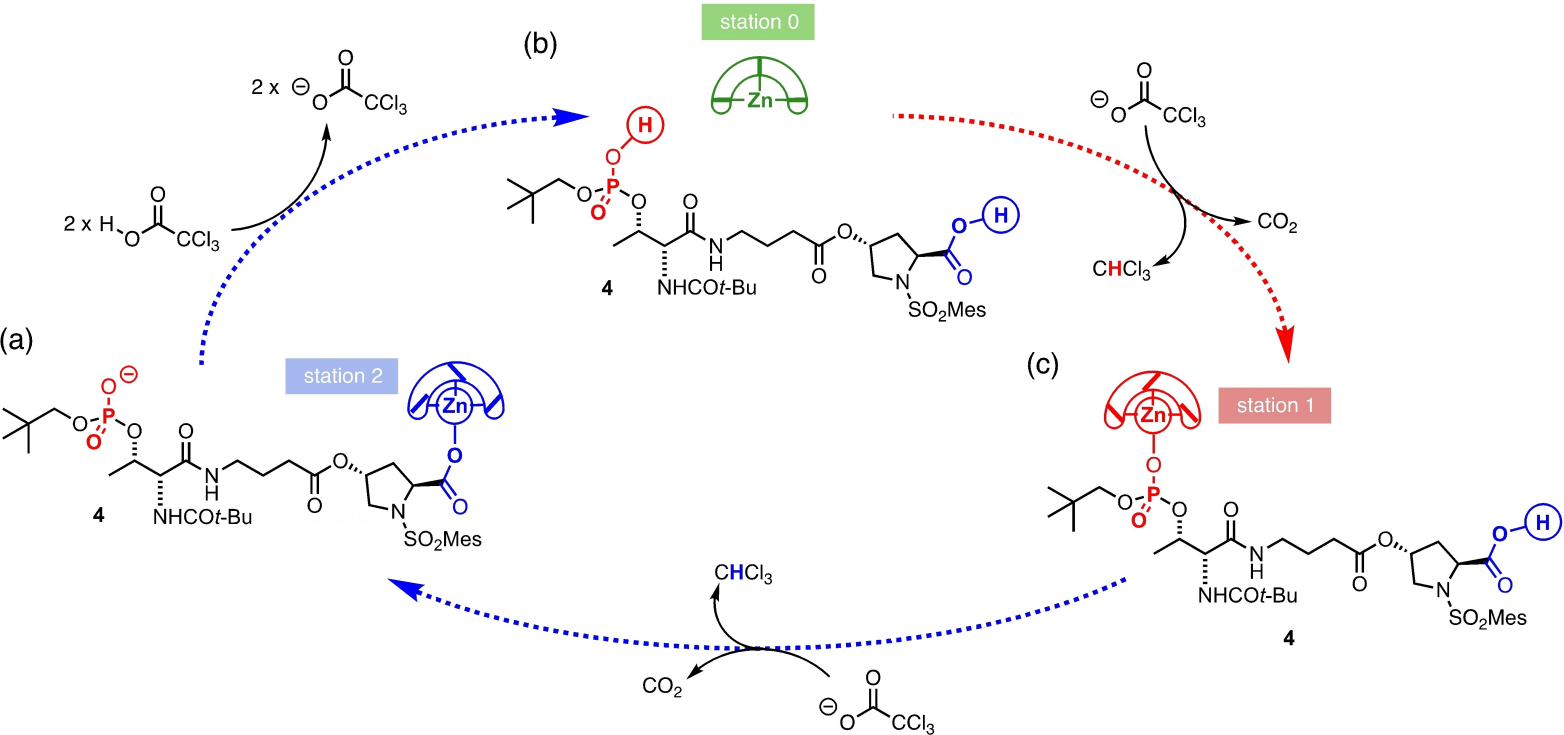
Schematic representation of TCAH‐fuelled Zn migration around ligand **4** (triethylammonium cations omitted for clarity): (a) Carboxylate‐bound resting state (Station 2); (b) ‘Off’ state (Station 0) following protonation of both binding sites by priming with 2 equiv. TCAH; (c) Transient phosphate‐bound state (Station 1) following deprotonation by a chloroform anion. Further deprotonation returns the system to the resting state (a).

We applied the TCAH‐fuelled pH change to the multi‐site binding system using ligand **4**. The operation of the autonomous system is illustrated in Figure 4. The resting state is one in which both binding sites of **4** are deprotonated (Figure 4a), and the Zn^2+^ occupies the more basic carboxylate station (station 2). Addition of two equivalents of TCAH (p*K*_a_(MeCN)=10.93)^[42]^ protonates both sites, priming the automaton by releasing [Zn(BQPA)]^2+^ into solution (station 0) (b). The deprotonated fuel decomposes slowly and continuously, transferring protons from the binding sites to chloroform irreversibly. Decomposition of the first equivalent of TCA^−^ deprotonates the more acidic phosphate site (station 1), which captures from solution the [Zn(BQPA)]^2+^ ions (c). Further decomposition of the remaining TCA^−^ deprotonates the carboxylic acid, causing the zinc to migrate to the carboxylate station and returning the system to the resting state (station 2) (a). In this way, a dynamic system emerges in which the binding selectivity of the metal complex is autonomously shifted from one site, or station, of the ligand to another in a manner that is programmed by the time‐course of the deacidification of the solution.

**Figure 5:**
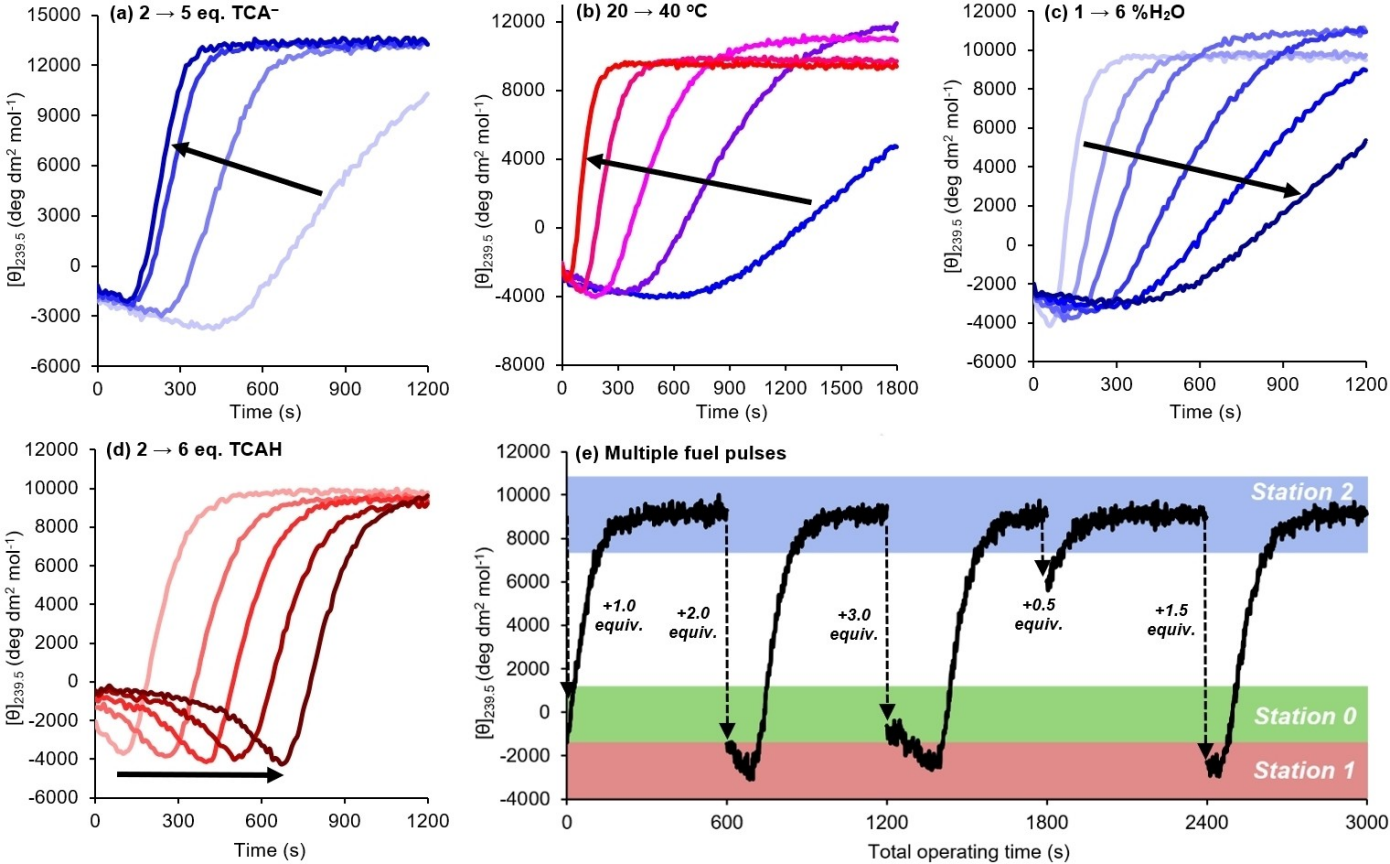
Time‐course molar ellipticity measurement at 239.5 nm of Zn(BQPA) ⋅ 2ClO_4_ (c_o_=0.25 mM) and **4** (1 equiv.) with (a) NEt_3_ (2–5 equiv.) and TCAH (2–5 equiv) in 2 % H_2_O/MeCN at 25 °C; (b) NEt_3_ (2 equiv) and TCAH (2 equiv) in 2 % H_2_O/MeCN at 20–40 °C; (c) NEt_3_ (2 equiv) and TCAH (2 equiv) in 1–6 % H_2_O/MeCN at 35 °C; (d) NEt_3_ (2 equiv) and TCAH (2–6 equiv) in 2 % H_2_O/MeCN at 35 °C; and (e) Zn(BQPA) ⋅ 2ClO_4_ (c_o_=0.025 mM) **4** (1 equiv.) and NEt_3_ (2 equiv.) in 2 % H_2_O/MeCN at 35 °C, with pulses of 1.0, 2.0, 3.0, 0.5 then 1.5 equiv. TCAH.

The molecular automaton was constructed by mixing Zn(BQPA) ⋅ 2ClO_4_ (0.25 mM), **4** (1 equiv), NEt_3_ (3 equiv.) in MeCN containing 2 % H_2_O,^[43]^ and primed by adding TCAH (3 equiv.) which initiated operation of the automaton starting at station 0. Progress of the system through the programmed series of steps was monitored by molar ellipticity at 239.5 nm over 20 min (Figure 5a). As we had hoped, the BQPA ‘location reporter’ signal decreased over the first few minutes, reaching a negative maximum of −3,000 deg dm^2^ mol^−1^ at around 4 minutes, as the zinc migrated to the phosphate station 1. The location reporter signal then swung to positive and increased in magnitude over the next 16 minutes, corresponding to carboxylate deprotonation and migration of Zn^2+^ to the carboxylate station 2.

Increased amounts of both NEt_3_ and TCAH up to 5 equivalents were accompanied by an increase in the rate of operation; the rapid growth of the phosphate signal showed that the zinc arrived earlier at the phosphate station 1, with the journey time decreasing from 420 seconds (at 2 equiv.) to 110 seconds (at 5 equiv.). Similarly, it migrated faster to the carboxylate station 2, as the carboxylate signal plateaued earlier following a steeper gradient.^[44]^

Higher temperatures likewise led to faster TCA^−^ decomposition and faster Zn^2+^ migration. Samples containing Zn(BQPA) ⋅ 2ClO_4_ (0.25 mM, MeCN with 2 % H_2_O), **4** (1 equiv.), NEt_3_ (2 equiv.) and TCAH (2 equiv.) were monitored over 30 minutes at temperatures between 20–40 °C (Figure 5b). Steeper gradients were observed as the temperature was increased; the time taken for Zn^2+^ to arrive at the phosphate station approximately halved with every 5 °C increase. The time taken for the system to return to the carboxylate station also decreased at higher temperatures; the output signal plateaued after 6 minutes at 40 °C, while no plateau was observed within 30 minutes below 25 °C.^[45]^

Since the TCAH stock solution in MeCN required a water content of 20 vol % to prevent spontaneous decomposition,^[43]^ it followed that the rate of Zn^2+^ migration could also be controlled by varying the water content of the sample. Samples containing Zn(BQPA) ⋅ 2ClO_4_ (0.25 mM), **4** (1 equiv.), NEt_3_ (2 equiv.) and TCAH (2 equiv.) were prepared in MeCN containing 1–6 vol % H_2_O and monitored over 20 minutes at 35 °C (Figure 5c). The rate of migration decreased as the water content increased, as water stabilises the TCA^−^ anion via hydrogen bonding.

With excess TCAH the site‐hopping event may be delayed by an induction period, as migration to the phosphate site can only begin once excess TCAH is depleted.^[39]^ Samples containing Zn(BQPA) ⋅ 2ClO_4_ (0.25 mM, MeCN with 2 % H_2_O), **4** (1 equiv.), NEt_3_ (2 equiv.) and TCAH (2–6 equiv.) were monitored over 20 minutes at 35 °C (Figure 5d). The excess TCAH leads to a travel delay which increases linearly by an average of 139 seconds for each additional equiv. of TCAH (0.25 mM), giving a zero‐order decomposition rate constant of *k*=1.8×10^−6^ mol dm^−3^ s^−1^ during the induction period. TCA^−^ concentration remains constant until TCAH is fully consumed, but once excess TCAH is depleted, the Zn^2+^ starts its journey first to the phosphate and then the carboxylate station, but at the same rate, regardless of the initial TCAH concentration.

Finally, multiple additions of fuel were used enforce restarts of the migration program. A sample of Zn(BQPA) ⋅ 2ClO_4_ (*c*_o_=0.025 mM,^[46]^ MeCN with 2 vol % H_2_O), **4** (1 equiv.) and NEt_3_ (2 equiv.) was consecutively treated with 1.0, 2.0, 3.0, 0.5 and 1.5 equiv. of TCAH, with a 10 minute observation period between fuel pulses (Figure 5e). By varying the amount of TCAH added, the migration process could be reprogrammed to start at any point between stations 0 and 2. After each fuel pulse, the system returned to the same resting state output of +9,100 deg dm^2^ mol^−1^ within 10 minutes. A further experiment in which the automaton was repeatedly restarted with ten successive additions of 2.0 equiv. TCAH (see Supporting Information) indicated that the operating rate decreased marginally with each cycle, owing to accumulation of the water required to stabilize the fuel.

## Conclusion

We have demonstrated migration of a cationic metal complex [Zn(BQPA)]^2+^ between different sites on an ambidentate peptide ligand **4**, in which the location of the metal is controlled by the pH of the medium. Coupling the migration of the metal between sites with TCA^−^ decomposition creates a chemically fuelled molecular automaton, in which autonomous reorganisation of its components followed a time course that could be programmed using five independent variables. The absolute configuration of the ligand binding sites controlled the sign and magnitude of the output signals, while initial TCA^−^ concentration, temperature and water content all influenced the rate of movement between sites. An excess of TCAH fuel allowed the start time of the motion to be delayed. The total operating time of the automaton was typically on the order of hundreds of seconds, a convenient timescale for continuous monitoring by optical techniques. The migration of the metal between stations and the factors controlling fuel decomposition are general and need not necessarily be limited to systems with a CD output; their highly programmable behaviour shows their potential to be used as controllers for more complex supramolecular assemblies.

## Experimental Section

**Representative sample preparation for a TCAH fuelled system**: An initial sample was prepared in a 1 mm path length quartz cuvette from Zn(BQPA) ⋅ 2ClO_4_ (20 μL, 2.50 mM in MeCN, 50 nmol), ligand **4** (20 μL, 2.50 mM in MeCN, 50 nmol), NEt_3_ (20 μL, 5.00 mM in MeCN, 100 nmol) and MeCN (120 μL). TCAH (20 μL, 5.00 mM in MeCN with 20 vol % H_2_O, 100 nmol) was added and the sample was quickly shaken for homogeneity, then placed into the CD spectrometer sample chamber (temperature set to 25 °C) within 10 seconds. The ellipticity output at 239.5 nm (θ_239.5_) was monitored over 20 minutes, with data points being taken every 10 seconds. See the Supporting Information for tabulated sample compositions for all TCAH fuelled experiments.

Synthetic procedures, characterisation of novel compounds (including ^1^H and ^13^C NMR spectra), miscellaneous CD data (base titrations and binding isotherms) and p*K*_a_ calculation methodology may be found in the Supporting Information.

## Conflict of interest

The authors declare no conflict of interest.

1

## Supporting information

As a service to our authors and readers, this journal provides supporting information supplied by the authors. Such materials are peer reviewed and may be re‐organized for online delivery, but are not copy‐edited or typeset. Technical support issues arising from supporting information (other than missing files) should be addressed to the authors.

Supporting Information

## Data Availability

The data that support the findings of this study are available in the supplementary material of this article.

## References

[1] S. Mann , Angew. Chem. Int. Ed. 2008, 47, 5306–5320; 10.1002/anie.200705538 18512208

[2] V. Balzani , M. Clemente-León , A. Credi , B. Ferrer , M. Venturi , A. H. Flood , J. F. Stoddart , Proc. Natl. Acad. Sci. USA 2006, 103, 1178–1183. 16432207 10.1073/pnas.0509011103 PMC1360556

[3] M. You , Y. Chen , X. Zhang , H. Liu , R. Wang , K. Wang , K. R. Williams , W. Tan , Angew. Chem. Int. Ed. 2012, 51, 2457–2460; 10.1002/anie.201107733 PMC3843772 22298502

[4] G. Ragazzon , M. Baroncini , S. Silvi , M. Venturi , A. Credi , Nat. Nanotechnol. 2015, 10, 70–75. 25420035 10.1038/nnano.2014.260

[5] J. C. M. Kistemaker , P. Štacko , J. Visser , B. L. Feringa , Nat. Chem. 2015, 7, 890–896. 26492009 10.1038/nchem.2362

[6] M. R. Wilson , J. Solà , A. Carlone , S. M. Goldup , N. Lebrasseur , D. A. Leigh , Nature 2016, 534, 235–240. 27279219 10.1038/nature18013

[7] E. Uhl , S. Thumser , P. Mayer , H. Dube , Angew. Chem. Int. Ed. 2018, 57, 11064–11068; 10.1002/anie.201804716 29932486

[8] J. Boekhoven , W. E. Hendriksen , G. J. M. Koper , R. Eelkema , J. H. Van Esch , Science 2015, 349, 1075–1079. 26339025 10.1126/science.aac6103

[9] S. Maiti , I. Fortunati , C. Ferrante , P. Scrimin , L. J. Prins , Nat. Chem. 2016, 8, 725–731. 27325101 10.1038/nchem.2511

[10] S. M. Morrow , I. Colomer , S. P. Fletcher , Nat. Commun. 2019, 10, 1011. 30824804 10.1038/s41467-019-08885-9 PMC6397266

[11] C. G. Pappas , P. K. Mandal , B. Liu , B. Kauffmann , X. Miao , D. Komáromy , W. Hoffmann , C. Manz , R. Chang , K. Liu , K. Pagel , I. Huc , S. Otto , Nat. Chem. 2020, 12, 1180–1186. 33219361 10.1038/s41557-020-00565-2

[12] M. De Poli , W. Zawodny , O. Quinonero , M. Lorch , S. J. Webb , J. Clayden , Science 2016, 352, 575–580. 27033546 10.1126/science.aad8352

[13] F. G. A. Lister , B. A. F. Le Bailly , S. J. Webb , J. Clayden , Nat. Chem. 2017, 9, 420–425.

[14] M. J. Langton , L. M. Scriven , N. H. Williams , C. A. Hunter , J. Am. Chem. Soc. 2017, 139, 15768–15773. 28876061 10.1021/jacs.7b07747

[15] E. Mattia , S. Otto , Nat. Nanotechnol. 2015, 10, 111–119. 25652169 10.1038/nnano.2014.337

[16] F. Lancia , A. Ryabchun , N. Katsonis , Nat. Chem. Rev. 2019, 3, 536–551.

[17] M. N. Tasbas , E. Sahin , S. Erbas-Cakmak , Coord. Chem. Rev. 2021, 443, 214039.

[18] M. N. Stojanovic , D. Stefanovic , Nat. Biotechnol. 2003, 21, 1069–1074. 12923549 10.1038/nbt862

[19] R. Pei , E. Matamoros , M. Liu , D. Stefanovic , M. N. Stojanovic , Nat. Nanotechnol. 2010, 5, 773–777. 20972436 10.1038/nnano.2010.194

[20] J. E. Poje , T. Kastratovic , A. R. Macdonald , A. C. Guillermo , S. E. Troetti , O. J. Jabado , M. L. Fanning , D. Stefanovic , J. Macdonald , Angew. Chem. Int. Ed. 2014, 53, 9222–9225; 10.1002/anie.201402698 25044570

[21] M. M. Wootten , B. A. F. Le Bailly , S. Tshepelevitsh , I. Leito , J. Clayden , Chem. Sci. 2022, 13, 2258–2269. 35310487 10.1039/d1sc06812a PMC8864710

[22] L. A. Joyce , M. S. Maynor , J. M. Dragna , G. M. Da Cruz , V. M. Lynch , J. W. Canary , E. V. Anslyn , J. Am. Chem. Soc. 2011, 133, 13746–13752. 21780788 10.1021/ja205775g PMC3179184

[23] L. A. Joyce , J. W. Canary , E. V. Anslyn , Chem. Eur. J. 2012, 18, 8064–8069. 22592912 10.1002/chem.201103592 PMC3416025

[24] R. Jastrzab , J. Coord. Chem. 2013, 66, 98–113.

[25] A. Klamt , J. Phys. Chem. 1995, 99, 2224–2235.

[26] A. Klamt , V. Jonas , T. Bürger , J. C. W. Lohrenz , J. Phys. Chem. A 1998, 102, 5074–5085.

[27] F. Eckert , A. Klamt , AIChE J. 2002, 48, 369–385.

[28] A. Kütt , T. Rodima , J. Saame , E. Raamat , V. Mäemets , I. Kaljurand , I. A. Koppel , R. Y. Garlyauskayte , Y. L. Yagupolskii , L. M. Yagupolskii , E. Bernhardt , H. Willner , I. Leito , J. Org. Chem. 2011, 76, 391–395. 21166439 10.1021/jo101409p

[29] F. H. Verhoek , J. Am. Chem. Soc. 1934, 56, 571–577.

[30] F. H. Verhoek , J. Am. Chem. Soc. 1945, 67, 1062–1064.

[31] G. A. Hall , F. H. Verhoek , J. Am. Chem. Soc. 1947, 69, 613–616.

[32] C. N. Cochran , F. H. Verhoek , J. Am. Chem. Soc. 1947, 69, 2987–2988.

[33] Y. Abe , H. Okamura , K. Nakazono , Y. Koyama , S. Uchida , T. Takata , Org. Lett. 2012, 14, 4122–4125. 22860717 10.1021/ol301771w

[34] S. Erbas-Cakmak , S. D. P. Fielden , U. Karaca , D. A. Leigh , C. T. McTernan , D. J. Tetlow , M. R. Wilson , Science 2017, 358, 340–343. 29051374 10.1126/science.aao1377

[35] C. Biagini , S. D. P. Fielden , D. A. Leigh , F. Schaufelberger , S. Di Stefano , D. Thomas , Angew. Chem. Int. Ed. 2019, 58, 9876–9880; 10.1002/anie.201905250 PMC6900173 31111628

[36] S. Choi , R. D. Mukhopadhyay , Y. Kim , I. C. Hwang , W. Hwang , S. K. Ghosh , K. Baek , K. Kim , Angew. Chem. Int. Ed. 2019, 58, 16850–16853; 10.1002/anie.201910161 31544353

[37] E. Olivieri , G. Quintard , J. V. Naubron , A. Quintard , J. Am. Chem. Soc. 2021, 143, 12650–12657. 34351739 10.1021/jacs.1c05183

[38] J. A. Berrocal , C. Biagini , L. Mandolini , S. Di Stefano , Angew. Chem. 2016, 128, 7111–7115; 10.1002/anie.201602594 27145060

[39] D. Del Giudice , E. Spatola , R. Cacciapaglia , A. Casnati , L. Baldini , G. Ercolani , S. Di Stefano , Chem. Eur. J. 2020, 26, 14954–14962. 32757429 10.1002/chem.202002574

[40] F. Rispoli , E. Spatola , D. Del Giudice , R. Cacciapaglia , A. Casnati , L. Baldini , S. Di Stefano , J. Org. Chem. 2022, 87, 3623–3629. 35196018 10.1021/acs.joc.2c00050 PMC8902750

[41] A. Ghosh , I. Paul , M. Schmittel , J. Am. Chem. Soc. 2021, 143, 5319–5323. 33787253 10.1021/jacs.1c01948

[42] F. Eckert , I. Leito , I. Kaljurand , A. Kütt , A. Klamt , M. Diedenhofen , J. Comput. Chem. 2009, 30, 799–810. 18727157 10.1002/jcc.21103

[43] TCAH stock solutions in neat anhydrous MeCN spontaneously decomposed with a half-life of approximately 3 h, even in the absence of base. Adding 20 vol % H_2_O to these solutions stabilised the TCAH sufficiently for our studies.

[44] This rate acceleration came at the cost of a weaker phosphate signal, decreasing from −3,800 (at 2 equiv.) to −2,100 deg dm^2^ mol^−1^ (at 5 equiv.), presumably due to TCA^−^ becoming increasingly competitive with the phosphate site for binding to [Zn(BQPA)]^2+^ at higher concentrations. The carboxylate signal was unaffected by TCA^−^ concentration.

[45] Higher temperatures led to weaker carboxylate signals, as though not all of the Zn^2+^ is arriving at the final destination. This may be rationalised by considering the entropy of different binding configurations. The strongest carboxylate signal would be observed when every [Zn(BQPA)]^2+^ binding site is occupied solely by carboxylate ligands. However, this configuration has a lower entropy than if some phosphate-bound [Zn(BQPA)]^2+^ is present i. e. if migration is incomplete. Therefore, the extent of [Zn(BQPA)]^2+^ migration decreases as temperature increases, as this is entropically more favourable, and results in a reduced carboxylate output. The same argument may be made for the decrease in phosphate magnitude with temperature, but with TCA^−^ being the competing anion.

[46] This lower concentration was used to minimise the change in [Zn(BQPA)]^2+^ and water concentration with each fuel pulse. The concentration change did not have a significant effect on the operating time of the automaton.

