# Supplementary material for: A Chemically Fuelled Molecular Automaton Displaying Programmed Migration of Zn2+ Between Alternative Binding Sites
Source: Chemistry. 2022 Aug 17;28(59):e202202247. doi: 10.1002/chem.202202247 (PMC9804598; doi:10.1002/chem.202202247)
Supplement: Supplementary file 1 — Supporting Information [file CHEM-28-0-s001.pdf]

Chemistry–A European Journal

Supporting Information

A Chemically Fuelled Molecular Automaton Displaying Programmed Migration of Zn^{2+} Between Alternative Binding Sites

Matthew M. Wootten, Sofja Tshepelevitsh, Ivo Leito, and Jonathan Clayden*

Table of Contents

S1 General information & Instrumentation ... 2

S2 Synthetic details ... 3

S2.1 General procedures ... 3

S2.2 Synthesis of test ligands **2a-c** ... 4

S2.3 Synthesis of test ligand **2d** ... 10

S2.4 Synthesis of test ligands **2e,f** ... 13

S2.5 Synthesis of carboxylate test ligand **3** ... 19

S2.6 Synthesis of pseudopeptide ligand **4** ... 22

S3 CD spectroscopy methodology ... 28

S3.1 Stock solution preparation ... 28

S3.2 Base titrations of ligands ... 28

S3.3 TCAH fuel experiments ... 31

S3.3.1 Varying initial TCA^- concentration (Fig. 5a) ... 31

S3.3.2 Varying the temperature (Fig. 5b) ... 31

S3.3.3 Varying water content (Fig. 5c) ... 32

S3.3.4 Varying initial TCAH concentration (Fig. 5d) ... 32

S3.3.5 Multiple fuel pulses (Fig. 5e) ... 33

S3.3.6 Assessing performance drift ... 33

S3.4 Binding constant measurements ... 34

S4 Calculation of pK_a values of ligands **2f** and **3** ... 37

S5 ^1H and ^{13}C NMR spectra of novel compounds ... 40

S6 References ... 70

S1 General information & Instrumentation

Air and/or moisture sensitive reactions were performed under a nitrogen atmosphere with glassware that was oven-dried or dried by heat gun, using standard anhydrous techniques. Air and/or moisture sensitive liquids and solutions were transferred via syringe through rubber septa.

All reagents were purchased from commercial suppliers and used as received, unless otherwise stated. Triethylamine was stored over KOH pellets. Anhydrous dichloromethane (DCM) and tetrahydrofuran (THF) were dried using an Anhydrous Engineering Grubbs-type solvent system. Anhydrous acetonitrile (MeCN) was purchased from Acros Organics and was measured to have a water content of 7.1 ppm by Karl Fischer titration (average of 3 measurements). Non-anhydrous solvents were purchased from commercial suppliers and used as received. P.E. refers to fractions of petroleum ether with boiling points in the range 40–60 °C.

Flash column chromatography was performed on silica gel (VWR 40-63 µm, 230-300 mesh). Analytical thin-layer chromatography (TLC) was performed on aluminium-backed silica (Merck 60 Å F_{254}), visualising using a UV lamp (254 nm), potassium permanganate or phosphomolybdic acid stain.

^{1}H and ^{13}C NMR spectra were acquired on Bruker (400 or 500 MHz) or Jeol ECS (400 MHz) spectrometers. Chemical shifts (δ) are reported in parts per million (ppm) and coupling constants (J) are reported in Hertz (Hz) to the nearest 0.1 Hz. ^{1}H-NMR spectra are referenced to the residual solvent peak ($CDCl_3$ 7.26, CD_3OD 3.31) and ^{13}C-NMR were referenced to the ^{13}C resonance of the solvent ($CDCl_3$ 77.16, CD_3OD 49.00). Multiplicities are reported as singlet (s), doublet (d), triplet (t), quartet (q), quintet (quint), septet (sept), multiplet (m), broad (br) or combinations thereof. Major and minor rotamers/diastereomers are denoted by the subscripts 'maj' and 'min'. ^{13}C resonances that are split multiple times due to rotamers, diastereomers and/or ^{31}P coupling are reported as multiplets.

Infrared spectra were recorded on a Perkin Elmer Spectrum Two spectrometer. Only major absorption maxima (ν_{max}) above 1000 cm^{-1} are reported and quoted in wavenumbers (cm^{-1}).

High-resolution ESI spectra were acquired on Bruker micrOTOF II or Thermo Scientific Orbitrap Elite spectrometers. High-resolution Nanospray spectra were acquired on a Waters Synapt G2S spectrometer with Advion Nanomate installed.

CD spectra were acquired on a JASCO J-815 CD spectrometer at the stated temperature and concentration using a 1 mm or 10 mm path length quartz cuvette. See section **S3** for detailed descriptions of sample preparation and experimental methods.

Zn(BQPA).2ClO_4 was prepared according our previous work.[1]

S2 Synthetic details

S2.1 General procedures

General Procedure A: Benzyl protection of carboxylic acids

K_2CO_3 (1.5 eq.) was added to a solution of carboxylic acid (1 eq.) in DMF (4 mL $mmol^{-1}$). Benzyl bromide (1.5 eq.) was added, and the mixture was stirred for 18 h at room temperature. Sat. NH_4Cl (4 mL $mmol^{-1}$) was added, and the biphasic mixture was extracted with Et_2O. The combined ethereal layers were washed with LiCl (5 wt% in H_2O) and brine, then dried (Na_2SO_4), filtered and concentrated under reduced pressure to give the crude product, which was purified by silica column chromatography.

General Procedure B: Phosphorodiamidite-alcohol coupling

Adapted from a literature procedure.[2]

5-(Ethylthio)-tetrazole (5-ETT, 1 eq.) was added to a solution of alcohol (1 eq.) and benzyl-*N*,*N*,*N′*,*N′*-tetraisopropylphosphorodiamidite **7** (1.1-1.2 eq.) in DCM (10 mL $mmol^{-1}$). The reaction mixture was stirred for 18 hours at room temperature and then quenched with sat. $NaHCO_3$ (10 mL $mmol^{-1}$). The biphasic mixture was extracted with DCM and the combined organic layers were washed with brine, dried (Na_2SO_4), filtered and concentrated under reduced pressure. The crude residue was purified by silica column chromatography to give the phosphoramidite product.

General Procedure C: Phosphoramidite-alcohol coupling and oxidation

Adapted from a literature procedure.[2]

5-(Ethylthio)-tetrazole (5-ETT, 1 eq.) was added to a solution of phosphoramidite (1 eq.) and alcohol (0.8-1.2 eq.) in DCM (10 mL $mmol^{-1}$). The mixture was stirred for 2-3 h (monitoring by TLC) at room temperature. *meta*-Chloroperbenzoic acid (1.4 eq.) was then added in portions, and the resulting solution was stirred for 1 h. The reaction mixture was washed with sat. $NaHCO_3$, 1 M HCl and brine, then dried (Na_2SO_4), filtered and concentrated under reduced pressure to give the crude product, which was purified by silica column chromatography.

General Procedure D: Deprotection of *O*-benzyl groups by catalytic hydrogenation

Pd/C (10% w/w, 10 mol%) was added to a solution of *O*-benzylated substrate (1 eq.) in methanol (10-20 mL $mmol^{-1}$). The mixture was placed under a hydrogen atmosphere (balloon pressure) and stirred for 24 h at room temperature (monitoring by TLC). When the reaction was complete the mixture was filtered through a pad of celite. The pad was washed with methanol, and the combined filtrates evaporated. Azeotropic removal of residual methanol with chloroform, followed by trituration with Et_2O, gave the deprotected product.

S2.2 Synthesis of test ligands **2a-c**

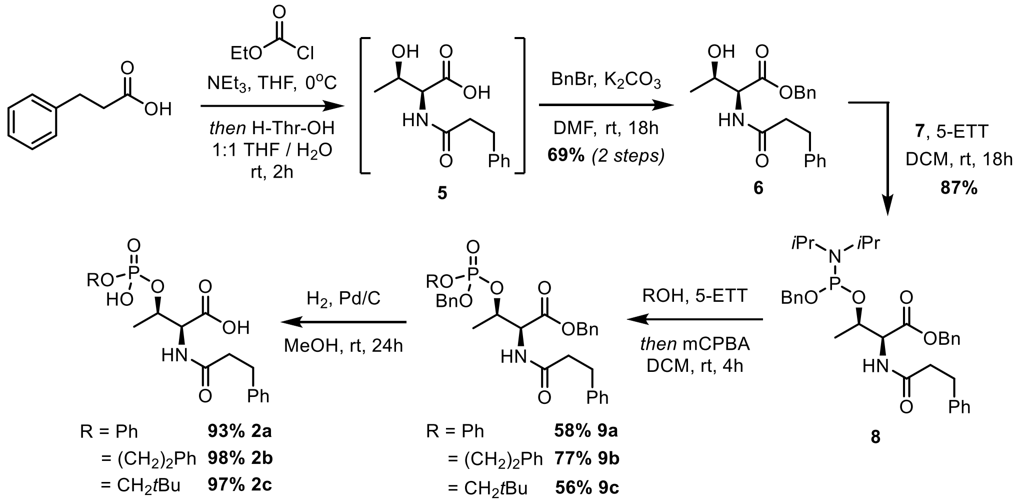

Scheme 1. Synthetic route to ligands **2a-c**

N-(3-Phenylpropanoyl)-L-threonine **5**

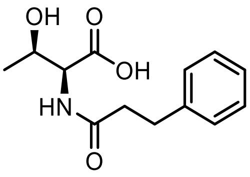

Prepared according to a modified literature procedure.[3]

Ethyl chloroformate (0.67 mL, 7.0 mmol) was added dropwise to a solution of 3-phenylpropionic acid (751 mg, 5.0 mmol) and triethylamine (2.1 mL, 15 mmol) in THF (10 mL) at 0 °C. The mixture was stirred at 0 °C for 1 hour, then a solution of L-threonine (893 mg, 7.5 mmol) in water (10 mL) was added. The mixture was stirred at room temperature for 1 hour, then the THF was evaporated under reduced pressure. The aqueous mixture was acidified to pH = 1-2 with aq. HCl (1 M), then extracted with EtOAc. The combined organic extracts were washed with brine, dried ($MgSO_4$), filtered and concentrated under reduced pressure to give the crude *N*-(3-phenylpropanoyl)-L-threonine **5** (1.24 g), which was used immediately in the next step without further purification.

Benzyl *N*-(3-phenylpropanoyl)-L-threoninate **6**

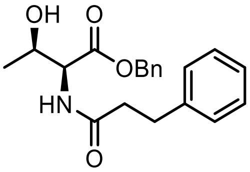

The crude *N*-(3-phenylpropanoyl)-L-threonine **5** (1.24 g, 4.9 mmol) was benzylated according to General Procedure A. The crude product was purified by silica column chromatography (1:1 EtOAc:P.E., R_f = 0.28), giving the title compound (1.18 g, 69% over 2 steps) as a colourless oil.

^{1}H NMR (CDCl$_3$, 400 MHz): δ_H 7.39–7.24 (m, 7H, ArH), 7.21–7.17 (m, 3H, ArH), 6.34 (d, *J* = 8.8, 1H, NH), 5.18 (d, *J* = 12.3, 1H, OC**H**HPh), 5.16 (d, *J* = 12.3, 1H, OCH**H**Ph), 4.64 (dd, *J* = 8.9, 2.5, 1H, C$^{\alpha}$H), 4.30 (qd, *J* = 6.4, 2.5, 1H, C$^{\beta}$H), 3.04–2.92 (m, 2H, $CH_2C\mathbf{H}_2Ph$), 2.65–2.52 (m, 2H, $C\mathbf{H}_2CH_2Ph$), 2.35 (br s, 1H, OH), 1.08 (d, *J* = 6.4, 3H, $C^{\gamma}H_3$)

^{13}C NMR (CDCl$_3$, 101 MHz): δ_C 172.9 (N**C**=O), 171.0 (C**C**=O), 140.7 (Ar), 135.3 (Ar), 128.8 (Ar), 128.6 (Ar), 128.6 (Ar), 128.4 (Ar), 128.3 (Ar), 126.4 (Ar), 68.1 (C$^{\beta}$H), 67.4 (O**C**H$_2$Ph), 57.4 (C$^{\alpha}$H), 38.2 ($\mathbf{C}H_2CH_2Ph$), 31.6 ($CH_2\mathbf{C}H_2Ph$), 20.0 ($C^{\gamma}H_3$)

FTIR (thin film): 3359 (br), 3071, 3035, 2976, 2936, 1739, 1645, 1524, 1196

HRMS (ESI$^+$): Calc. for $C_{20}H_{24}NO_4$ $[M+H]^+$ 342.1700, found 342.1717

Benzyl-*N*,*N*,*N′*,*N′*-tetraisopropylphosphorodiamidite **7**

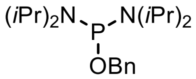

Prepared according to a modified literature procedure.[4] A solution of benzyl alcohol (0.54 mL, 5.0 mmol) and triethylamine (0.70 ml, 5.0 mmol) in Et_2O (5 ml) was added dropwise to a suspension of bis(diisopropylamino)chlorophosphine (1.33 g, 5.0 mmol) in Et_2O (20 ml) at 0 °C. The mixture was stirred at room temperature for 1.5 hours. The precipitate was filtered and washed with Et_2O, and the filtrate was concentrated under reduced pressure. The residue was triturated with hexane and filtered again, then concentrated to give the title compound (1.62 g, 96%) as a colourless oil, which was stored at -18 °C and used without further purification.

^{1}H NMR (400 MHz, CDCl$_3$): δ_H 7.41–7.30 (m, 4H, ArH), 7.26–7.22 (m, 1H, ArH) 4.66 (d, *J* = 7.4, 2H, PhC**H**$_2$), 3.58 (d sept, *J* = 11.1, 6.8, 4H, C**H**(CH$_3$)$_2$), 1.20 (d, *J* = 6.8, 12H, CH(C**H**$_3$)$_2$), 1.19 (d, *J* = 6.8, 12H, CH(C**H**$_3$)$_2$)

^{13}C NMR (101 MHz, CDCl$_3$): δ_C 140.7 (d, $^3J_{CP}$ = 10.3, Ar), 128.2 (Ar), 127.0 (Ar x2), 66.3 (d, $^2J_{CP}$ = 23.3, OCH$_2$), 44.6 (d, $^2J_{CP}$ = 12.4, NCH), 24.8 (d, $^3J_{CP}$ = 7.9, CH**C**H$_3$), 24.0 (d, $^3J_{CP}$ = 5.6, CH**C**H$_3$)

Spectroscopic data are consistent with those previously reported.[5]

Benzyl *O*-((benzyloxy)(diisopropylamino)phosphaneyl)-*N*-(3-phenylpropanoyl)-L-threoninate **8**

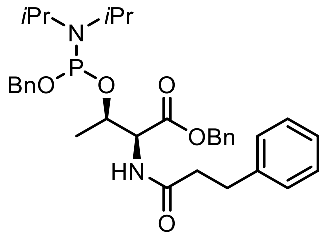

Prepared according to General Procedure B, using alcohol **6** (854 mg, 2.5 mmol) and **7** (1.02 g, 3.0 mmol). The crude product was purified by silica column chromatography (3:20:77 NEt_3:EtOAc:P.E., R_f = 0.32), giving the title compound (1.26 g, 87%) as a colourless oil. Isolated as a 40:60 mixture of diastereomers.

^{1}H NMR ($CDCl_3$, 400 MHz): δ_H 7.37–7.15 (m, 15H, ArH), 6.29 (d, *J* = 8.3, 0.6H, NH_{maj}), 6.16 (d, *J* = 9.2, 0.4H, NH_{min}), 5.20–4.99 (m, 2H, COC**H**$_2$Ph), 4.72–4.46 (m, 4H, POC**H**$_2$Ph + C$^\alpha$H + C$^\beta$H), 3.65–3.52 (m, 2H, C**H**(CH_3)$_2$), 3.01–2.87 (m, 2H, CH_2C**H**$_2$Ph), 2.55–2.38 (m, 2H, C**H**$_2$$CH_2$Ph), 1.21–1.14 (m, 15H, (C$^\gamma$H$_3$ + CH(C**H**$_3$)$_2$)

^{13}C NMR ($CDCl_3$, 101 MHz): δ_C 172.5 (m, NC=O), 170.4–170.3 (m, OC=O), 140.9–140.8 (m, Ar), 139.3 (d, $^3J_{C\text{-}P}$ = 7.6, Ar), 135.4 (m, Ar), 128.7–128.4 (m, Ar), 127.6 (Ar), 127.2 (Ar), 127.1 (Ar), 126.3 (m, Ar), 71.2 (d, $^2J_{C\text{-}P}$ = 15.3, C$^\beta$H$_{min}$), 70.0 (d, $^2J_{C\text{-}P}$ = 16.7, C$^\beta$H$_{maj}$), 67.4–67.3 (m, CO**C**H$_2$Ph), 65.5–64.9 (m, PO**C**H$_2$Ph), 57.7–57.4 (m, C$^\alpha$H), 43.3–43.1 (m, **C**H(CH_3)$_2$), 38.1–38.0 (m, **C**H$_2$$CH_2$Ph), 31.5 ($CH_2$**C**H$_2$Ph), 24.9–24.6 (CH(**C**H$_3$)$_2$), 19.8–19.5 (m, C$^\gamma$H$_3$)

FTIR (thin film): 3327 (br), 3035, 2967, 2936, 2868, 1746, 1681, 1497, 1183

HRMS (ESI$^+$): Calc. for $C_{33}H_{44}N_2O_5P$ $[M+H]^+$ 579.2982, found 579.2996

Benzyl *O*-((benzyloxy)(phenoxy)phosphoryl)-*N*-(3-phenylpropanoyl)-L-threoninate **9a**

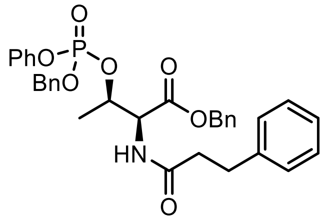

Prepared according to General Procedure C, using **8** (174 mg, 0.3 mmol) and phenol (34 mg, 0.36 mmol). The crude product was purified by silica column chromatography (45:55 EtOAc:P.E., R_f = 0.30), giving the title compound (102 mg, 58%) as a colourless oil. Isolated as a 40:60 mixture of diastereomers.

^{1}H NMR ($CDCl_3$, 400 MHz): δ_H 7.37–7.25 (m, 14H, ArH), 7.21–7.12 (m, 6H, ArH), 6.27 (d, *J* = 9.1, 0.6H, NH_{maj}), 6.23 (d, *J* = 9.1, 0.4H, NH_{min}), 5.17–5.04 (m, 4H, POC**H**$_2$Ph + C$^\beta$**H** + COC**H**HPh), 4.98–4.89 (m, 1H,

COCH**H**Ph), 4.83–4.79 (m, 1H, C$^{\alpha}$H), 3.02–2.91 (m, 2H, CH$_2$C**H**$_2$Ph), 2.64–2.50 (m, 2H, C**H**$_2$CH$_2$Ph), 1.28–1.22 (m, 3H, C$^{\gamma}$H$_3$)

^{13}C NMR (CDCl$_3$, 101 MHz): δ_C 172.6–172.5 (m, NC=O), 169.2 (m, OC=O), 150.5 (m, Ar), 140.6 (m, Ar), 135.4–135.3 (m, Ar), 135.0 (m, Ar), 129.8 (Ar), 128.8 (Ar), 128.7–128.4 (m, Ar), 128.1 (m, Ar), 126.3 (Ar), 125.4 (Ar), 120.2–120.1 (m, Ar), 76.5 (m, C$^{\beta}$H), 70.2 (m, PO**C**H$_2$Ph), 67.8 (m, CO**C**H$_2$Ph), 56.5– 56.4 (m, C$^{\alpha}$H), 38.0 (m, **C**H$_2$CH$_2$Ph), 31.4 (m, CH$_2$**C**H$_2$Ph), 18.5 (m, C$^{\gamma}$H$_3$)

FTIR (thin film): 3309 (br), 3062, 3030, 2936, 1746, 1677, 1490, 1276, 1207, 1008

HRMS (ESI$^+$): Calc. for $C_{33}H_{34}NNaO_7P$ [M+Na]$^+$ 610.1965, found 610.1961

Benzyl *O*-((benzyloxy)(phenethoxy)phosphoryl)-*N*-(3-phenylpropanoyl)-L-threoninate **9b**

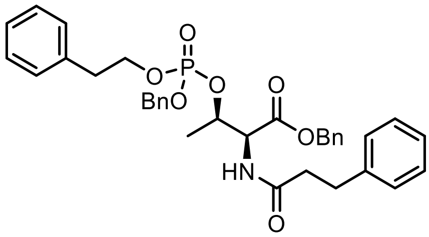

Prepared according to General Procedure C, using **8** (174 mg, 0.3 mmol) and phenethyl alcohol (43 μL, 0.36 mmol). The crude product was purified by silica column chromatography (45:55 EtOAc:P.E., R$_f$ = 0.21), giving the title compound (132 mg, 77%) as a colourless oil. Isolated as a mixture of diastereomers.

^{1}H NMR (CDCl$_3$, 400 MHz): δ_H 7.37–7.14 (m, 20H, ArH), 6.31–6.27 (m, 1H, NH), 5.17–5.14 (m, 1H, COC**H**HPh), 5.03–4.99 (m, 1H, COCH**H**Ph), 4.96–4.91 (m, 1H, C$^{\beta}$H), 4.90–4.87 (m, 2H, POC**H**$_2$Ph), 4.77–4.73 (m, 1H, C$^{\alpha}$H), 4.17–4.04 (m, 2H, OC**H**$_2$CH$_2$Ph), 3.01–2.94 (m, 2H, CCH$_2$C**H**$_2$Ph), 2.92–2.86 (m, 2H, OCH$_2$C**H**$_2$Ph), 2.63–2.50 (m, 2H, CC**H**$_2$CH$_2$Ph), 1.20–1.17 (m, 3H, C$^{\gamma}$H$_3$)

^{13}C NMR (CDCl$_3$, 101 MHz): δ_C 172.5 (N**C**=O), 169.4 (O**C**=O), 140.6 (Ar), 137.0 (m, Ar), 135.8–135.7 (m, Ar), 135.1 (Ar), 129.1 (Ar), 128.7–128.4 (m, Ar), 128.0–127.9 (m, Ar), 126.9 (Ar), 126.3 (Ar), 75.6–75.5 (m, C$^{\beta}$H), 69.4 (d, $^2J_{C\text{-}P}$ = 5.7, PO**C**H$_2$Ph), 68.3 (d, $^2J_{C\text{-}P}$ = 6.1, O**C**H$_2$CH$_2$Ph), 67.7 (m, CO**C**H$_2$Ph), 56.5 (d, $^3J_{CP}$ = 6.7, C$^{\alpha}$H), 38.0 (C**C**H$_2$CH$_2$Ph), 36.7 (d, $^3J_{CP}$ = 7.1, OCH$_2$**C**H$_2$Ph), 31.4 (CCH$_2$**C**H$_2$Ph), 18.4 (m, C$^{\gamma}$H$_3$)

FTIR (thin film): 3291 (br), 3066, 3026, 2958, 1746, 1675, 1497, 1265

HRMS (ESI$^+$): Calc. for $C_{35}H_{38}NNaO_7P$ [M+Na]$^+$ 638.2278, found 638.2255

Benzyl *O*-((benzyloxy)(neopentyloxy)phosphoryl)-*N*-(3-phenylpropanoyl)-L-threoninate **9c**

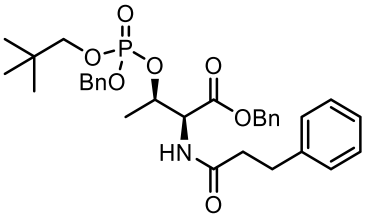

Prepared according to General Procedure C, using **8** (174 mg, 0.3 mmol) and neopentyl alcohol (39 μL, 0.36 mmol). The crude product was purified by silica column chromatography (40:60 EtOAc:P.E., R_f = 0.22), giving the title compound (98 mg, 56%) as a colourless oil. Isolated as a 45:55 mixture of diastereomers.

^{1}H NMR ($CDCl_3$, 400 MHz): δ_H 7.39–7.24 (m, 12H, ArH), 7.22–7.16 (m, 3H, ArH), 6.41 (d, *J* = 8.9, 0.45H, NH_{min}), 6.32 (d, *J* = 9.1, 0.55H, NH_{maj}), 5.21–5.14 (m, 1H, COC**H**HPh), 5.06–4.92 (m, 4H, COCH**H**Ph + POC**H**$_2$Ph + C$^\beta$H), 4.80–4.75 (m, 1H, C$^\alpha$H), 3.64–3.57 (m, 2H, C**H**$_2$C(CH$_3$)$_3$), 3.04–2.91 (m, 2H, CH$_2$C**H**$_2$Ph), 2.66–2.51 (m, 2H, C**H**$_2$CH$_2$Ph), 1.28–1.22 (m, 3H, C$^\gamma$H$_3$), 0.91–0.89 (m, 9H, C(C**H**$_3$)$_3$)

^{13}C NMR ($CDCl_3$, 101 MHz): δ_C 172.6–172.5 (m, N**C**=O), 169.4 (m, O**C**=O), 140.6 (Ar), 135.9–135.8 (m, Ar), 135.1–135.0 (m, Ar), 128.7–128.4 (m, Ar), 127.9 (m, Ar), 126.3 (Ar), 77.4–77.3 (**C**H$_2$C(CH$_3$)$_3$), 75.5–75.3 (m, C$^\beta$H), 69.4–69.3 (PO**C**H$_2$Ph), 67.8–67.70 (m, CO**C**H$_2$Ph), 56.6–56.5 (m, C$^\alpha$H), 38.1 (**C**H$_2$CH$_2$Ph), 32.2–32.1 (m, **C**(CH$_3$)), 31.5–31.4 (m, CH$_2$**C**H$_2$Ph), 26.0 (C(**C**H$_3$)), 18.5–18.4 (m, C$^\gamma$H$_3$)

FTIR (thin film): 3287 (br), 3066, 3035, 2954, 1747, 1681, 1263, 1009

HRMS (ESI$^+$): Calc. for C_{32}H40NNaO_7P $[M+Na]^+$ 604.2435, found 604.2413

O-(Hydroxy(phenoxy)phosphoryl)-*N*-(3-phenylpropanoyl)-L-threonine **2a**

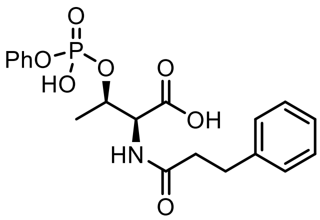

Prepared according to General Procedure D, using **9a** (87 mg, 0.15 mmol) in methanol (3 mL) to give the title compound (56 mg, 93%) as a colourless solid. Isolated as a mixture of diastereomers.

^{1}H NMR (CD_3OD, 400 MHz): δ_H 7.33 (t, *J* = 7.6, 2H, ArH), 7.27–7.12 (m, 8H, ArH), 5.00 (partially obscured by H_2O peak, C$^\beta$H), 4.66 (d, *J* = 13.2, 1H, C$^\alpha$H), 2.93 (t, *J* = 7.6, 2H, CH$_2$C**H**$_2$Ph), 2.69–2.59 (m, 2H, C**H**$_2$CH$_2$Ph), 1.24–1.19 (m, 3H, C$^\gamma$H$_3$)

^{13}C NMR (CD_3OD, 101 MHz): δ_C 175.8 (N**C**=O), 171.2 (C**C**=O), 152.8 (Ar), 142.1–142.0 (m, Ar), 130.6 (m, Ar), 129.4 (m, Ar), 127.2 (Ar), 125.60–125.5 (m, Ar), 121.3–121.2 (Ar), 76.2–75.9 ($C^{\beta}H$), 58.0–57.9 ($C^{\alpha}H$), 38.4–38.3 (**C**H_2CH_2Ph), 32.7 (m, CH_2**C**H_2Ph), 18.9–18.7 ($C^{\gamma}H_3$)

FTIR (thin film): 3274 (br), 3066, 3028, 2991, 2938, 1740, 1655, 1490, 1213, 1008

HRMS (Nanospray$^-$): Calc. for $C_{19}H_{21}NO_7P$ [M-H]$^-$ 406.1056, found 406.1047

O-(Hydroxy(phenethoxy)phosphoryl)-*N*-(3-phenylpropanoyl)-L-threonine **2b**

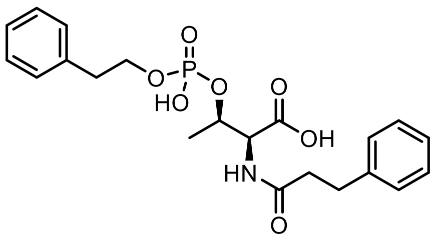

Prepared according to General Procedure D, using **9b** (82 mg, 0.13 mmol) in methanol (2.5 mL) to give the title compound (52 mg, 90%) as a colourless semi-solid.

^{1}H NMR (CD_3OD, 400 MHz): δ_H 7.29–7.13 (m, 10H, ArH), 4.92–4.87 (m, 1H partially obscured by H_2O signal, $C^{\beta}H$), 4.61–4.59 (m, 1H, $C^{\alpha}H$), 4.16–4.08 (m, 2H, OCH_2), 2.97–2.90 (m, 4H, CH_2 x2), 2.63 (td, J = 7.6, 3.3, 2H, CH_2), 1.18 (d, J = 6.3, 3H, $C^{\gamma}H_3$)

^{13}C NMR (CD_3OD, 101 MHz): δ_C 175.8 (N**C**=O), 172.3 (C**C**=O), 142.1 (Ar), 138.9 (Ar), 130.1 (Ar), 129.5 (Ar), 127.6 (Ar), 127.2 (Ar), 75.6 (d, $^2J_{CP}$ = 4.9, $C^{\beta}H$), 68.8 (d, $^2J_{CP}$ = 5.2, OCH_2), 57.8 (d, $^3J_{CP}$ = 8.4, $C^{\alpha}H$), 38.4 (CH_2), 37.7 (d, $^3J_{CP}$ = 7.3, CH_2), 32.7 (CH_2), 18.8 ($C^{\gamma}H_3$)

FTIR (thin film): 3281 (br), 3062, 3032, 2937, 1739, 1654, 1530, 1212, 1011

HRMS (Nanospray$^-$): Calc. for $C_{21}H_{25}NO_7P$ [M-H]$^-$ 434.1369, found 434.1362

O-(Hydroxy(neopentyloxy)phosphoryl)-N-(3-phenylpropanoyl)-L-threonine **2c**

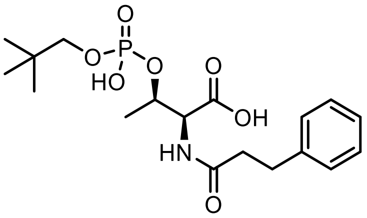

Prepared according to General Procedure D, using **9c** (87 mg, 0.15 mmol) in methanol (3 mL) to give the title compound (58 mg, 97%) as a colourless solid.

^{1}H NMR (CD_3OD, 400 MHz): δ_H 7.28–7.22 (m, 4H, ArH), 7.18–7.13 (m, 1H, ArH), 4.94 (partially obscured by H_2O peak, $C^{\beta}H$), 4.65–4.63 (m, 1H, $C^{\alpha}H$), 3.64–3.58 (m, 2H, OCH_2), 2.94 (t, J = 7.6, 2H, $CH_2\mathbf{CH_2}Ph$), 2.71–2.59 (m, 2H, $\mathbf{CH_2}CH_2Ph$), 1.23 (d, J = 6.1, 3H, $C^{\gamma}H_3$), 0.94 (s, 9H, $C(CH_3)_3$)

^{13}C NMR (CD_3OD, 101 MHz): δ_C 175.8 (N**C**=O), 172.3 (C**C**=O), 142.1 (Ar), 129.5 (Ar), 127.2 (Ar), 77.5 (d, $^2J_{CP}$ = 6.1, OCH_2), 75.5 (d, $^2J_{CP}$ = 4.9, $C^{\beta}H$), 57.9 (d, $^3J_{CP}$ = 7.9, $C^{\alpha}H$), 38.4 ($\mathbf{CH_2}CH_2Ph$), 32.8 (d, $^3J_{CP}$ = 8.1, $\mathbf{C}(CH_3)_3$), 32.8 ($CH_2\mathbf{CH_2}Ph$), 26.4 ($C(\mathbf{CH_3})_3$), 18.8 ($C^{\gamma}H_3$)

FTIR (thin film): 3277 (br), 3028, 2958, 2874, 1738, 1652, 1532, 1212, 1008

HRMS (Nanospray$^-$): Calc. for $C_{18}H_{27}NO_7P$ $[M-H]^-$ 400.1525, found 400.1521

S2.3 Synthesis of test ligand **2d**

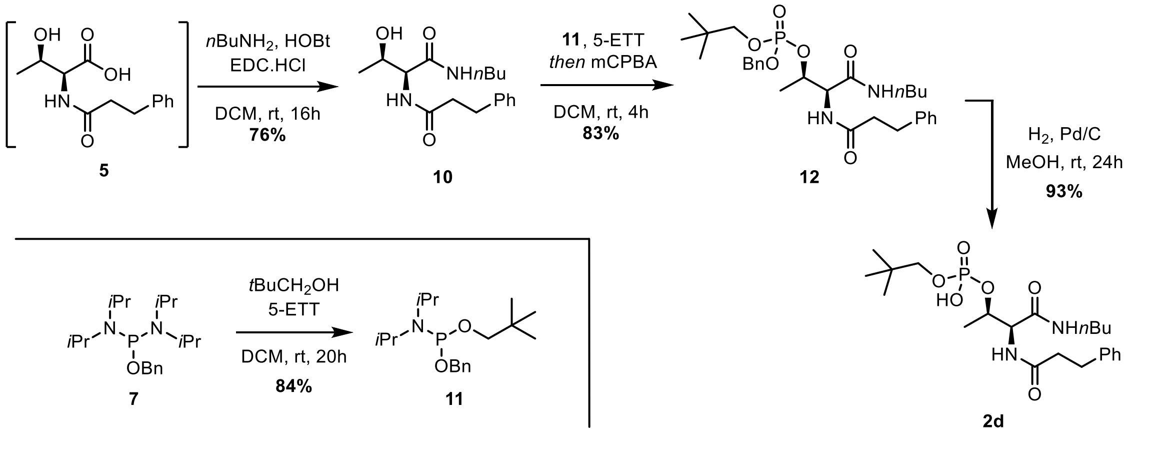

Scheme 2. Synthetic route to ligand **2d**

<u>$PhCH_2CH_2C(O)$-L-Thr-NH*n*Bu **10**</u>

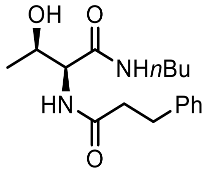

EDC.HCl (341 mg, 1.78 mmol) was added to a solution of crude *N*-(3-phenylpropanoyl)-L-threonine **5** (448 mg, 1.78 mmol), *n*-butylamine (0.16 mL, 1.6 mmol) and HOBt hydrate (218 mg, 1.6 mmol) in DCM (5 mL). The mixture was stirred at room temperature for 16 h, washed with aq. HCl (1 M), sat. $NaHCO_3$ and brine, then dried ($MgSO_4$), filtered and concentrated. The crude product was purified by silica column chromatography (70:30 EtOAc:P.E., R_f = 0.27) to give the title compound (373 mg, 76%) as a colourless solid.

^{1}H NMR (400 MHz, $CDCl_3$): δ_H 7.29–7.25 (m, 2H, ArH), 7.21–7.16 (m, 3H, ArH), 6.76 (t, *J* = 5.9, 1H, N**H**CH_2), 6.57 (d, *J* = 7.5, 1H, NH), 4.31–4.26 (m, 2H, $C^{\alpha}H$ + $C^{\beta}H$), 3.98 (br s, 1H, OH), 3.26–3.11 (m, 2H, NHC**H**$_2$), 2.96 (t, *J* = 7.6, 2H, CH_2C**H**$_2$Ph), 2.58 (t, *J* = 7.6, 2H, C**H**$_2CH_2$Ph), 1.48–1.41 (m, 2H, $NHCH_2$C**H**$_2$), 1.36–1.27 (m, 2H, C**H**$_2CH_3$), 1.00 (d, *J* = 6.4, 3H, $C^{\gamma}H_3$), 0.91 (t, *J* = 7.3, 3H, CH_2C**H**$_3$)

^{13}C NMR (101 MHz, $CDCl_3$): δ_C 173.4 (C=O), 171.2 (C=O), 140.4 (Ar), 128.7 (Ar), 128.4 (Ar), 126.5 (Ar), 66.4 ($C^{\beta}H$), 56.6 ($C^{\alpha}H$), 39.3 ($NHCH_2$), 38.2 (**C**H_2CH_2Ph), 31.7 (CH_2**C**H_2Ph), 31.5 ($NHCH_2$**C**H_2), 20.2 (**C**H_2CH_3), 18.0 ($C^{\gamma}H_3$), 13.8 (CH_2**C**H_3)

FTIR (thin film): 3288 (br), 2961, 2929, 2871, 1634, 1546

HRMS (ESI$^+$): Calc. for $C_{17}H_{26}N_2NaO_3$ $[M+Na]^+$ 329.1836, found 329.1825

<u>*O*-Benzyl-*O′*-neopentyl-*N,N*-diisopropylphosphoramidite **11**</u>

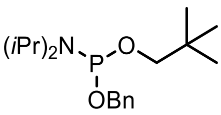

5-(Ethylthio)-tetrazole (182 mg, 1.4 mmol) was added to a solution of **7** (1.04 g, 3.08 mmol) and neopentyl alcohol (247 mg, 2.8 mmol) in DCM (11 mL). The mixture was stirred at room temperature for 20 hours, then washed with $NaHCO_3$ and brine, then dried (Na_2SO_4), filtered and concentrated. The crude product was purified by silica column chromatography (1:19 NEt_3:P.E.), giving the title compound (765 mg, 84%) as a colourless oil.

^{1}H NMR (400 MHz, $CDCl_3$): δ_H 7.39–7.30 (m, 4H, ArH), 7.27–7.22 (m, 1H, ArH), 4.76 (dd, *J* = 12.8, 8.3, 1H, OC**H**HPh), 4.67 (dd, *J* = 12.8, 8.4, 1H, OCH**H**Ph), 3.73–3.60 (m, 2H, C**H**$(CH_3)_2$), 3.34 (dd, *J* = 9.5, 6.1, 1H, OC**H**H*t*Bu), 3.27 (dd, *J* = 9.5, 6.4, 1H, OCH**H***t*Bu), 1.22–1.19 (m, 12H, CH(C**H**$_3)_2$), 0.92 (s, 9H, $C(CH_3)_3$)

^{13}C NMR (101 MHz, CDCl$_3$): δ_C 140.0 (d, $^3J_{CP}$ = 7.1, Ar), 128.3 (Ar), 127.2 (Ar), 127.0 (Ar), 74.1 (d, $^2J_{CP}$ = 14.9, O**C**H$_2$*t*Bu), 65.2 (d, $^2J_{CP}$ = 17.5, O**C**H$_2$Ph), 43.1 (d, $^2J_{CP}$ = 12.4, **C**H(CH$_3$)$_2$), 32.6 (d, $^3J_{CP}$ = 7.9, **C**(CH$_3$)$_3$), 26.7 (C(**C**H$_3$)$_3$), 24.8–24.7 (m, CH(**C**H$_3$)$_2$)

FTIR (thin film): 2963, 2933, 2867, 1362, 1009

HRMS (Nanospray$^+$): Calc. for $C_{18}H_{33}NO_2P$ [M+H]$^+$ 326.2249, found 326.2248

PhCH$_2$CH$_2$C(O)-L-Thr(PO(OBn)(OCH$_2$*t*Bu))-NHBu **12**

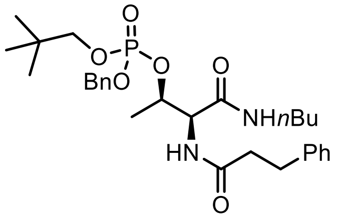

Prepared according to General Procedure C, using alcohol **10** (92 mg, 0.3 mmol) and phosphoramidite **11** (107 mg, 0.33 mmol). The crude product was purified by silica column chromatography (40:60 to 100:0 EtOAc:P.E., R_f = 0.27 at 50:50) to give the title compound (136 mg, 83%) as a colourless semi-solid. Isolated as a 55:45 mixture of diastereomers.

^{1}H NMR (CDCl$_3$, 400 MHz): δ_H 7.43–7.30 (m, 5H, ArH), 7.28–7.23 (m, 2H, ArH), 7.20–7.14 (m, 3H, ArH), 6.85–6.82 (m, 1H, NH), 6.60 (t, *J* = 5.8, 0.55H, N**H**CH$_{2maj}$), 6.51 (t, *J* = 5.7, 0.45H, N**H**CH$_{2min}$), 5.14–5.04 (m, 2H, OC**H**$_2$Ph), 4.89–4.80 (m, 1H, C$^\beta$H), 4.61–4.53 (m, 1H, C$^\alpha$H), 3.74–3.58 (m, 2H, C**H**$_2$C(CH$_3$)$_3$), 3.23–3.08 (m, 2H, NHC**H**$_2$), 2.96 (q, *J* = 7.5, 2H, CH$_2$C**H**$_2$Ph), 2.60–2.51 (m, 2H, C**H**$_2$CH$_2$Ph), 1.45–1.36 (m, 2H, NHCH$_2$C**H**$_2$), 1.34–1.24 (m, 2H, C**H**$_2$CH$_3$), 1.19 (t, *J* = 6.7, 3H, C$^\gamma$H$_3$), 0.92–0.86 (m, 12H, C(CH$_3$)$_3$ + CH$_2$C**H**$_3$)

^{13}C NMR (CDCl$_3$, 101 MHz): δ_C 172.4 (C=O), 168.0 (C=O$_{min}$), 167.9 (C=O$_{maj}$), 140.6 (Ar), 136.0–135.8 (m, Ar), 128.7 (Ar), 128.6 (m, Ar), 128.4 (Ar), 128.2 (Ar), 128.0 (Ar), 126.4 (m, Ar), 77.6–77.5 (m, **C**H$_2$C(CH$_3$)$_3$), 74.4–74.2 (m, C$^\beta$H), 69.9–69.6 (m, O**C**H$_2$Ph), 56.9–56.7 (m, C$^\alpha$H), 39.5 (NH**C**H$_2$), 38.1 (**C**H$_2$CH$_2$Ph), 32.2–32.1 (m, **C**(CH$_3$)$_3$), 31.6–31.5 (m, CH$_2$**C**H$_2$Ph + NHCH$_2$**C**H$_2$), 26.1 (C(**C**H$_3$)$_{3,min}$), 26.0 (C(**C**H$_3$)$_{3,maj}$), 20.1 (**C**H$_2$CH$_3$), 17.6–17.4 (C$^\gamma$H$_3$), 13.8 (CH$_2$**C**H$_3$)

FTIR (thin film): 3288 (br), 2958, 2934, 2872, 1644, 1256, 1007

HRMS (ESI$^+$): Calc. for $C_{29}H_{44}N_2O_6P$ [M+H]$^+$ 547.2932, found 547.2924

PhCH$_2$CH$_2$C(O)-L-Thr(PO$_2$H(OCH$_2$*t*Bu))-NHBu **2d**

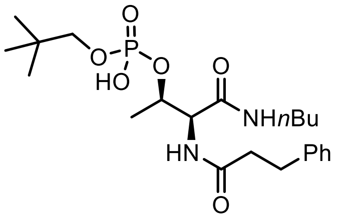

Prepared according to General Procedure D, using **12** (88 mg, 0.16 mmol) in MeOH (2 mL). The title compound (68 mg, 93%) was obtained as a colourless semi-solid.

^{1}H NMR (CDCl$_3$, 400 MHz): δ_H 7.30–7.25 (m, 2H, ArH), 7.21–7.17 (m, 3H, ArH), 6.95 (d, *J* = 7.6, 1H, NH), 6.88 (t, *J* = 5.5, 1H, NH), 6.41 (br s, 2H, OH + H_2O fast exchange), 4.85–4.77 (m, 1H, C$^\beta$H), 4.60 (dd, *J* = 7.6, 3.7, 1H, C$^\alpha$H), 3.71–3.64 (m, 2H, OCH$_2$), 3.29–3.21 (m, 1H, NHCH**H**), 3.18–3.09 (m, 1H, NHCH**H**), 2.96 (t, *J* = 7.6, 2H, CH$_2$**CH$_2$**Ph), 2.66–2.56 (m, 2H, **CH$_2$**CH$_2$Ph), 1.47–1.40 (m, 2H, NHCH$_2$**CH$_2$**), 1.35–1.26 (m, 2H, C**H$_2$**CH$_3$), 1.16 (d, *J* = 6.4, 3H, C$^\gamma$H$_3$), 0.94 (s, 9H, C(CH$_3$)$_3$), 0.90 (t, *J* = 7.3, 3H, CH$_2$C**H$_3$**)

^{13}C NMR (CDCl$_3$, 125 MHz): δ_C 173.3 (C=O), 169.0 (C=O), 140.4 (m, Ar), 128.8 (Ar), 128.5 (Ar), 126.6 (Ar), 73.0–72.8 (m, C$^\beta$H), 56.5 (C$^\alpha$H), 77.3 (partially obscured by solvent peak, OCH$_2$), 39.7 (NHCH$_2$), 38.1 (**C**H$_2$CH$_2$Ph), 32.2 (d, $^3J_{CP}$ = 7.6, **C**(CH$_3$)$_3$), 31.7 (CH$_2$**C**H$_2$Ph), 31.4 (NHCH$_2$**C**H$_2$), 26.1 (C(**C**H$_3$)$_3$), 20.1 (**C**H$_2$CH$_3$), 17.5 (m, C$^\gamma$H$_3$), 13.8 (CH$_2$**C**H$_3$)

FTIR (thin film): 3288 (br), 2958, 2934, 2872, 1642, 1536, 1007

HRMS (Nanospray$^-$): Calc. for $C_{22}H_{36}N_2O_6P$ [M-H]$^-$ 455.2311, found 455.2323

S2.4 Synthesis of test ligands **2e,f**

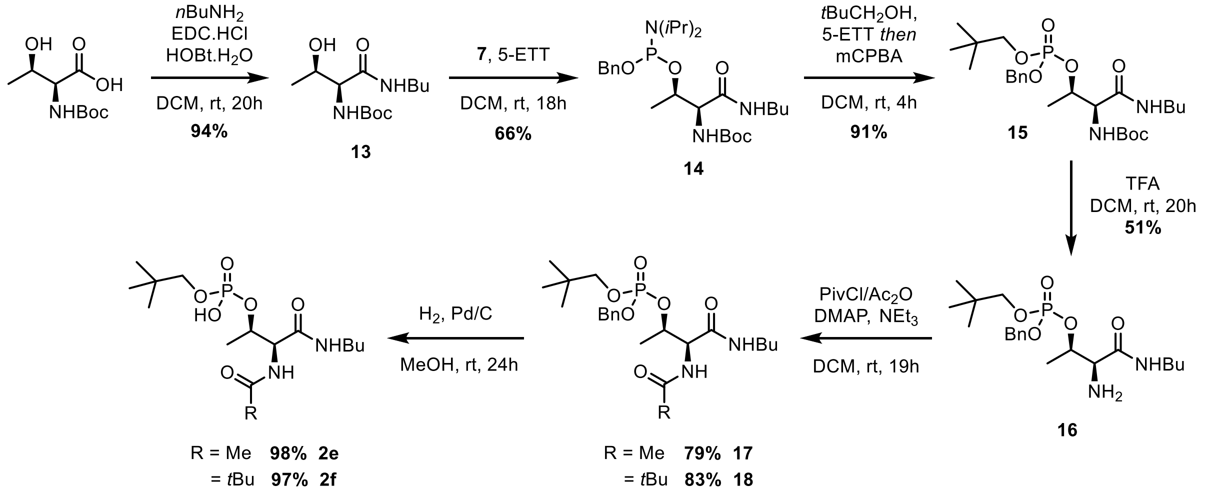

Scheme 3. Synthetic route to ligands **2e,f**

Boc-L-Thr-NHBu **13**

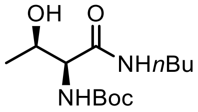

EDC.HCl (1.05 g, 5.5 mmol) was added to a solution of Boc-Thr-OH (1.21 g, 5.5 mmol), *n*-butylamine (0.49 mL, 5.0 mmol) and HOBt.H_2O (676 mg, 5.0 mmol) in DCM (40 mL) at 0 °C. After stirring at room temperature for 20 hours, the mixture was washed with sat. $NaHCO_3$, aq. HCl (1 M) and brine, then dried ($MgSO_4$), filtered and concentrated under reduced pressure. The crude product was purified by silica column chromatography (2:1 EtOAc:P.E., R_f = 0.23) to give the title compound (1.27 g, 94%) as a colourless solid.

^{1}H NMR (400 MHz, $CDCl_3$): δ_H 6.68 (br s, 1H, NH), 5.54 (d, *J* = 8.2, 1H, NH), 4.36–4.31 (m, 1H, $C^\beta H$), 3.98 (dd, *J* = 8.2, 2.2, 1H, $C^\alpha H$), 3.70 (br s, 1H, OH), 3.31–3.17 (m, 2H, NCH_2), 1.51–1.41 (m, 11H, CH_2 + $C(CH_3)_3$), 1.37–1.28 (m, 2H, CH_2), 1.16 (d, *J* = 6.5, 3H, $C^\gamma H_3$), 0.90 (t, *J* = 7.3, 3H, CH_3)

^{13}C NMR (101 MHz, $CDCl_3$): δ_C 171.8 (C**C**=O), 156.8 (O**C**=O), 80.5 (**C**$(CH_3)_3$), 66.8 ($C^\beta H$), 58.0 ($C^\alpha H$), 39.3 (NCH_2), 31.6 (CH_2), 28.4 (C(**C**$H_3)_3$), 20.1 (CH_2), 18.4 ($C^\gamma H_3$), 13.8 (CH_3)

FTIR (thin film): 3320 (br), 2966, 2933, 2874, 1694, 1648, 1500, 1366, 1163

HRMS (ESI$^+$): Calc. for $C_{13}N_{26}N_2NaO_4$ $[M+Na]^+$ 297.1785, found 297.1777

Boc-L-Thr(P(N*i*Pr_2)OBn)-NHBu **14**

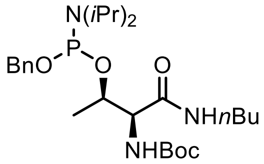

Prepared according to General Procedure B using alcohol **13** (1.00 g, 3.64 mmol) and **7** (1.48 g, 4.37 mmol) in DCM (36 mL). The crude product was purified by silica column chromatography (5:5:90 NEt_3:EtOAc:P.E., R_f = 0.37), giving the title compound (1.24 g, 66%) as a colourless oil. Isolated as a 2:1 mixture of diastereomers.

^{1}H NMR (400 MHz, $CDCl_3$): δ_H 7.37–7.24 (m, 5H, ArH), 6.62 (br s, 0.67H, NH_{maj}), 6.43 (br s, 0.33H, NH_{min}), 5.60 (br s, 0.67H, NH_{maj}), 5.42 (br s, 0.33H, NH_{min}), 4.80–4.70 (m, 1H, C**H**HPh), 4.65–4.56 (m, 1H, CH**H**Ph), 4.52–4.45 (m, 1H, $C^\beta H$), 4.25–4.09 (m, 1H, $C^\alpha H$), 3.69–3.56 (m, 2H, C**H**$(CH_3)_2$), 3.31–3.11 (m, 1.33H, $NCH_{2,maj}$), 3.04–2.96 (m, 0.67H, $NCH_{2,min}$), 1.46–1.44 (m, 9H, $C(CH_3)_3$), 1.36–1.23 (m, 4H, NCH_2C**H**$_2$ + C**H**$_2CH_3$), 1.22–1.14 (m, 15H, CH(C**H**$_3)_3$ + $C^\gamma H_3$), 0.89 (t, *J* = 7.3, 1H, CH_2C**H**$_{3,min}$), 0.84 (t, *J* = 7.2, 2H, CH_2C**H**$_{3,maj}$)

^{13}C NMR (125 MHz, $CDCl_3$): δ_C 169.5 (C**C**=O_{min}), 168.8 (C**C**=O_{maj}), 155.8 (OC=O_{min}), 155.5 (OC=O_{maj}), 139.4–139.0 (m, Ar), 128.5 (Ar), 127.7 (Ar), 127.5 (Ar), 127.1 (Ar), 78.0 (**C**$(CH_3)_{3,min}$), 79.7 (**C**$(CH_3)_{3,maj}$),

70.5–69.9 (m, C$^{\beta}$H), 65.6–65.4 (m, **C**H_2Ph), 59.0 (C$^{\alpha}$H$_{min}$), 58.0 (C$^{\alpha}$H$_{maj}$), 43.4–43.2 (m, **C**H$(CH_3)_2$), 39.4 (NCH$_{2,min}$), 39.3 (NCH$_{2,maj}$), 31.7 (CH$_{2,min}$), 31.5 (CH$_{2,maj}$), 28.5 (C(**C**H$_3$)$_{3,maj}$), 28.4 (C(**C**H$_3$)$_{3,min}$), 24.9–24.6 (m, CH(**C**H$_3$)$_2$), 20.2 (CH$_{2,min}$), 20.1 (CH$_{2,maj}$), 17.9–17.5 (m, C$^{\gamma}$H$_3$), 13.9 (CH$_{3,min}$), 13.8 (CH$_{3,maj}$)

FTIR (thin film): 3330 (br), 2965, 2932, 2872, 1716, 1662, 1168

HRMS (Nanospray$^+$): Calc. for $C_{26}H_{47}N_3O_5P$ $[M+H]^+$ 512.3253, found 512.3259

Boc-L-Thr(PO(OBn)(OCH$_2$*t*Bu))-NHBu **15a**

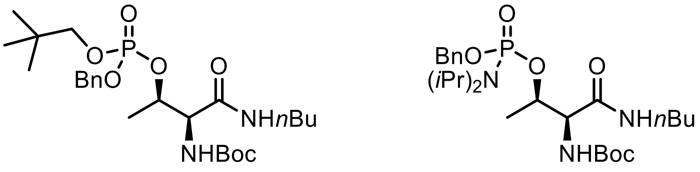

15a **15b**

Prepared according to General Procedure C using **14** (1.18 g, 2.3 mmol) and neopentyl alcohol (169 mg, 1.9 mmol). The crude product was purified by silica column chromatography (1:1 EtOAc:P.E., R$_f$ = 0.22), giving the title compound (897 mg, 91%) as a colourless oil. Isolated as a 2:1 mixture of diastereomers. **15b** was also detected as an inseparable minor impurity.

^{1}H NMR (400 MHz, CDCl$_3$,): δ_H 7.42–7.28 (m, 5H, ArH), 6.60 (br s, 0.33H, NH$_{min}$), 6.49 (br s, 0.67H, NH$_{maj}$), 5.57 (t, *J* = 9.6, 1H, N**H**CH$_2$), 5.12–5.02 (m, 2H, OC**H**$_2$Ph), 4.97–4.87 (m, 1H, C$^{\beta}$H), 4.32–4.19 (m, 1H, C$^{\alpha}$H), 3.70–3.59 (m, 2H, OC**H**$_2$*t*Bu), 3.28–3.10 (m, 2H, NHC**H**$_2$), 1.46–1.39 (m, 11H, $C(CH_3)_3$ + CH$_2$), 1.33–1.25 (m, 5H, C$^{\gamma}$H$_3$ + CH$_2$), 0.89–0.84 (m, 12H, $C(CH_3)_3$ + CH$_3$)

^{13}C NMR (101 MHz, CDCl$_3$,): δ_C 168.4 (C**C**=O), 155.7 (OC=O), 135.9 (d, $^{3}J_{CP}$ = 7.0, Ar), 128.7–128.6 (m, Ar), 128.1 (Ar), 128.0 (Ar), 80.3 (O**C**(CH$_3$)$_3$), 77.3 (d, $^{2}J_{CP}$ = 6.6, O**C**H$_2$*t*Bu), 75.0–74.9 (m, C$^{\beta}$H), 69.6–69.4 (m, O**C**H$_2$Ph), 58.3–58.2 (C$^{\alpha}$H), 39.4 (NHCH$_2$), 32.2–32.1 (m, C**C**(CH$_3$)$_3$), 31.6 (m, CH$_2$), 28.3 (OC(**C**H$_3$)$_3$), 26.0 (CC(**C**H$_3$)$_3$), 20.1 (CH$_2$), 17.7–17.6 (m, C$^{\gamma}$H$_3$), 13.8 (CH$_3$)

FTIR (thin film): 3299 (br), 2959, 2935, 2873, 1716, 1667, 1248, 1168, 1009

HRMS (ESI$^+$): Calc. for $C_{25}H_{43}N_2NaO_7P$ $[M+Na]^+$ 537.2700, found 537.2708

H-L-Thr(PO(OBn)(OCH$_2$*t*Bu))-NHBu **16**

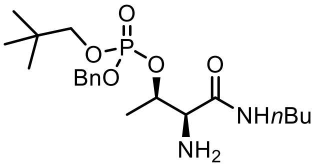

Trifluoroacetic acid (1.29 mL, 16.7 mmol) was added to a solution of **15a** (859 mg, 1.67 mmol) in DCM (16 mL). The mixture was stirred at room temperature for 20 hours, then the volatiles were evaporated under reduced pressure. The residue was redissolved in EtOAc and washed with sat. $NaHCO_3$ (aq.) and brine, then dried (Na_2SO_4), filtered and concentrated under reduced pressure to give the crude free amine, which was purified by silica column chromatography (1:19 MeOH:EtOAc, R_f = 0.17) to give the title compound (355 mg, 51%) as a colourless oil that crystallised on standing. Isolated as a mixture of diastereomers.

^{1}H NMR (400 MHz, CDCl$_3$): δ_H 7.39–7.27 (m, 6H, ArH + N**H**CH$_2$), 5.09–4.99 (m, 2H, OC**H**$_2$Ph), 4.98–4.90 (m, 1H, C$^\beta$H), 3.66–3.57 (m, 2H, C**H**$_2$C(CH$_3$)$_3$), 3.30–3.09 (m, 3H, C$^\alpha$H + NHC**H**$_2$), 1.49–1.40 (m, 2H, NCH$_2$C**H**$_2$), 1.39–1.36 (m, 3H, C$^\gamma$H$_3$), 1.34–1.24 (m, 2H, C**H**$_2$CH$_3$), 0.91–0.84 (m, 12H, C(C**H**$_3$)$_3$ + CH$_2$C**H**$_3$)

^{13}C NMR (101 MHz, CDCl$_3$): δ_C 171.2 (C=O), 136.2 (d, *J* = 7.1, Ar), 128.6–128.5 (m, Ar), 128.0 (Ar), 127.9 (Ar), 77.2–77.1 (m partially obscured by solvent peak, **C**H$_2$C(CH$_3$)$_3$), 76.4–76.2 (m, C$^\beta$H), 69.3–69.2 (m, O**C**H$_2$Ph), 59.5–59.4 (m, C$^\alpha$H), 39.1 (NHCH$_2$), 32.1 (d, *J* = 8.1, **C**(CH$_3$)$_3$), 31.7 (m, NHCH$_2$**C**H$_2$), 26.0 (C(**C**H$_3$)$_3$), 20.2 (m, **C**H$_2$CH$_3$), 18.5–18.4 (m, C$^\gamma$H$_3$), 13.8 (m, CH$_2$**C**H$_3$)

FTIR (thin film): 3312 (br), 2958, 2931, 2872, 1660, 1256

HRMS (ESI$^+$): Calc. for $C_{20}H_{36}N_2O_5P$ [M+H]$^+$ 415.2356, found 415.2355

Ac-L-Thr(PO(OBn)(OCH$_2$*t*Bu))-NHBu **17**

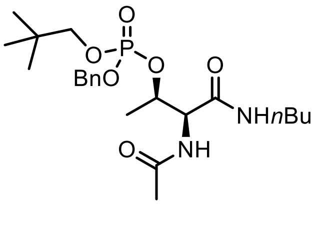

Acetic anhydride (21 µL, 0.22 mmol) was added dropwise to a solution of **16** (83 mg, 0.2 mmol), NEt$_3$ (42 µL, 0.3 mmol) and DMAP (5 mg, 0.04 mmol) in DCM (2 mL). After stirring at room temperature for 19 h, the mixture was diluted with EtOAc, washed with aq. HCl (1 M) and brine, then dried ($MgSO_4$), filtered and concentrated under reduced pressure. The crude product was purified by silica column chromatography (EtOAc, R_f = 0.27) to give the title compound (72 mg, 79%) as a colourless oil. Isolated as a mixture of diastereomers.

^{1}H NMR (CDCl$_3$, 400 MHz): δ_H 7.41–7.29 (m, 5H, ArH), 6.98–6.84 (m, 2H, NH x2), 5.12–5.05 (m, 2H, OC**H**$_2$Ph), 4.89–4.82 (m, 1H, C$^\beta$H), 4.64–4.55 (m, 1H, C$^\alpha$H), 3.73–3.57 (m, 2H, C**H**$_2$C(CH$_3$)$_3$), 3.28–3.12 (m, 2H, NHC**H**$_2$), 2.02–1.97 (m, 3H, CH$_3$^{Ac}), 1.48–1.40 (m, 2H, NHCH$_2$C**H**$_2$), 1.35–1.26 (m, 5H, C**H**$_2$CH$_3$ + C$^\gamma$H$_3$), 0.91–0.85 (m, 12H, C(C**H**$_3$)$_3$ + CH$_2$C**H**$_3$)

^{13}C NMR (CDCl$_3$, 125 MHz): δ_C 170.6 (C=O), 168.2 (C=O), 135.9–135.8 (m, Ar), 128.7–128.6 (m, Ar), 128.0 (Ar), 127.9 (Ar), 77.5–77.4 (m, **C**H$_2$C(CH$_3$)$_3$), 74.7–74.6 (m, C$^\beta$H), 69.7–69.5 (m, O**C**H$_2$Ph), 56.9 (C$^\alpha$H), 39.4 (NHCH$_2$), 32.1–32.0 (m, **C**(CH$_3$)$_3$), 31.4 (m, NCH$_2$**C**H$_2$), 26.0 (m, C(**C**H$_3$)$_3$), 23.1 (CH$_3$^{Ac}), 20.0 (**C**H$_2$CH$_3$), 17.9–17.7 (m, C$^\gamma$H$_3$), 13.7 (CH$_2$**C**H$_3$)

FTIR (thin film): 3286 (br), 2958, 2933, 2873, 1649, 1256, 1007

HRMS (ESI$^+$): Calc. for $C_{22}H_{37}N_2NaO_6P$ [M+Na]$^+$ 479.2281, found 279.2283

Piv-L-Thr(PO(OBn)(OCH$_2$*t*Bu))-NHBu **18**

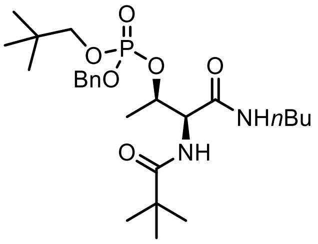

Pivaloyl chloride (27 µL, 0.22 mmol) was added dropwise to a solution of **16** (83 mg, 0.2 mmol), NEt$_3$ (42 µL, 0.3 mmol) and DMAP (5 mg, 0.04 mmol) in DCM (2 mL). After stirring at room temperature for 19 h, the mixture was diluted with EtOAc, washed with aq. HCl (1 M) and brine, then dried (MgSO$_4$), filtered and concentrated under reduced pressure. The crude product was purified by silica column chromatography (1:1 EtOAc:P.E., R_f = 0.20) to give the title compound (83 mg, 83%) as a colourless oil. Isolated as a 40:60 mixture of diastereomers.

^{1}H NMR (CDCl$_3$, 400 MHz): δ_H 7.40–7.27 (m, 5H, ArH), 7.01 (d, J = 7.1, 0.4H, NH$_{min}$), 6.98 (d, J = 7.2, 0.6H, NH$_{maj}$), 6.92 (t, J = 5.7, 0.4H, N**H**CH$_{2,min}$), 6.79 (t, J = 5.7, 0.6H, N**H**CH$_{2,maj}$), 5.16–5.03 (m, 2H, OC**H**$_2$Ph), 4.94–4.80 (m, 1H, C$^\beta$H), 4.57–4.50 (m, 1H, C$^\alpha$H), 3.70–3.58 (m, 2H, C**H**$_2$C(CH$_3$)$_3$), 3.26–3.11 (m, 2H, NHC**H**$_2$), 1.47–1.37 (m, 2H, NHCH$_2$C**H**$_2$), 1.32–1.23 (m, 5H, C**H**$_2$CH$_3$ + C$^\gamma$H$_3$), 1.20 (s, 3.6H, C(CH$_3$)$_{3,min}$), 1.18 (s, 5.4H, C(CH$_3$)$_{3,maj}$), 0.89–0.83 (m, 12H, CH$_2$C**H**$_3$ + CH$_2$C(C**H**$_3$)$_3$)

^{13}C NMR (CDCl$_3$, 101 MHz): δ_C 179.0 (m, C=O), 168.5 (C=O$_{maj}$), 168.3 (C=O$_{min}$), 135.9–135.8 (m, Ar), 128.7–128.6 (m, Ar), 128.1 (Ar), 127.9 (Ar), 77.5 (m, **C**H$_2$C(CH$_3$)$_3$), 74.3–74.1 (m, C$^\beta$H), 69.8–69.5 (m, O**C**H$_2$Ph), 56.9–56.7 (m, C$^\alpha$H), 39.3 (NHCH$_2$), 38.9 (**C**(CH$_3$)$_3$), 32.2–32.0 (m, CH$_2$**C**(CH$_3$)$_3$), 31.5 (NHCH$_2$**C**H$_2$), 27.5 (m, C(**C**H$_3$)$_3$), 26.0 (m, CH$_2$C(**C**H$_3$)$_3$), 20.0 (**C**H$_2$CH$_3$), 18.0–17.7 (C$^\gamma$H$_3$), 13.7 (CH$_2$**C**H$_3$)

FTIR (thin film): 3308 (br), 2959, 2933, 2872, 1645, 1258

HRMS (ESI$^+$): Calc. for $C_{25}H_{43}N_2NaO_6P$ [M+Na]$^+$ 521.2751, found 521.2742

Ac-L-Thr(PO$_2$H(OCH$_2$*t*Bu))-NHBu **2e**

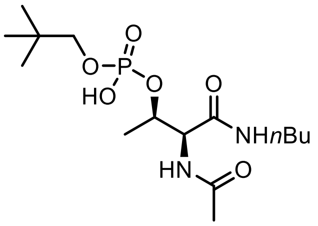

Prepared according to General Procedure D, using **17** (70 mg, 0.15 mmol) in MeOH (2 mL). The title compound (55 mg, 98%) was obtained as a colourless solid.

^{1}H NMR (CDCl$_3$, 400 MHz): δ_H 8.28 (br s, 1H, OH), 7.42–7.27 (m, 2H, 2x NH), 4.85–4.72 (m, 1H, C$^\beta$H), 4.70–4.60 (m, 1H, C$^\alpha$H), 3.73–3.58 (m, 2H, OCH$_2$), 3.34–3.12 (m, 2H, NHC**H**$_2$), 2.06 (s, 3H, CH$_3^{Ac}$), 1.52–1.44 (m, 2H, NHCH$_2$C**H**$_2$), 1.39– 1.25 (m, 5H, C**H**$_2$CH$_3$ + C$^\gamma$H$_3$), 0.94–0.85 (m, 12H, CH$_2$C**H**$_3$ + C(CH$_3$)$_3$)

^{13}C NMR (CDCl$_3$, 101 MHz): δ_C 171.3 (C=O), 168.7 (C=O), 77.1 (partially obscured by solvent peak, OCH$_2$), 73.3 (C$^\beta$H), 57.2 (C$^\alpha$H), 39.7 (NH**C**H$_2$), 32.2 (**C**(CH$_3$)$_3$), 31.4 (NHCH$_2$**C**H$_2$), 26.1 (C(**C**H$_3$)$_3$), 23.0 (CH$_3^{Ac}$), 20.1 (**C**H$_2$CH$_3$), 17.6 (C$^\gamma$H$_3$), 13.8 (CH$_2$**C**H$_3$)

FTIR (thin film): 3287 (br), 2959, 2936, 2873, 1646, 1537, 1003

HRMS (Nanospray$^-$): Calc. for $C_{15}H_{30}N_2O_6P$ [M-H]$^-$ 365.1841, found 365.1847

Piv-L-Thr(PO$_2$H(OCH$_2$*t*Bu))-NHBu **2f**

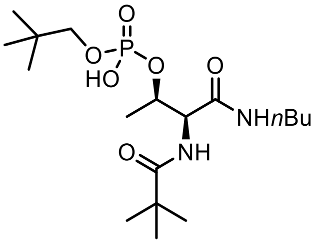

Prepared according to General Procedure D using **18** (82 mg, 0.16 mmol) in MeOH (2 mL). The title compound (65 mg, 97%) was obtained as a colourless semi-solid.

^{1}H NMR (400 MHz, CDCl$_3$): δ_H 9.10 (br s, 1H, OH), 7.25 (br, 1H, NH), 6.98 (d, *J* = 6.1, 1H, N$^\alpha$H), 4.84–4.76 (m, 1H, C$^\beta$H), 4.64–4.60 (m, 1H, C$^\alpha$H), 3.67–3.61 (m, 2H, OCH$_2$), 3.35–3.16 (m, 2H, NHC**H**$_2$), 1.52–1.45 (m, 2H, NHCH$_2$C**H**$_2$), 1.39–1.31 (m, 2H, C**H**$_2$CH$_3$), 1.24–1.19 (m, 12H, C(CH$_3$)$_3$ + C$^\gamma$H$_3$), 0.92–0.87 (m, 12H, C(CH$_3$)$_3$ + CH$_2$C**H**$_3$)

^{13}C NMR (101 MHz, CDCl$_3$): δ_C 179.2 (C=O), 168.3 (C=O), 77.0 (partially obscured by solvent peak, OCH$_2$), 72.7 (C$^\beta$H), 56.4 (C$^\alpha$H), 39.6 (NH$_2$**C**H$_2$), 38.9 (**C**(CH$_3$)$_3$), 32.1 (d, $^3J_{CP}$ = 7.5, **C**(CH$_3$)$_3$), 31.4 (NHCH$_2$**C**H$_2$), 27.5 (C(**C**H$_3$)$_3$), 26.1 (C(**C**H$_3$)$_3$), 20.1 (**C**H$_2$CH$_3$), 17.1 (C$^\gamma$H), 13.8 (CH$_2$**C**H$_3$)

FTIR (thin film): 3301 (br), 2960, 2937, 2873, 1644, 1511, 1010

HRMS (Nanospray$^-$): Calc. for $C_{18}H_{36}N_2O_6P$ [M-H]$^-$ 407.2311, found 407.2319

S2.5 Synthesis of carboxylate test ligand **3**

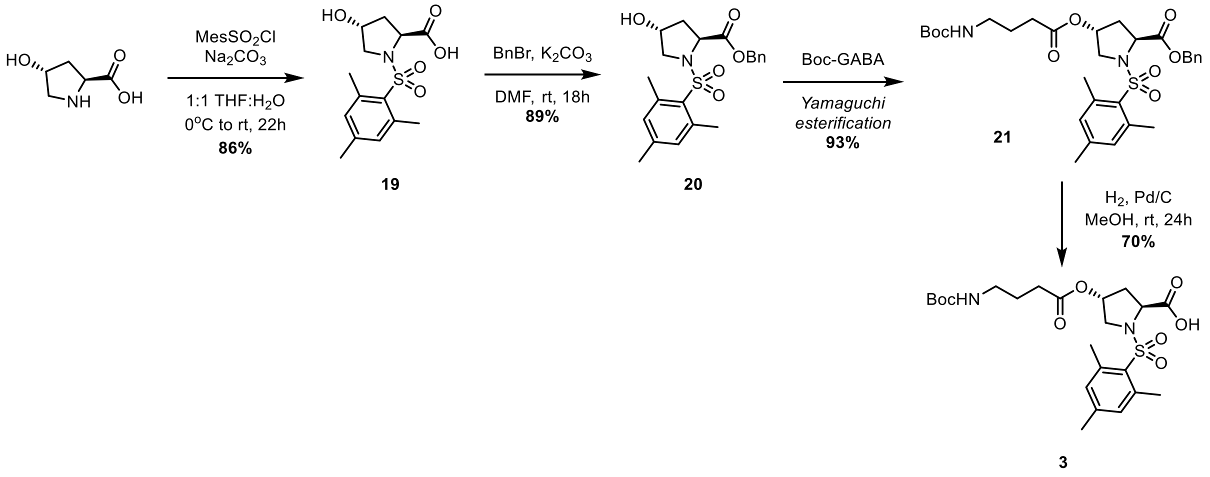

Scheme 4. Synthetic route to ligand **3**

<u>*N*-Mesitylsulfonyl-L-hydroxyproline (MesSO$_2$-L-Hyp-OH) **19**</u>

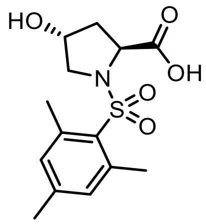

2-Mesitylenesulfonyl chloride (875 mg, 4.0 mmol) was added in portions to a solution of L-hydroxyproline (629 mg, 4.8 mmol) and sodium carbonate (1.02 g, 9.6 mmol) in 1:1 THF/H_2O (16 mL) at 0 °C. The biphasic mixture was warmed to room temperature and stirred for 22 hours. The THF was evaporated under reduced pressure, then the residue was acidified to pH 1-2 (conc. aq. HCl) and extracted with EtOAc. The combined organic extracts were washed with brine, then dried ($MgSO_4$), filtered and concentrated under reduced pressure to give the title compound (1.08 g, 86%) as a colourless solid.

^{1}H NMR (400 MHz, CDCl$_3$): δ_H 6.94 (s, 2H, ArH), 5.99 (br s, 2H, OH + CO_2H fast exchange), 4.57 (t, *J* = 7.9, 1H, C$^\alpha$H), 4.50–4.47 (m, 1H, C$^\gamma$H), 3.49–3.45 (m, 1H, C$^\delta$**H**H), 3.41 (dd, *J* = 11.8, 4.0, 1H, C$^\delta$H**H**), 2.63 (s, 6H, 2,6-CH$_3$), 2.45–2.38 (m, 1H, C$^\beta$**H**H), 2.28 (s, 3H, 4-CH$_3$), 2.21 (ddd, *J* = 13.4, 7.4, 4.9, 1H, C$^\beta$H**H**).

^{13}C NMR (101 MHz, CDCl$_3$): δ_C 176.2 (C=O), 143.7 (Ar), 141.0 (Ar), 132.2 (Ar), 131.4 (Ar), 70.5 (C$^\gamma$H), 58.2 (C$^\alpha$H), 55.8 (C$^\delta$H$_2$), 40.1 (C$^\beta$H$_2$), 23.0 (2,6-CH$_3$), 21.2 (4-CH$_3$)

FTIR (thin film): 3460 (br), 2983, 2942, 1726, 1313, 1149

HRMS (Nanospray$^-$): Calc. for $C_{14}H_{18}NO_5S$ [M-H]$^-$ 312.0906, found 312.0896

Benzyl *N*-mesitylsulfonyl-L-hydroxyprolinate ($MesSO_2$-L-Hyp-OBn) **20**

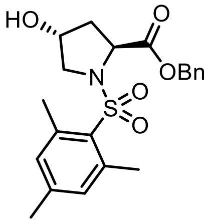

Prepared according to General Procedure A using **19** (1.08 g, 3.45 mmol). The crude product was purified by silica column chromatography (1:1 EtOAc:P.E., R_f = 0.33) to give the title compound (1.23 g, 89%) as a colourless oil.

^{1}H NMR (400 MHz, $CDCl_3$): δ_H 7.37–7.30 (m, 3H, ArH), 7.24–7.20 (m, 2H, ArH), 6.89 (s, 2H, ArH), 4.88 (d, *J* = 12.3, 1H, C**H**HPh), 4.82 (d, *J* = 12.3, 1H, CH**H**Ph), 4.60–4.54 (m, 1H, $C^{\alpha}H$), 4.50–4.44 (m, 1H, $C^{\gamma}H$), 3.68 (dt, *J* = 12.1, 1.9, 1H, C^{δ}**H**H), 3.46 (dd, *J* = 12.1, 3.8, 1H, C^{δ}H**H**), 2.70 (d, *J* = 6.4, 1H, OH), 2.61 (s, 6H, 2,6-CH_3), 2.39 (dddd, *J* = 13.2, 8.2, 2.6, 1.9, 1H, C^{β}**H**H), 2.27 (s, 3H, 4-CH_3), 2.16 (ddd, *J* = 13.2, 7.7, 4.8, 1H, C^{β}H**H**)

^{13}C NMR (101 MHz, $CDCl_3$): δ_C 171.6 (C=O), 143.3 (Ar), 141.0 (Ar), 135.2 (Ar), 132.0 (Ar), 131.7 (Ar), 128.6 (Ar), 128.5 (Ar), 128.2 (Ar), 70.5 ($C^{\gamma}H$), 67.05 (**C**H_2Ph), 58.4 ($C^{\alpha}H$), 56.2 ($C^{\delta}H_2$), 40.4 ($C^{\beta}H_2$), 22.9 (2,6-CH_3), 21.1 (4-CH_3)

FTIR (thin film): 3501 (br), 3033, 2978, 2941, 1738, 1315, 1148

HRMS (ESI$^+$): Calc. for $C_{21}H_{25}NNaO_5S$ $[M+Na]^+$ 426.1346, found 426.1355

Benzyl *O*^{3}-(γ-(tert-butoxycarbonyl)aminobutyryl)-*N*-mesitylsulfonyl-L-hydroxyprolinate **21**

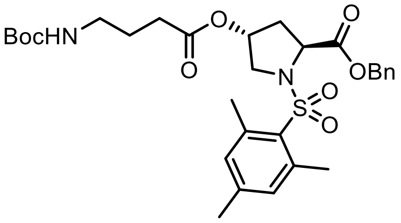

Prepared according to a modified literature procedure.[6]

2,4,6-Trichlorobenzoyl chloride (0.47 mL, 3.0 mmol) was added to a solution of *N*-Boc-GABA (610 mg, 3.0 mmol) and triethylamine (0.49 mL, 3.5 mmol) in THF (25 mL). The mixture was stirred at room temperature for 1 hour, then filtered and evaporated. The residue was dissolved in a solution of **20** (1.00 g, 2.5 mmol) and DMAP (305 mg, 2.5 mmol) in toluene (20 mL), and the mixture stirred at 70 °C for 1 hour. After cooling to room temperature, the mixture was diluted with Et_2O then washed with aq. HCl (1 M), sat. $NaHCO_3$ and brine. The organic layer was dried ($MgSO_4$), filtered and concentrated under reduced pressure to give the crude product, which was purified by silica column

chromatography (30:70 to 40:60 EtOAc:P.E., R_f = 0.28 at 40:60) to give the title compound (1.37 g, 93%) as a colourless oil.

^{1}H NMR (400 MHz, $CDCl_3$): δ_H 7.36–7.30 (m, 3H, ArH), 7.22–7.18 (m, 2H, ArH), 6.87 (s, 2H, ArH), 5.26–5.23 (m, 1H, Hyp-C$^\gamma$H), 4.85–4.77 (m, 3H, C**H**$_2$Ph + NH), 4.55 (t, *J* = 7.9, 1H, Hyp-C$^\alpha$H), 3.90 (dt, *J* = 12.4, 1.8, 1H, Hyp-C$^\delta$H), 3.57 (dd, *J* = 12.4, 3.9, 1H, Hyp-C$^\delta$H), 3.21–3.07 (m, 2H, GABA-C$^\gamma$H$_2$), 2.57 (s, 6H, 2,6-CH$_3$), 2.47 (ddt, *J* = 13.9, 8.0, 2.2, 1H, Hyp-C$^\beta$H), 2.35 (td, *J* = 7.2, 2.4, 2H, GABA-C$^\alpha$H$_2$), 2.31–2.24 (m, 4H, Hyp-C$^\beta$H + 4-CH$_3$), 1.80 (quint, *J* = 7.1, 2H, GABA-C$^\beta$H$_2$), 1.42 (s, 9H, C(CH$_3$)$_3$)

^{13}C NMR (101 MHz, $CDCl_3$): δ_C 172.8 (C**C**=O), 171.2 (C**C**=O), 156.1 (N**C**=O), 142.9 (Ar), 140.6 (Ar), 135.1 (Ar), 132.6 (Ar), 132.0 (Ar), 128.6 (Ar), 128.5 (Ar), 128.2 (Ar), 79.2 (**C**(CH$_3$)$_3$), 72.6 (Hyp-C$^\gamma$H), 67.2 (**C**H$_2$Ph), 58.8 (Hyp-C$^\alpha$H) , 53.7 (Hyp-C$^\delta$H$_2$), 39.8 (GABA-C$^\gamma$H$_2$), 37.3 (Hyp-C$^\beta$H$_2$), 31.5 (GABA-C$^\alpha$H$_2$), 28.5 (C(**C**H$_3$)$_3$), 25.1 (GABA-C$^\beta$H$_2$), 22.9 (2,6-CH$_3$), 21.1 (4-CH$_3$)

FTIR (thin film): 3404, 2977, 2939, 2887, 1736, 1708, 1152

HRMS (ESI$^+$): Calc. for $C_{30}H_{41}N_2O_8S$ [M+H]$^+$ 589.2578, found 589.2551

<u>*O*^{3}-(γ-(*tert*-Butoxycarbonyl)aminobutyryl)-*N*-mesitylsulfonyl-L-hydroxyproline **3**</u>

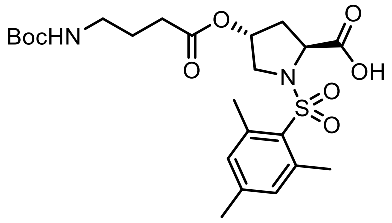

Prepared according to General Procedure D using **21** (118 mg, 0.2 mmol) in MeOH (2 mL). The crude product was redissolved in EtOAc and extracted with sat. $NaHCO_3$. The combined aqueous extracts were acidified to pH ~1 (conc. HCl) and re-extracted with EtOAc. The combined organic extracts were washed with brine, dried (Na_2SO_4), filtered and concentrated under reduced pressure to give the title compound (70 mg, 70%) as a colourless semi-solid.

^{1}H NMR (400 MHz, $CDCl_3$): δ_H 7.31 (br s, 1H, OH), 6.92 (s, 2H, ArH), 5.91 (br s, 0.3H, NH$_{min}$), 5.26–5.22 (m, 1H, Hyp-C$^\gamma$H), 4.84 (br s, 0.7H, NH$_{maj}$), 4.53 (t, *J* = 7.8, 1H, Hyp-C$^\alpha$H), 3.70 (d, *J* = 12.2, 1H, Hyp-C$^\delta$**H**H), 3.55 (dd, *J* = 12.2, 4.2, 1H, Hyp-C$^\delta$H**H**), 3.18–3.03 (m, 2H, GABA-C$^\gamma$H$_2$), 2.60 (s, 6H, 2,6-CH$_3$), 2.52–2.42 (m, 1H, Hyp-C$^\beta$**H**H), 2.36–2.29 (m, 3H, Hyp-C$^\beta$H**H** + GABA-C$^\alpha$H$_2$), 2.27 (s, 3H, 4-CH$_3$), 1.77 (quint, *J* = 7.0, 2H, GABA-C$^\beta$H$_2$), 1.42 (s, 9H, C(CH$_3$)$_3$)

^{13}C NMR (101 MHz, $CDCl_3$): δ_C 175.4 (C**C**=O), 172.9 (C**C**=O), 156.3 (N**C**=O), 143.2 (Ar), 140.7 (Ar), 132.3 (Ar), 132.0 (Ar), 79.5 (**C**(CH$_3$)$_3$), 72.7 (Hyp-C$^\gamma$H), 58.6 (Hyp-C$^\alpha$H), 53.4 (Hyp-C$^\gamma$H$_2$), 39.8 (GABA-C$^\gamma$H$_2$), 37.1 (Hyp-C$^\beta$H$_2$), 31.5 (GABA-C$^\alpha$H$_2$), 28.5 (C(**C**H$_3$)$_3$), 25.1 (GABA-C$^\beta$H$_2$), 23.0 (2,6-CH$_3$), 21.1 (4-CH$_3$)

FTIR (thin film): 3386 (br), 2977, 2939, 2877, 1735 (br), 1153

HRMS (Nanospray$^-$): Calc. for $C_{23}H_{33}N_2O_8S$ [M-H]$^-$ 497.1958, found 497.1962

S2.6 Synthesis of pseudopeptide ligand **4**

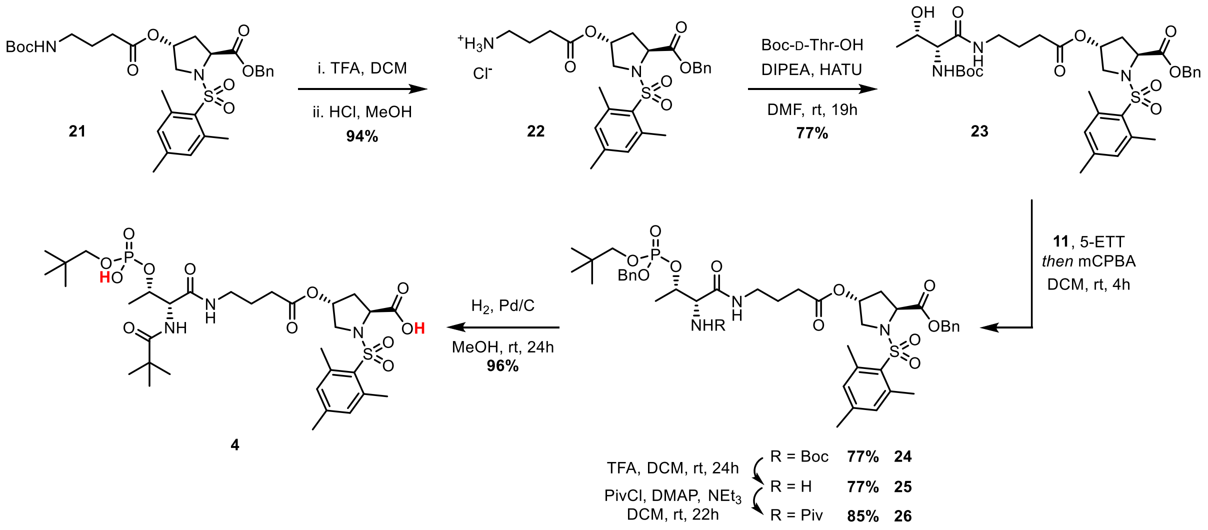

Scheme 5. Synthetic route to ligand **4**

Benzyl O^3-(γ-aminobutyryl)-*N*-mesitylsulfonyl-L-hydroxyprolinate hydrochloride **22**

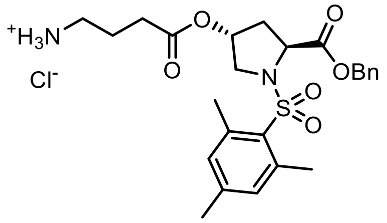

Trifluoroacetic acid (0.39 mL, 5.0 mmol) was added to a solution of **21** (293 mg, 0.5 mmol) in DCM (2.5 mL). The mixture was stirred at room temperature for 20 hours, then the volatiles were evaporated under reduced pressure. The residue was redissolved in methanolic HCl (1.5 mL, 1.25 M) and stirred for 30 mins. The volatiles were evaporated under reduced pressure and the residue triturated with Et_2O to give the title compound (244 mg, 94%) as a colourless hygroscopic solid, which was stored in a vacuum desiccator over calcium chloride.

^{1}H NMR ($CDCl_3$, 400 MHz): δ_H 8.15 (br s, 3H, NH_3^+), 7.35–7.27 (m, 3H, ArH), 7.20–7.16 (m, 2H, ArH), 6.84 (s, 2H, ArH), 5.27–5.21 (m, 1H, Hyp-C$^\gamma$H), 4.81–4.75 (m, 2H, C**H**$_2$Ph), 4.51 (t, *J* = 7.6, 1H, Hyp-C$^\alpha$H), 3.84 (d, *J* = 12.1, 1H, Hyp-C$^\delta$**H**H), 3.58–3.51 (m, 1H, Hyp-C$^\delta$H**H**), 3.16–3.06 (m, 2H, GABA-C$^\gamma$H$_2$), 2.54–2.43 (m, 9H, 2,6-CH$_3$ + GABA-C$^\alpha$H$_2$ + Hyp-C$^\beta$**H**H), 2.28–2.21 (m, 4H, 4-CH$_3$ + Hyp-C$^\beta$H**H**), 2.13–2.05 (m, 2H, GABA-C$^\beta$H$_2$)

^{13}C NMR ($CDCl_3$, 101 MHz): δ_C 172.2 (C=O), 171.2 (C=O), 143.0 (Ar), 140.6 (Ar), 135.1 (Ar), 132.5 (Ar), 132.0 (Ar), 128.6 (Ar), 128.5 (Ar), 128.1 (Ar), 72.9 (Hyp-C$^\gamma$H), 67.1 (**C**H$_2$Ph), 58.7 (Hyp-C$^\alpha$H), 53.6 (Hyp-C$^\delta$H$_2$), 39.3 (GABA-C$^\gamma$H$_2$), 37.2 (Hyp-C$^\beta$H$_2$), 31.1 (GABA-C$^\alpha$H$_2$), 22.9 (2,6-CH$_3$), 22.5 (GABA-C$^\beta$H$_2$), 21.1 (4-CH$_3$)

FTIR (thin film): 3403 (br), 3028, 2978, 2940, 1733, 1317, 1173, 1151

HRMS (ESI$^+$): Calc. for $C_{25}H_{33}N_2O_6S$ $[M]^+$ 489.2054, found 489.2062

Benzyl (2S,4R)-4-((4-((2R,3S)-2-((tert-butoxycarbonyl)amino)-3-hydroxybutanamido)butanoyl)oxy)-1-(mesitylsulfonyl)pyrrolidine-2-carboxylate **23**

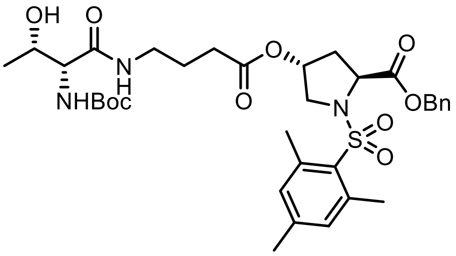

Boc-D-Thr-OH (271 mg, 1.24 mmol) and **22** (684 mg, 1.30 mmol) was dissolved in DMF (4 mL). HATU (470 mg, 1.24 mmol) was added dropwise as a solution in DMF (2 mL), followed by the dropwise addition of DIPEA (0.65 mL, 3.7 mmol). The mixture was stirred at room temperature for 19 h, then diluted with water (100 mL) and extracted with DCM (4 x 15 mL). The combined organic extracts were washed with aq. HCl (1 M), sat. $NaHCO_3$ and brine, then dried ($MgSO_4$), filtered and concentrated under reduced pressure. The crude product was purified by silica column chromatography (65:35 EtOAc:P.E., R_f = 0.27) to give the title compound (655 mg, 77%) as a colourless semi-solid.

^{1}H NMR (400 MHz, $CDCl_3$): δ_H 7.37–7.30 (m, 3H, ArH), 7.22–7.17 (m, 2H, ArH), 6.87 (s, 2H, ArH), 6.82–6.77 (m, 1H, NH), 5.49 (d, J = 8.4, 1H, NH), 5.27–5.23 (m, 1H, Hyp-C$^\gamma$H), 4.80 (d, J = 12.3, 1H, C**H**HPh), 4.75 (d, J = 12.3, 1H, CH**H**Ph), 4.56 (td, J = 7.9, 0.6, 1H, Hyp-C$^\alpha$H), 4.39–4.33 (m, 1H, Thr-C$^\beta$H), 4.03–3.99 (m, 1H, Thr-C$^\alpha$H), 3.98–3.93 (m, 1H, Hyp-C$^\delta$**H**H), 3.56 (dd, J = 12.4, 3.8, 1H, Hyp-C$^\delta$H**H**), 3.44–3.34 (m, 2H, GABA-C$^\gamma$**H**H + OH), 3.26–3.18 (m, 1H, GABA-C$^\gamma$H**H**), 2.57 (s, 6H, 2,6-CH_3), 2.50 (ddt, J = 13.8, 7.9, 2.1, 1H, Hyp-C$^\beta$**H**H), 2.37 (td, J = 7.1, 3.7, 2H, GABA-C$^\alpha H_2$), 2.31–2.24 (m, 4H, 4-CH_3 + Hyp-C$^\beta$H**H**), 1.92–1.75 (m, 2H, GABA-C$^\beta H_2$), 1.43 (s, 9H, $C(CH_3)_3$), 1.16 (d, J = 6.4, 3H, Thr-C$^\gamma H_3$)

^{13}C NMR (101 MHz, $CDCl_3$): δ_C 172.7 (C=O), 171.9 (C=O), 171.2 (C=O), 156.6 (C=O), 143.0 (Ar), 140.6 (Ar), 135.1 (Ar), 132.7 (Ar), 132.0 (Ar), 128.64 (Ar), 128.55 (Ar), 128.2 (Ar), 80.4 (**C**($CH_3)_3$), 72.7 (Hyp-C$^\gamma$H), 67.2 (**C**H_2Ph), 67.0 (Thr-C$^\beta$H), 58.8 (Hyp-C$^\alpha$H), 58.3 (Thr-C$^\alpha$H), 54.0 (Hyp-C$^\delta H_2$), 38.5 (GABA-C$^\gamma H_2$), 37.4 (Hyp-C$^\beta H_2$), 31.3 (GABA-C$^\alpha H_2$), 28.4 (C(**C**$H_3)_3$), 24.5 (GABA-C$^\beta H_2$), 22.9 (2,6-CH_3), 21.1 (4-CH_3), 18.7 (Thr-C$^\gamma H_3$)

FTIR (thin film): 3367 (br), 2977, 2938, 1736, 1659, 1155

HRMS (ESI$^+$): Calc. for $C_{34}H_{47}N_3NaO_{10}S$ $[M+Na]^+$ 712.2874, found 712.2889

Benzyl (2*S*,4*R*)-4-((4-((2*R*,3*S*)-3-(((benzyloxy)(neopentyloxy)phosphoryl)oxy)-2-((tert-butoxycarbonyl) amino)butanamido)butanoyl)oxy)-1-(mesitylsulfonyl)pyrrolidine-2-carboxylate **24**

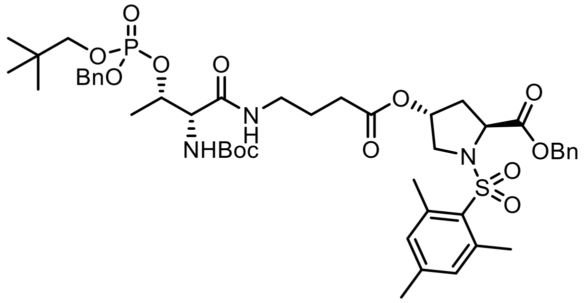

Prepared according to General Procedure C using alcohol **23** (613 mg, 0.89 mmol) and phosphoramidite **11** (356 mg, 1.09 mmol). The crude product was purified by silica column chromatography (30% to 100% EtOAc in P.E., R_f = 0.32 at 60% EtOAc) to give the title compound (635 mg, 77%) as a colourless oil. Isolated as a 45:55 mixture of diastereomers.

^{1}H NMR (CDCl$_3$, 400 MHz): δ_H 7.42–7.30 (m, 8H, ArH), 7.23–7.18 (m, 2H, ArH), 6.87 (s, 2H, ArH), 6.78–6.74 (m, 0.55H, NH_{maj}), 6.70–6.66 (m, 0.45H, NH_{min}), 5.55–5.50 (m, 1H, NH), 5.25–5.21 (m, 1H, Hyp-C^{γ}H), 5.11–5.02 (m, 2H, POC**H**$_2$Ph), 4.99–4.91 (m, 1H, Thr-C^{β}H), 4.85–4.76 (m, 2H, COC**H**$_2$Ph), 4.55 (t, *J* = 7.8, 1H, Hyp-C^{α}H), 4.29–4.23 (m, 1H, Thr-C^{α}H), 3.92–3.87 (m, 1H, Hyp-C^{δ}**H**H), 3.71–3.60 (m, 2H, C**H**$_2$C(CH$_3$)$_3$), 3.59–3.54 (m, 1H, Hyp-C^{δ}H**H**), 3.30–3.22 (m, 2H, GABA-$C^{\gamma}H_2$), 2.57 (s, 6H, 2,6-CH$_3$), 2.48–2.42 (m, 1H, Hyp-C^{β}**H**H), 2.41–2.23 (m, 6H, 4-CH$_3$ + GABA-$C^{\alpha}H_2$ + Hyp-C^{β}H**H**), 1.88–1.76 (m, 2H, GABA-$C^{\beta}H_2$), 1.42 (s, 4.95H, $C(CH_3)_{3,maj}$), 1.40 (s, 4.05H, $C(CH_3)_{3,min}$), 1.34–1.31 (m, 3H, Thr-$C^{\gamma}H_3$), 0.90 (s, 4.05H, $C(CH_3)_{3,min}$), 0.90 (s, 4.95H, $C(CH_3)_{3,maj}$)

^{13}C NMR (CDCl$_3$, 101 MHz): δ_C 172.7 (C=O), 171.3 (C=O), 168.9 (C=O), 155.75 (C=O), 142.9 (Ar), 140.6 (Ar), 136.1 (d, $^3J_{CP}$ = 7.0, Ar), 135.1 (Ar), 132.7 (Ar), 132.0 (Ar), 128.7 (Ar), 128.6 (Ar), 128.5 (Ar), 128.22 (Ar), 128.15 (Ar), 128.0 (Ar), 80.4 (O**C**(CH$_3$)$_3$), 77.4 (partially obscured by solvent peak, **C**H$_2$C(CH$_3$)$_3$), 74.9 (d, $^2J_{CP}$ = 6.2, Thr-C^{β}H), 72.7 (Hyp-C^{γ}H), 69.6–69.5 (m, PO**C**H$_2$Ph), 67.2 (CO**C**H$_2$Ph), 58.8 (Hyp-C^{α}H), 58.6–58.5 (m, Thr-C^{α}H), 53.8 (m, Hyp-$C^{\delta}H_2$), 38.9 (GABA-$C^{\gamma}H_2$), 37.3 (Hyp-$C^{\beta}H_2$), 32.2 (d, $^3J_{CP}$ = 7.7, CH$_2$**C**(CH$_3$)$_3$), 31.4 (m, GABA-$C^{\alpha}H_2$), 28.4 (OC(**C**H$_3$)$_3$), 26.1 (CH$_2$C(**C**H$_3$)$_3$), 24.5–24.4 (m, GABA-$C^{\beta}H_2$), 22.9 2,6-CH$_3$), 21.1 (4-CH$_3$), 18.0–17.8 (m, Thr-$C^{\gamma}H_3$)

FTIR (thin film): 3313, 2962, 2938, 1736, 1717, 1675, 1155, 1007

HRMS (ESI$^+$): Calc. for $C_{46}H_{64}N_3O_{13}PS$ [M+H]$^+$ 930.3970, found 930.3986

Benzyl (2*S*,4*R*)-4-((4-((2*R*,3*S*)-2-amino-3-(((benzyloxy)(neopentyloxy)phosphoryl)oxy)butanamido) butanoyl)oxy)-1-(mesitylsulfonyl)pyrrolidine-2-carboxylate **25**

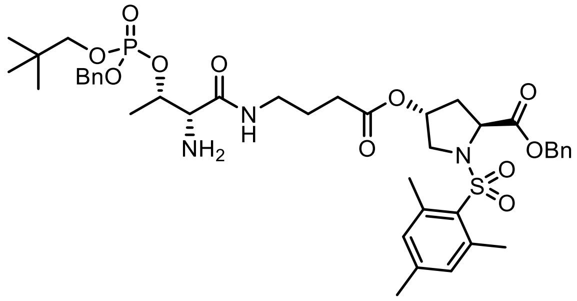

Trifluoroacetic acid (0.39 mL, 5.1 mmol) was added to a solution of **24** (473 mg, 0.51 mmol) in DCM (5 mL). The mixture was stirred at room temperature for 24 hours, then the volatiles were evaporated under reduced pressure. The residue was redissolved in EtOAc and washed with sat. $NaHCO_3$ (aq.) and brine, then dried (Na_2SO_4), filtered and concentrated under reduced pressure to give the crude free amine, which was purified by silica column chromatography (0 to 6% MeOH in EtOAc, R_f = 0.32 at 6% MeOH) to give the title compound (323 mg, 77%) as a colourless oil. Isolated as a 45:55 mixture of diastereomers.

^{1}H NMR ($CDCl_3$, 400 MHz): δ_H 7.50 (t, *J* = 6.1, 0.55H, NH_{maj}), 7.45 (t, *J* = 6.0, 0.45H, NH_{min}), 7.39–7.27 (m, 8H, ArH), 7.20–7.16 (m, 2H, ArH), 6.85 (s, 2H, ArH), 5.24–5.19 (m, 1H, Hyp-C^{γ}H), 5.09–4.99 (m, 2H, POC**H**$_2$Ph), 4.98–4.91 (m, 1H, Thr-C^{β}H), 4.81 (d, *J* = 12.3, 1H, COC**H**HPh), 4.76 (d, *J* = 12.3, 1H, COCH**H**Ph), 4.52 (t, *J* = 7.8, 1H, Hyp-C^{α}H), 3.87 (dt, *J* = 12.2, 1.8, 1H, Hyp-C^{δ}**H**H), 3.66–3.53 (m, 3H, Hyp-C^{δ}H**H** + C**H**$_2$C(CH_3)$_3$), 3.33–3.14 (m, 3H, Thr-C^{α}H + GABA-$C^{\gamma}H_2$), 2.55 (s, 6H, 2,6-CH_3), 2.47–2.39 (m, 1H, Hyp-C^{β}**H**H), 2.35–2.22 (m, 6H, 4-CH_3 + GABA-$C^{\alpha}H_2$ + Hyp-C^{β}H**H**), 1.84–1.70 (m, 4H, GABA-$C^{\beta}H_2$ + NH_2), 1.39–1.36 (m, 3H, Thr-$C^{\gamma}H_3$), 0.88 (s, 9H, C(CH_3)$_3$)

^{13}C NMR ($CDCl_3$, 101 MHz): δ_C 172.5 (C=O), 171.6 (C=O), 171.1 (C=O), 142.8 (Ar), 140.5 (Ar), 136.12 (d, $^3J_{CP}$ = 6.8, Ar), 135.0 (Ar), 132.6 (Ar), 131.9 (Ar), 128.6 (Ar), 128.5 (Ar), 128.4 (Ar), 128.1 (Ar), 127.9 (Ar), 127.8 (Ar), 77.1–77.0 (m, **C**H_2C(CH_3)$_3$), 76.3–76.2 (Thr-C^{β}H), 72.5 (Hyp-C^{γ}H), 69.2–69.1 (PO**C**H_2Ph), 67.1 (CO**C**H_2Ph), 59.5 (d, $^3J_{CP}$ = 7.5, Thr-C^{α}H), 58.7 (Hyp-C^{α}H), 53.6 (Hyp-$C^{\delta}H_2$), 38.4 (m, GABA-$C^{\gamma}H_2$), 37.2 (Hyp-$C^{\beta}H_2$), 32.1 (d, $^2J_{CP}$ = 7.9, **C**(CH_3)$_3$), 31.5 (GABA-$C^{\alpha}H_{2,maj}$), 31.4 (GABA-$C^{\alpha}H_{2,min}$), 26.0 (C(**C**H_3)$_3$), 24.6 (m, GABA-$C^{\beta}H_2$), 22.8 (2,6-CH_3), 21.0 (4-CH_3), 18.5 (m, Thr-$C^{\gamma}H_3$)

FTIR (thin film): 3382 (br), 3325 (br), 2955, 2877, 1736, 1667, 1153, 1004

HRMS (ESI$^+$): Calc. for $C_{41}H_{57}N_3O_{11}PS$ $[M+H]^+$ 830.3446, found 830.3439

Benzyl (2*S*,4*R*)-4-((4-((2*R*,3*S*)-3-(((benzyloxy)(neopentyloxy)phosphoryl)oxy)-2-pivalamidobutanamido)butanoyl)oxy)-1-(mesitylsulfonyl)pyrrolidine-2-carboxylate **26**

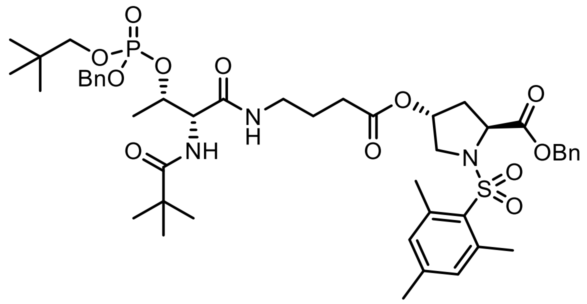

Pivaloyl chloride (43 µL, 0.35 mmol) was added to a solution of **25** (241 mg, 0.29 mmol), triethylamine (81 µL, 0.58 mmol) and DMAP (7 mg, 0.06 mmol) in DCM (3 mL) at room temperature. The mixture was stirred at room temperature for 22 hours, then diluted with EtOAc, washed with aq. HCl (1 M), sat. $NaHCO_3$ and brine, then dried (Na_2SO_4), filtered and concentrated under reduced pressure. The crude product was purified by silica column chromatography (60:40 EtOAc:P.E., R_f = 0.21) to give the title compound (225 mg, 85%) as a colourless semi-solid. Isolated as a 45:55 mixture of diastereomers.

^{1}H NMR ($CDCl_3$, 400 MHz): δ_H 7.41–7.28 (m, 8H, ArH), 7.21–7.17 (m, 2H, ArH), 7.01 (d, *J* = 7.1, 0.55H, NH_{maj}), 6.99–6.94 (m, 1H, NH_{min} + NH_{maj}), 6.89–6.84 (m, 2.45H, NH_{min} + ArH), 5.25–5.20 (m, 1H, Hyp-$C^{\gamma}H$), 5.13–5.03 (m, 2H, POC**H**$_2$Ph), 4.93–4.85 (m, 1H, Thr-$C^{\beta}H$), 4.83–4.75 (m, 2H, COC**H**$_2$Ph), 4.55–4.49 (m, 2H, Thr-$C^{\alpha}H$ + Hyp-$C^{\alpha}H$), 3.89–3.83 (m, 1H, Hyp-C^{δ}**H**H), 3.71–3.54 (m, 3H, Hyp-C^{δ}H**H** + C**H**$_2C(CH_3)_3$), 3.30–3.18 (m, 2H, GABA-$C^{\gamma}H_2$), 2.55 (s, 6H, 2,6-CH_3), 2.47–2.39 (m, 1H, Hyp-C^{β}**H**H), 2.37–2.22 (m, 6H, 4-CH_3 + GABA-$C^{\alpha}H_2$ + Hyp-C^{β}H**H**), 1.84–1.73 (m, 2H, GABA-$C^{\beta}H_2$), 1.31 (d, *J* = 6.4, 1H, Thr-$C^{\gamma}H_{3,min}$), 1.28 (d, *J* = 6.4, 1.65H, Thr-$C^{\gamma}H_{3,maj}$), 1.21 (s, 4.95H, $C(CH_3)_{3,maj}$), 1.19 (s, 4.05H, $C(CH_3)_{3,min}$), 0.89 (s, 4.05H, $C(CH_3)_{3,min}$), 0.86 (s, 4.95H, $C(CH_3)_{3,maj}$)

^{13}C NMR ($CDCl_3$, 101 MHz): δ_C 179.1 (C=O), 172.6 (C=O), 171.1 (C=O), 168.8 (C=O_{min}), 168.7 (C=O_{maj}), 142.8 (Ar), 140.5 (Ar), 135.9–135.8 (m, Ar), 135.0 (Ar), 132.6 (Ar), 131.9 (Ar), 128.6 (m, Ar), 128.6 (Ar), 128.4 (Ar), 128.1 (Ar), 128.1 (Ar), 127.9 (Ar), 77.5–77.4 (m, partially obscured by solvent peak, **C**$H_2C(CH_3)_3$), 74.3–74.1 (m, Thr-$C^{\beta}H$), 72.6 (m, Hyp-$C^{\gamma}H$), 69.8–69.5 (m, PO**C**H_2Ph), 67.1 (CO**C**H_2Ph), 58.7 (Hyp-$C^{\alpha}H$), 57.1–57.0 (m, Thr-$C^{\alpha}H$), 53.6 (m, Hyp-$C^{\delta}H_2$), 38.9 (**C**$(CH_3)_3$), 38.7 (GABA-$C^{\gamma}H_2$), 37.2 (Hyp-$C^{\beta}H_2$), 32.2–32.0 (CH_2**C**$(CH_3)_3$), 31.3 (GABA-$C^{\alpha}H_2$), 27.5 (m, C(**C**$H_3)_3$), 26.0 (m, C(**C**$H_3)_3$), 24.4 (m, GABA-$C^{\beta}H_2$), 22.8 (2,6-CH_3), 21.0 (4-CH_3), 18.1–17.9 (m, Thr-$C^{\gamma}H_3$)

FTIR (thin film): 3308 (br), 2961, 2870, 1737, 1654, 1154, 1003

HRMS (ESI$^+$): Calc. for $C_{46}H_{65}N_3O_{12}PS$ $[M+H]^+$ 914.4021, found 914.4017

(2*S*,4*R*)-4-((4-((2*R*,3*S*)-3-((Hydroxy(neopentyloxy)phosphoryl)oxy)-2-pivalamidobutanamido) butanoyl)oxy)-1-(mesitylsulfonyl)pyrrolidine-2-carboxylic acid **4**

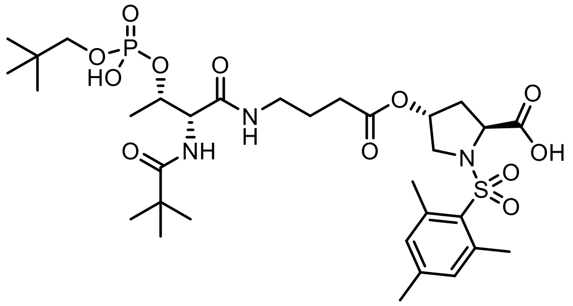

Prepared according to General Procedure D using **26** (112 mg, 0.12 mmol) in methanol (2 mL). The title compound (86 mg, 96%) was obtained as a colourless solid.

The ^{1}H NMR spectrum of **4** unexpectedly showed an additional minor set of peaks for some proton resonances, such as the aromatic mesitylene protons. These were unlikely to be due to rotamerism as these peaks resisted coalescence despite heating to 100 °C in DMSO-d_6. We speculate that the sample of **4** was contaminated by a small amount of its metal salts, formed during filtration of the reaction mixture through celite. Metal salt formation of highly acidic compounds is a well-documented problem.[7–9] These metals salts did not impede the operation of the reported automaton, so the conclusions drawn in the main article remain valid.

^{1}H NMR (CD_3OD, 400 MHz): δ_H 7.03–7.00 (m, 2H, ArH), 5.26–5.22 (m, 1H, Hyp-C$^\gamma$H), 4.76–4.68 (m, 1H, Thr-C$^\beta$H), 4.46–4.40 (m, 2H, Thr-C$^\alpha$H + Hyp-C$^\alpha$H), 3.80–3.68 (m, 1H, Hyp-C$^\delta$**H**H), 3.66 (d, $^3J_{PH}$ = 5.1, 2H, C**H**$_2$C(CH_3)$_3$), 3.62–3.56 (m, 1H, Hyp-C$^\delta$H**H**), 3.27–3.23 (m, 2H, GABA-C$^\gamma$H$_2$), 2.62–2.60 (m, 6H, 2,6-CH_3), 2.54–2.45 (m, 1H, Hyp-C$^\beta$**H**H), 2.41–2.27 (m, 6H, Hyp-C$^\beta$H**H** + GABA-C$^\alpha$H$_2$ + 4-CH_3), 1.85–1.76 (m, 2H, GABA-C$^\beta$H$_2$), 1.36 (d, *J* = 6.3, 3H, Thr-C$^\gamma$H$_3$), 1.24 (s, 9H, C(O)C(CH_3)$_3$), 0.96 (s, 9H, CH_2C(C**H**$_3$)$_3$))

^{13}C NMR (CD_3OD, 101 MHz): δ_C 181.2 (C=O), 174.7 (C=O), 174.1 (C=O), 171.2 (C=O), 144.5 (Ar), 141.7 (Ar), 133.9 (Ar), 133.0 (Ar), 77.9 (d, $^2J_{CP}$ = 6.5, **C**H$_2$C(CH_3)$_3$), 75.4 (d, $^2J_{CP}$ = 5.2, Thr-C$^\beta$H), 74.3 (Hyp-C$^\gamma$H), 60.2 (Hyp-C$^\alpha$H), 59.5 (d, $^3J_{CP}$ = 6.5, Thr-C$^\alpha$H), 54.3 (Hyp-C$^\delta$H$_2$), 39.9 (C(O)**C**(CH_3)$_3$), 39.8 (GABA-C$^\gamma$H$_2$), 38.1 (Hyp-C$^\beta$H$_2$), 32.9 (d, $^3J_{CP}$ = 8.2, CH_2**C**(CH_3)$_3$), 32.2 (GABA-C$^\alpha$H$_2$), 27.8 (C(O)C(**C**H$_3$)$_3$), 26.4 (CH_2C(**C**H$_3$)$_3$), 25.4 (GABA-C$^\beta$H$_2$), 23.2 (2,6-CH_3), 21.0 (4-CH_3), 19.0 (Thr-C$^\gamma$H$_3$)

FTIR (thin film): 3338, 2958, 2872, 1737, 1645, 1155, 1021

HRMS (Nanospray$^-$): Calc. for $C_{32}H_{51}N_3O_{12}PS$ [M-H]$^-$ 732.2931, found 732.2917

S3 CD spectroscopy methodology

S3.1 Stock solution preparation

Stock solutions of the receptor (Zn(BQPA)·2ClO_4) and ligands (**2a-c**, **3**, **4**) were prepared as 2.50 mM solutions by weighing out the appropriate mass of compound in a 5 mL volumetric flask, then dissolving in anhydrous MeCN. For volatile or hygroscopic compounds (NEt_3, *t*BuP_1(NMe_2)$_3$ and TCAH), an arbitrary amount (40-60 mg) of compound was weighed out in a pre-weighed, stoppered 5 mL volumetric flask (to minimise compound evaporation and water absorption) and dissolved in MeCN (for NEt_3 and *t*BuP_1(NMe_2)$_3$) or 20 vol% H_2O/MeCN (for TCAH) to give a solution of arbitrary, but known, concentration. An aliquot of this solution was then diluted to the desired concentration by the addition of an appropriate volume of solvent, as required.

S3.2 Base titrations of ligands

An initial sample (c_o = 0.25 mM) was prepared in a 1 mm path length quartz cuvette from Zn(BQPA).2ClO_4 (20 µL, 2.50 mM, 50 nmol) and ligand (20 µL, 2.50 mM, 50 nmol), made up to 200 µL with MeCN, and its CD spectrum (200–270 nm) was recorded. Then, NEt_3 (2.50 mM) was added in 10 µL (25 nmol, 0.5 eq.) increments and the sample gently shaken for homogeneity; the CD spectrum was re-recorded after each addition.

During such titration experiments, the sample was diluted after each addition of titrant. It was therefore necessary to convert the raw ellipticity output, θ, into molar values to account for this concentration change. The *molar ellipticity*, $[\theta]_\lambda$, was calculated using the following formula:

$$[\theta]_\lambda = \frac{\theta_\lambda}{cl}$$

Where θ_λ is the ellipticity measured at wavelength λ, c is the concentration of Zn(BQPA) in the sample and l is the path length of the cuvette. In practice, it is more convenient to rewrite c in terms of Zn(BQPA) stock solution concentration:

$$[\theta]_\lambda = \frac{V\theta_\lambda}{vc'l}$$

Where c' is the concentration of the Zn(BQPA) stock solution, V is the total volume of the sample and v is the volume of Zn(BQPA) stock solution used in the sample.

The complete base titration CD spectra for ligands **2a-f** are shown in Figs. **S1** and **S2**. Ligands **2a-c** possess both phosphate and carboxylate binding sites. The initial signal arises from deprotonation and binding of the more acidic phosphate site (Fig. **S1**). Further addition of base leads to deprotonation of the carboxylate and displacement of the phosphate site from the $[Zn(BQPA)]^{2+}$ receptor. The relative configuration of the threonine scaffold was chosen such that the phosphate and carboxylate sites induced CD signals of opposite sign upon binding to $[Zn(BQPA)]^{2+}$. Ligands **2d-f** were derivatised as threoninyl amides with only a phosphate binding site, so these gave just a single positive CD output (Fig. **S2**).

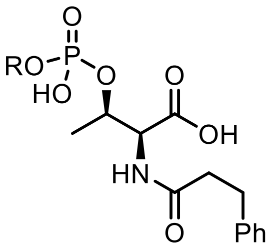

R = Ph **2a**
= $(CH_2)_2Ph$ **2b**
= CH_2*t*Bu **2c**

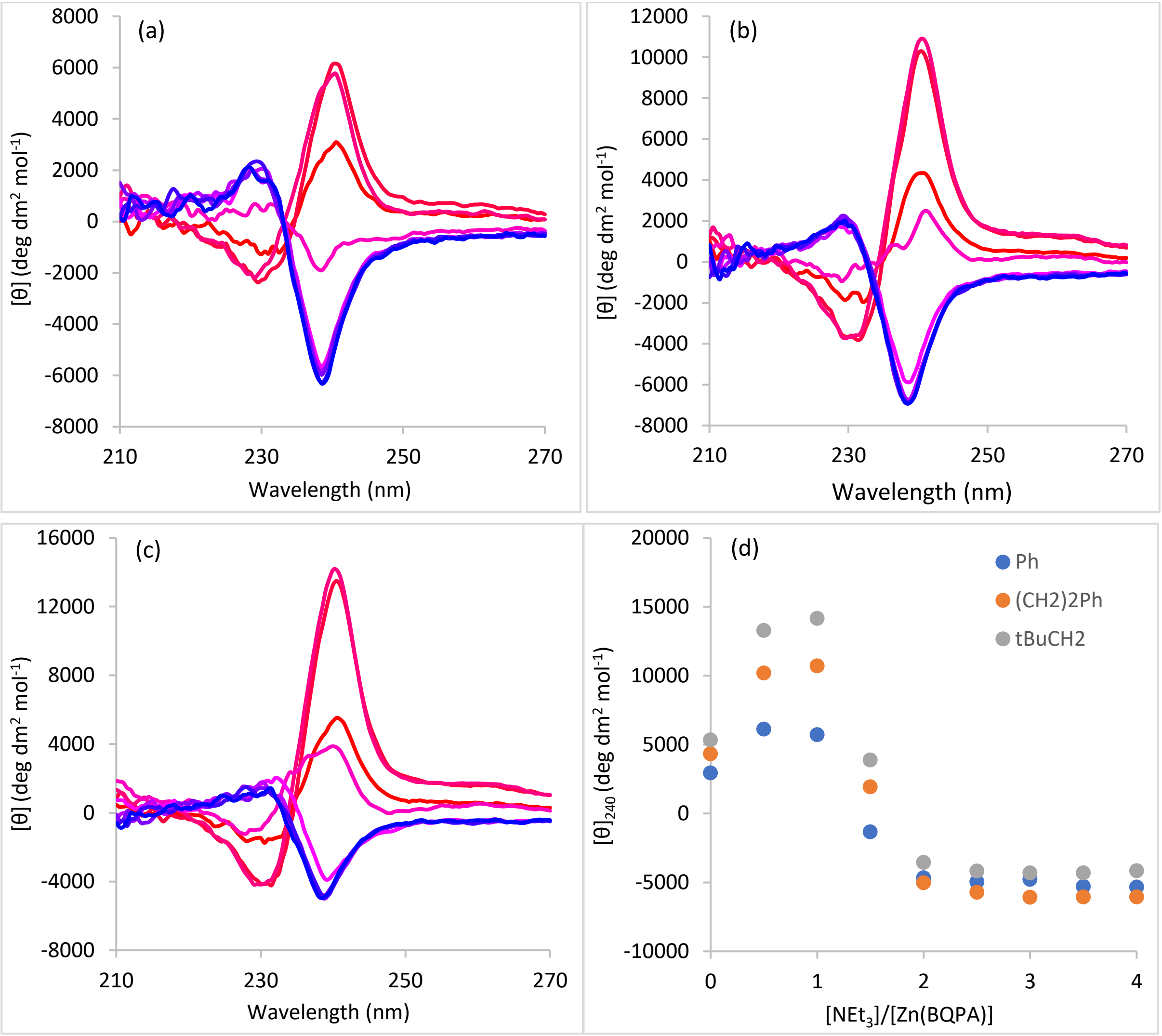

Figure S1. CD spectra of Zn(BQPA)·$2ClO_4$ (c_o = 0.25 mM, MeCN) and (a) **2a** (1 eq.), (b) **2b** (1 eq.) or (c) **2c** (1 eq.) with addition of NEt_3 (**0.0** → **4.0** eq.); (d) Molar ellipticities at 240 nm.

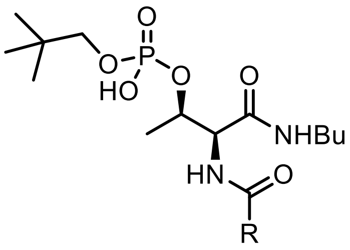

R = CH_2CH_2Ph **2d**
= Me **2e**
= *t*Bu **2f**

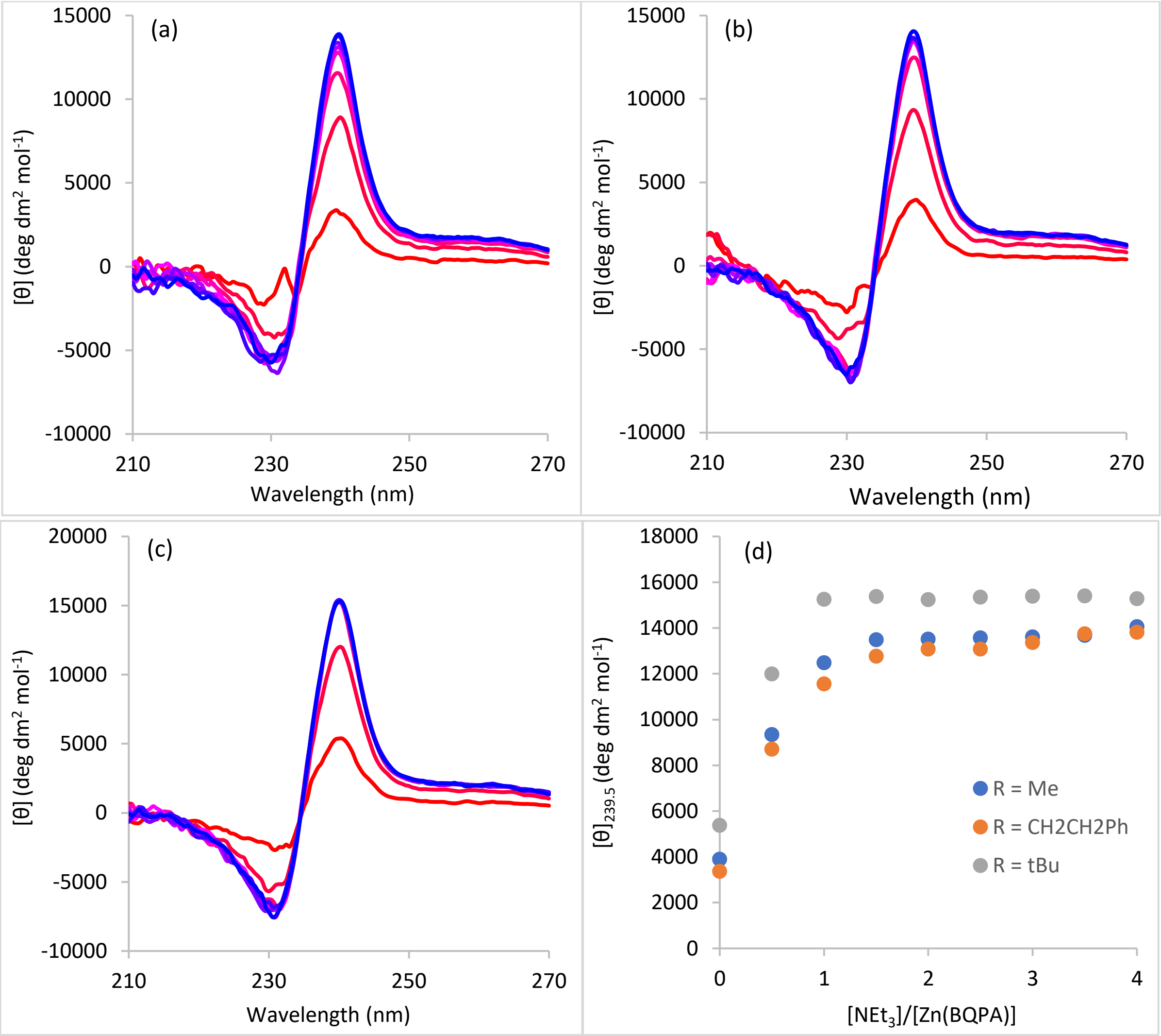

Figure S2. CD spectra of Zn(BQPA)·$2ClO_4$ (c_o = 0.25 mM, MeCN) and (a) **2d** (1 eq.), (b) **2e** (1 eq.) or (c) **2f** (1 eq.) with addition of NEt_3 (**0.0** → **4.0** eq.); (d) Molar ellipticities at 239.5 nm

S3.3 TCAH fuel experiments

S3.3.1 Varying initial TCA^- concentration (Fig. 5a)

An initial sample was prepared in a 1 mm path length quartz cuvette from Zn(BQPA)·$2ClO_4$ (20 µL, 2.50 mM, 50 nmol), ligand **4** (20 µL, 2.50 mM, 50 nmol), NEt_3 (20 µL, see Table **S1**) and MeCN (120 µL). TCAH (20 µL, see Table **S1**) was added and the sample was quickly shaken for homogeneity, then placed into the CD spectrometer sample chamber (temperature set to 25 °C) within 10 seconds. $\theta_{239.5}$ was monitored over 20 minutes, with data points being taken every 10 seconds.

Table S1. Sample compositions for initial [TCA^-] variation. Experiments run in 2% H2O/MeCN at 25 °C.

Component	Volume (µL)	Stock solution concentration (mM)			
		Sample 1	Sample 2	Sample 3	Sample 4
Zn(BQPA)·$2ClO_4$	20	2.5	2.5	2.5	2.5
Ligand **4**	20	2.5	2.5	2.5	2.5
NEt_3	20	5.0	7.5	10.0	12.5
TCAH	20	5.0	7.5	10.0	12.5
MeCN	120	-	-	-	-
**Total volume (µL)**		200	200	200	200
**[TCA^-]/[Zn(BQPA)]**		2.0	3.0	4.0	5.0

S3.3.2 Varying the temperature (Fig. 5b)

An initial sample was prepared in a 1 mm path length quartz cuvette from Zn(BQPA)·$2ClO_4$ (20 µL, 2.50 mM, 50 nmol), ligand **4** (20 µL, 2.50 mM, 50 nmol), NEt_3 (20 µL, 5.0 mM, 100 nmol) and MeCN (120 µL) (Table **S2**). TCAH (20 µL, 5.0 mM, 100 nmol) was added and the sample was quickly shaken for homogeneity, then placed into the sample chamber (temperature set to 20, 25, 30, 35 or 40 °C) within 10 seconds. $\theta_{239.5}$ was monitored over 30 minutes, with data points being taken every 10 seconds. Three runs were performed at each temperature and the average ellipticity taken.

Table S2. Sample composition for temperature variation. Experiments run in 2% H_2O/MeCN at 20, 25, 30, 35 or 40 °C.

Component	Volume (µL)	Stock solution concentration (mM)
Zn(BQPA)·$2ClO_4$	20	2.5
Ligand **4**	20	2.5
NEt_3	20	5.0
TCAH	20	5.0
MeCN	120	-
**Total volume (µL)**		200
**[TCA^-]/[Zn(BQPA)]**		2.0

S3.3.3 Varying water content (Fig. 5c)

An initial sample was prepared in a 1 mm path length quartz cuvette from Zn(BQPA)·2ClO_4 (20 µL, 2.50 mᴍ, 50 nmol), ligand **4** (20 µL, 2.50 mᴍ, 50 nmol), NEt_3 (20 µL, 5.0 mᴍ, 100 nmol), H_2O (see Table **S3**) and MeCN (see Table **S3**). TCAH (10 µL, 10.0 mᴍ, 100 nmol) was added and the sample was quickly shaken for homogeneity, then placed into the sample chamber (temperature set to 35 °C) within 10 seconds. $\theta_{239.5}$ was monitored over 20 minutes, with data points being taken every 10 seconds. Three runs were performed for each water content and the average ellipticity taken.

Table S3. Sample composition for water content variation. Experiments run in 1-6% H_2O/MeCN at 35 °C. a – Note that 10 µL of TCAH solution contains 2 µL H_2O.

Component	Stock solution concentration (mᴍ)	Volume (µL)					
		S1	S2	S3	S4	S5	S6
Zn(BQPA)·2ClO_4	2.5	20	20	20	20	20	20
Ligand **4**	2.5	20	20	20	20	20	20
NEt_3	5.0	20	20	20	20	20	20
TCAH	10.0	10	10	10	10	10	10
H_2O	-	0 (+2)[a]	2 (+2)	4 (+2)	6 (+2)	8 (+2)	10 (+2)
MeCN	-	130	128	126	124	122	120
	**Total volume (µL)**	200	200	200	200	200	200
	**Water content (%)**	1	2	3	4	5	6

S3.3.4 Varying initial TCAH concentration (Fig. 5d)

An initial sample was prepared in a 1 mm path length quartz cuvette from Zn(BQPA)·2ClO_4 (20 µL, 2.50 mᴍ, 50 nmol), ligand **4** (20 µL, 2.50 mᴍ, 50 nmol), NEt_3 (20 µL, 5.0 mᴍ, 100 nmol) and MeCN (120 µL). TCAH (See Table **S4**) was added, and the sample was quickly shaken for homogeneity, then placed into the sample chamber (temperature set to 35 °C) within 10 seconds. $\theta_{239.5}$ was monitored over 20 minutes, with data points being taken every 10 seconds. Three runs were performed for each value of $[TCAH]_o$ and the average ellipticity taken.

Table S4. Sample compositions for initial [TCAH] variation. Experiments run in 2% H_2O/MeCN at 35 °C.

Component	Volume (µL)	Stock solution concentration (mᴍ)				
		S1	S2	S3	S4	S5
Zn(BQPA)·2ClO_4	20	2.5	2.5	2.5	2.5	2.5
Ligand **4**	20	2.5	2.5	2.5	2.5	2.5
NEt_3	20	5.0	5.0	5.0	5.0	5.0
TCAH	20	5.0	7.5	10.0	12.5	15.0
MeCN	120	-	-	-	-	-
**Total volume (µL)**		200	200	200	200	200
**$[TCAH]_o$/[Zn(BQPA)]**		2.0	3.0	4.0	5.0	6.0

S3.3.5 Multiple fuel pulses (Fig. 5e)

An initial sample was prepared in a 10 mm path length quartz cuvette from Zn(BQPA)·2ClO$_4$ (20 µL, 2.50 mM, 50 nmol), ligand **4** (20 µL, 2.50 mM, 50 nmol), NEt$_3$ (20 µL, 5.0 mM, 100 nmol), H_2O (40 µL) and MeCN (1900 µL). A pulse of TCAH (See Table **S5**) was added and the sample was quickly shaken for homogeneity, then placed into the sample chamber (temperature set to 35 °C) within 10 seconds. $\theta_{239.5}$ was monitored over 10 minutes, with data points being taken every 2 seconds. Four further pulses of TCAH were added, with a 10-minute observation period after each addition.

Table S5. Sample composition for addition of multiple fuel pulses. Experiments run at 35 °C. a – cumulative volume of H_2O contained in the sample after each pulse of fuel, which contained 20 vol% H_2O

Component	Stock solution concentration (mM)	Volume (µL)					
		Initial	Pulse 1	P2	P3	P4	P5
Zn(BQPA)·2ClO$_4$	2.5	20	-	-	-	-	-
Ligand **4**	2.5	20	-	-	-	-	-
NEt$_3$	5.0	20	-	-	-	-	-
TCAH	10.0	0	5.0	10.0	15.0	2.5	7.5
H_2O	-	40	(41)^a	(43)^a	(46)^a	(46.5)^a	(48)^a
MeCN	-	1900	-	-	-	-	-
	**Total volume (µL)**	2000	2005	2015	2030	2032.5	2040
	**Water content (%)**	2.00	2.04	2.13	2.27	2.29	2.35

S3.3.6 Assessing performance drift

In the multiple fuel pulse experiment (Fig. 5e), the water content increased from 2.00% to 2.35% after five fuel pulses of varying volume (section S3.3.5). Water was known to influence the decomposition rate of TCA$^-$ (Fig. 6c), so a more extensive multiple fuel pulse experiment was performed to assess the degree to which each fuel pulse affects the performance of the automaton.

An initial sample was prepared in a 10 mm path length quartz cuvette from Zn(BQPA)·2ClO$_4$ (20 µL, 2.50 mM, 50 nmol), ligand **4** (20 µL, 2.50 mM, 50 nmol), NEt$_3$ (20 µL, 5.0 mM, 100 nmol), H_2O (40 µL) and MeCN (1900 µL) and the ellipticity at 239.5 nm recorded over 5 minutes. A pulse of TCAH (10 µL, 10.0 mM, 100 nmol) was added, and the sample was quickly shaken for homogeneity then placed into the sample chamber (temperature set to 35 °C) within 10 seconds. $\theta_{239.5}$ was monitored over 10 minutes, with data points being taken every 2 seconds. 9 further pulses of TCAH (10 µL, 10.0 mM, 100 nmol) were added, with a 10 minute observation period after each addition except for the final three pulses, which used a 15 minute observation period.

A plot of $\theta_{239.5}$ against time revealed a steady decrease in both operating rate and output magnitude of the two stations (Fig. S3). Since the total volume of fuel added was 100 µL, the total sample volume increased from 2000 µL to 2100 µL, reducing the concentration of the [Zn(BQPA)]$^{2+}$ receptor from

25.0 µM to 23.8 µM. This concentration change accounts for the decrease in output magnitude. Although this can be corrected for by converting $\theta_{239.5}$ to molar values, it is important to acknowledge that the *raw* CD output is reduced by multiple fuel pulses. The water concentration in the sample increased from 2.00% to 2.86% after 10 pulses, since the fuel contained 20 vol% water. The deceleration of operating rate is therefore consistent with the results in Fig. 5c, where varying the water content of the sample by as little as 1% had a significant effect on the TCA^- decomposition rate.

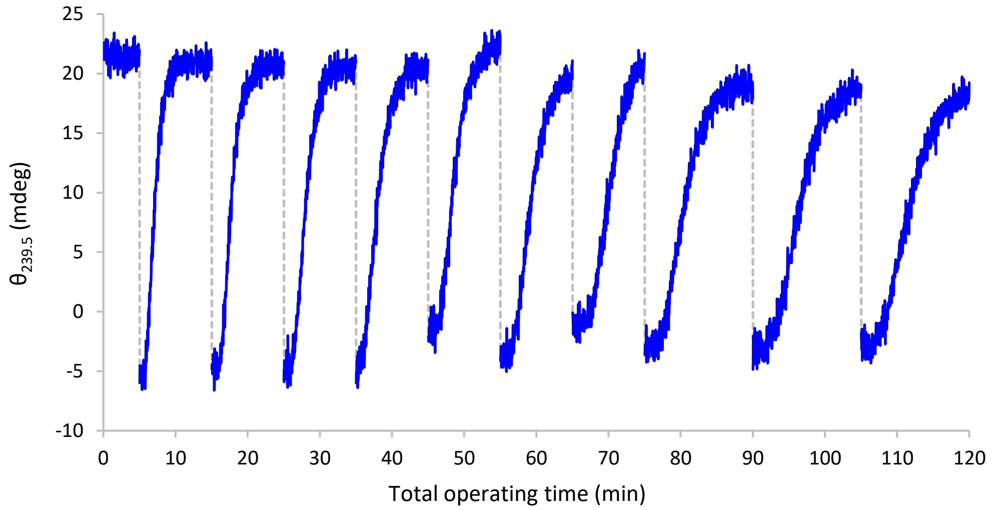

Figure S3. Time-course ellipticity measurement at 239.5 nm of Zn(BQPA)·$2ClO_4$ (c_o = 0.025 mM), **4** (1 eq.) and NEt_3 (2 eq.) in 2% H_2O/MeCN at 35 °C with 10 consecutive pulses of 2.0 eq. TCAH.

In principle, these performance drift effects can be mitigated by increasing the initial sample volume, which reduces the concentration changes caused by TCAH addition. Additionally, using a more concentrated TCAH solution would allow a smaller volume of fuel to be added for each pulse. Of course, the ideal solution would be to find a fuelling system in which the solvent composition of the fuel is identical to that of the sample. In this case, using 20 vol% in the sample may slow TCA^- decomposition too much for the automaton to operate, but this may be counterbalanced by increasing the operating temperature or initial base concentration. This demonstrates the power and versatility of having several independent variables that can control the rate of TCA^- decomposition.

S3.4 Binding constant measurements

Titration experiments were carried out with Zn(BQPA)·$2ClO_4$ using test ligands **2f** and **3** to determine their binding constants and hence estimate the binding selectivity for the phosphate and carboxylate sites in ligand **4**.

A 2000 µL sample of Zn(BQPA)·$2ClO_4$ (*c* = 25.0 µM, 2:98 H_2O:MeCN) in a 10 mm path length cuvette was titrated with a solution composed of ligand **3** (1.50 mM), phosphazene base tBuP$_1$(NMe$_2$)$_3$ (1.50 mM), Zn(BQPA)·$2ClO_4$ (25.0 µM) in 2:98 H_2O:MeCN at 25 °C. tBuP$_1$(NMe$_2$)$_3$ was chosen as the base for its high basicity (pK_a(MeCN) = 26.98)[11] to ensure quantitative deprotonation of **3** and to minimise hydrogen bonding effects arising from the cationic conjugate acid. Having 25.0 µM Zn(BQPA)·$2ClO_4$ in

the ligand solution ensured a constant host concentration throughout the titration experiment, thus eliminating any potential dilution effects.

The ellipticity at 239.5 nm was recorded after each addition of ligand solution, for a total of 20 data points between 0 and 120 µL of total added solution, corresponding to 0 and 3.4 equivalents of ligand. The titration data was fit to a 1:1 binding model using BindFit with the Nelder-Mead method;[12] a representative binding curve and fit is shown in fig. **S4**. An average of three experiments gave a binding constant of ***K* = 1.19 ± 0.15 × 10^{6} M^{-1}**, where the uncertainty is the standard deviation from the mean (Table **S6**).[13]

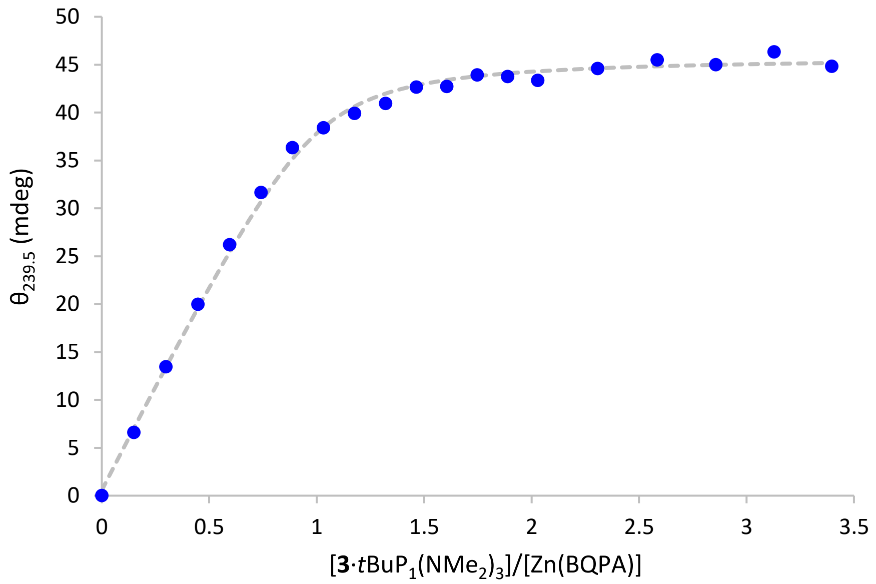

Figure S4. Experimental data (blue points) and 1:1 fit (grey dashed line) from a titration of Zn(BQPA)·2ClO$_4$ (*c* = 25.0 µM) with a 1:1 mixture of ligand **3** and *t*BuP$_1$(NMe$_2$)$_3$ in 2:98 H_2O:MeCN at 25 °C.

Table S6. Results from fitting data from titration of Zn(BQPA)·2ClO$_4$ (*c* = 25.0 µM) with 1:1 mixture of ligand **3** and *t*BuP$_1$(NMe$_2$)$_3$ in 2:98 H_2O:MeCN at 25 °C to a 1:1 binding model.

**Experiment**	***K* (10^{6} M^{-1})**
1	1.34
2	1.20
3	1.04
Mean	1.19
Standard Deviation	0.15

Titration of Zn(BQPA)·2ClO$_4$ with a 1:1 mixture of **2f** and *t*BuP$_1$(NMe$_2$)$_3$ gave typical tight-binding curves (i.e. a linear segment between 0 and 1 equiv ligand followed by a plateau beyond 1 equiv of ligand) at c(Zn(BQPA)) = 0.25 or 0.05 mM (Fig. **S5**, red and yellow points, respectively). However, when the binding titration was performed at c(Zn(BQPA)) = 25.0 µM, the expected 1:1 binding curve was not observed (Fig. **S5**, blue points). While the general appearance of the curve superficially resembled that

of 1:1 binding, the initial gradient was too steep for this binding model and BindFit failed to fit the data with acceptable error values. Similarly, the data could not be adequately fit to the 1:2 or 2:1 binding model either. This suggests that the binding interaction between **2f** and $[Zn(BQPA)]^{2+}$ is more complex than simple coordination of a phosphate oxygen atom to the Zn^{2+} centre. Although the precise nature of the binding interaction between **2f** and $[Zn(BQPA)]^{2+}$ is not known, it is still likely that a $[Zn(BQPA)]^{2+}$ receptor binds, at most, one equivalent of **2f** as we have previously shown that multiple anion binding to $[Zn(BQPA)]^{2+}$ leads to a reduction in its CD output.[1]

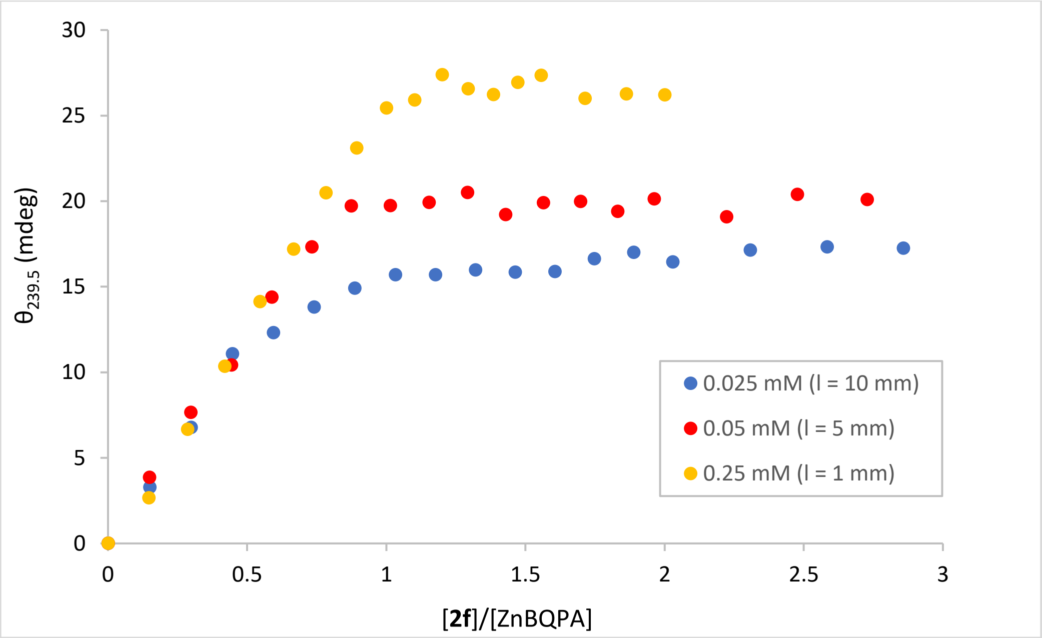

Figure S5: Ellipticity at 239.5 nm from a titration of $Zn(BQPA)\cdot 2ClO_4$ (c = 0.25 mM, 0.05 mM or 0.025 mM) with a 1:1 mixture of **2f** and tBuP$_1$(NMe$_2$)$_3$ in 2:98 H_2O:MeCN at 25 °C.

S4 Calculation of pK_a values of ligands **2f** and **3**

pK_a values in pure acetonitrile were calculated using the COSMO-RS method[14–16] and were empirically corrected. COSMO-RS calculation consists of two steps: (1) geometry optimisation in an ideal conductor with DFT calculations, yielding total energy and partial charge distribution over the molecular surface, and (2) statistical thermodynamic calculation considering intermolecular interactions in solution via pairwise interactions of molecular surface segments.

To simplify calculations, ligand **4** was split into the following model compounds **27** and **28**:

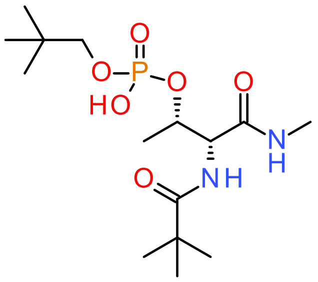

Model phosphate **27** for station 1

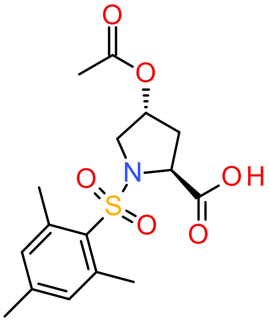

Model carboxylate **28** for station 2

Different starting geometries were created to identify all stable conformers of the neutral and anionic forms. The geometries were optimized in ideal conductor at DFT BP TZVP level with RI approximation. Vibrational spectra were calculated to verify that the geometries correspond to local energy minima; if imaginary (negative) frequencies were found, the geometries were adjusted and re-optimized. Negative frequencies with wave numbers down to –20 cm^{-1} were allowed, conformers with persistent lower imaginary wave numbers were excluded. After that, single-point calculations were carried out at BP TZVPD level of theory with *Fine cavity* parameter. All DFT calculations were carried out using Turbomole V7.2[17] software.

pK_a values in acetonitrile were calculated using the standard feature of COSMOtherm (Release 2021, parametrization BP_TZVPD_FINE_21)[18] software. All conformers chosen in the previous step were taken into account. The calculated values were empirically corrected as described in ref. [1]; the reliable experimental pK_a values of a number of similar compounds were compared with the corresponding calculated values (Table **S7**), and corrections (multiplicative and/or additive) were applied. The standard error of regression was used to estimate the standard uncertainty (*u*) of the corrected values. The pK_a estimates thus obtained are **15.0 ± 2.0** for phosphate **27** and **21.8 ± 1.3** for carboxylate **28** (Table **S8**). Thus, the difference between the pK_a values of the stations is most likely over 3 pK_a units.

Table S7. Experimental and calculated pK_a values for selected reference acids. *a* – Calculated pK_a values using COSMO-RS. *b* – Experimental pK_a values taken from ref. [19] unless stated otherwise. *c* – Difference between calculated and experimental pK_a. BPA = BINOL phosphoric acid

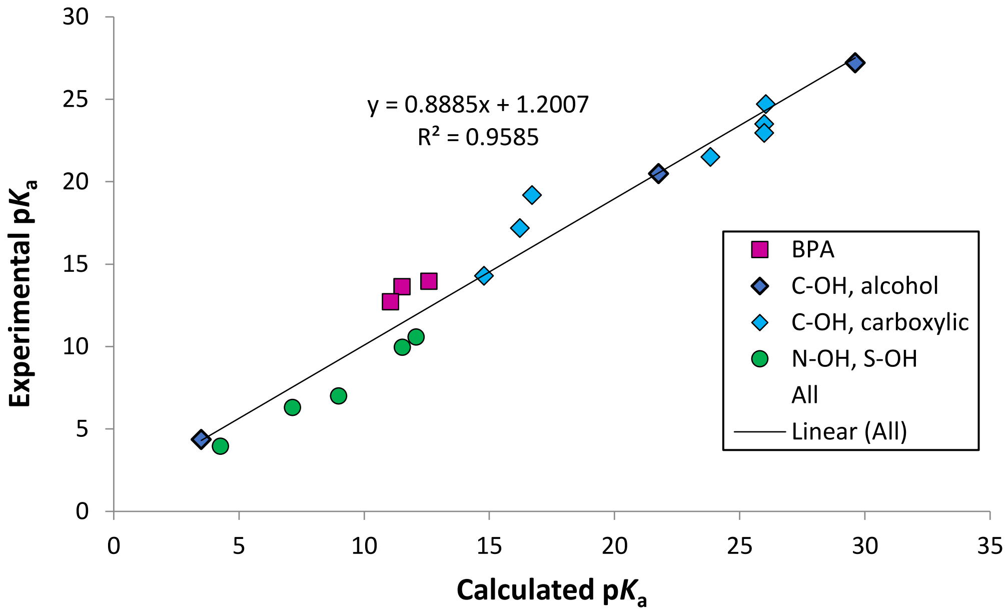

Compound	CAS	Type	Calc.[a]	Exp.[b]	Error[c]
[9-Phenanthrene]-BPA-[H_8]	934201-93-1	BPA (P-OH)	12.6	13.97	-1.4
[2,4,6-*i*-Pr_3-Ph]-BPA	791616-63-2	BPA (P-OH)	11.5	13.63	-2.1
[Ph]-BPA	695162-86-8	BPA (P-OH)	11.1	12.73	-1.7
$(3,5–(CF_3)_2–C_6H_3)_3COH$	2376448-26-7	C-OH	29.6	27.21	2.4
$(CF_3)_3COH$	2378-02-1	C-OH	21.8	20.5	1.3
$(CN)_2C=C(CN)OH$	27062-39-1	C-OH	3.5	4.36	-0.9
Acetic acid	64-19-7	C-OH	26.0	23.5	2.5
Sorbic acid	110-44-1	C-OH	26.0	22.97	3.0
Cyclohexanecarboxylic acid	98-89-5	C-OH	26.0	24.7[20]	1.3
Benzoic acid	65-85-0	C-OH	23.8	21.5	2.3
Br_4–CA–benzoic acid	1269510-74-8	C-OH	16.7	19.2	-2.5
Br_4–CX–benzoic acid		C-OH	16.2	17.19	-1.0
Phthalic acid	88-99-3	C-OH	14.8	14.3[21]	0.5
Nitric acid	7697-37-2	N-OH	12.1	10.6[22]	1.5
Me-SO_3H	75-75-2	S-OH	11.5	10.0	1.6
4-Cl-C_6H_4-SO_3H	98-66-8	S-OH	9.0	7.0	2.0
4-NO_2-C_6H_4-SO_3H	138-42-1	S-OH	7.1	6.3	0.8
2,4-$(NO_2)_2$-C_6H_3-SO_3H	89-02-1	S-OH	4.3	3.96	0.3

Table S8. Calculated and corrected pK_a data for model compounds **27** and **28**, with standard uncertainty estimates (*u*)

Model Compound	Calc.	Predicted	*u*	Correction	Assigned pK_a	*u*
**27**	15.3	14.8	1.5	all OH, correlation	**15.0**	**±2.0**
		17.0		BPA, additive		
**28**	23.2	21.8	1.5	all OH, correlation	**21.8**	**±1.3**
		21.8	1.3	C-OH, correlation		
		21.8	1.3	carboxylic, correlation		

The effect of water on pK_a can be rationalized using the degree of charge delocalization in an anionic conjugate base.[23] If charge delocalization in the conjugate bases of two acids is similar, the pK_a difference of these acids will not change significantly upon addition of a small amount of water, even if the acids are of different types (e.g. phenol vs diphenylacetonitrile). This was demonstrated for water contents up to ~1% (by mass) by using weighted average positive sigma (WAPS) values as computational descriptors of charge delocalization in anions.[23] It was shown that addition of ~1% of water to acetonitrile changed the pK_a difference of two acids by 1 and more units if WAPS difference of anions was >5.

WAPS values were obtained from the results of DFT calculations; values of conformers were weighted by relative stabilities of the conformers in acetonitrile with 2% water (computed with COSMO-RS method). WAPS values of anions of model compounds 1 and 2 are 2.1 and 2.6, respectively. It is safe to conclude that in this case the addition of up to 1% of water to acetonitrile will have only a limited influence on the difference of their pK_a values.

The following table shows the effect of 40% of water on the acidity differences of OH-type acids:

Acid 1	Acid 2	ΔpK_a(MeCN)[19]	Δ_s^spK_a (60% MeCN 40% water)[24]
Phenol	Acetic acid	29.2 – 23.5 = 5.7	12.38 – 6.57 = 5.81
Phenol	Benzoic acid	29.2 – 21.5 = 7.7	12.38 – 6.25 = 6.13
Acetic acid	Benzoic acid	23.5 – 21.5 = 2.0	6.57 – 6.25 = 0.32

These data support the conclusion that the addition of 2% of water will not lead to a dramatic change in pK_a difference between stations 1 and 2 of ligand **4**, as changing the water content from 0% to 40% gave, at most, a change in pK_a difference of 1.6 units.

S5 ^{1}H and ^{13}C NMR spectra of novel compounds

^{1}H NMR (400 MHz, $CDCl_3$) of Compound **6**

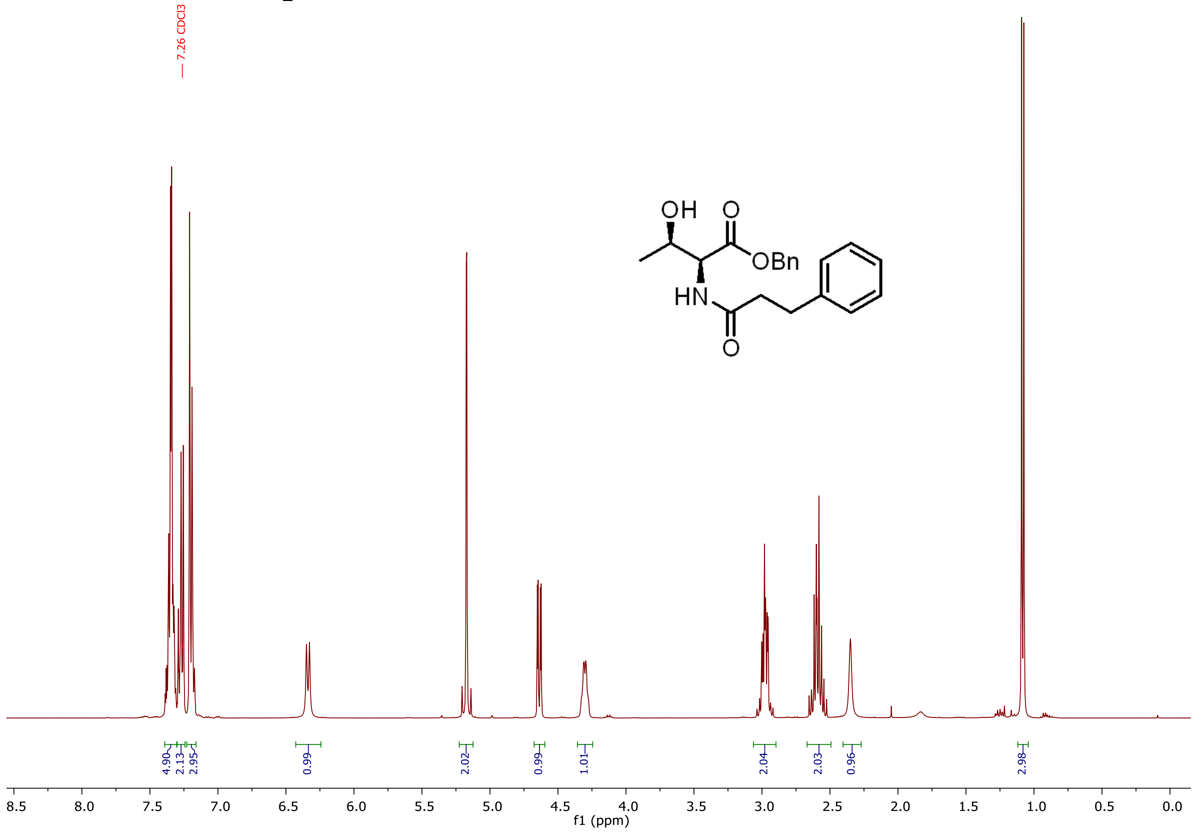

^{13}C NMR (101 MHz, $CDCl_3$) of Compound **6**

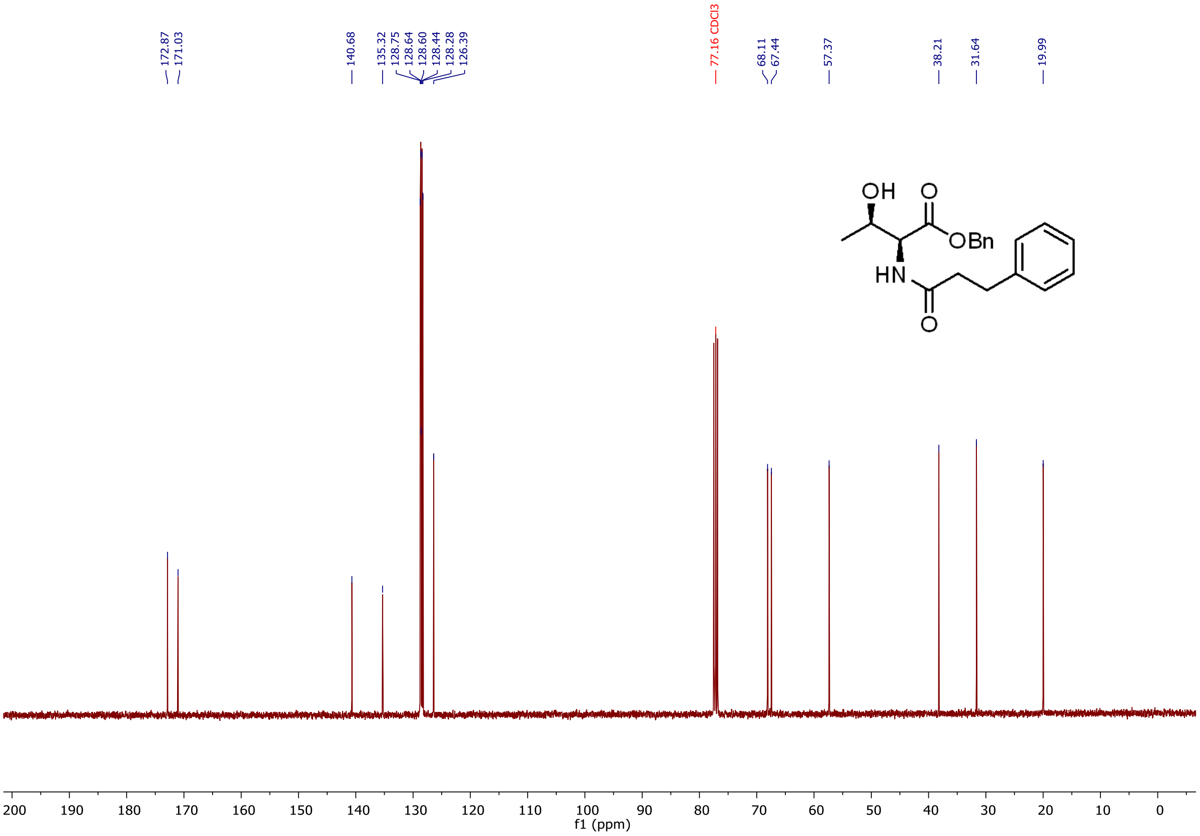

^{1}H NMR (400 MHz, $CDCl_3$) of Compound **8**

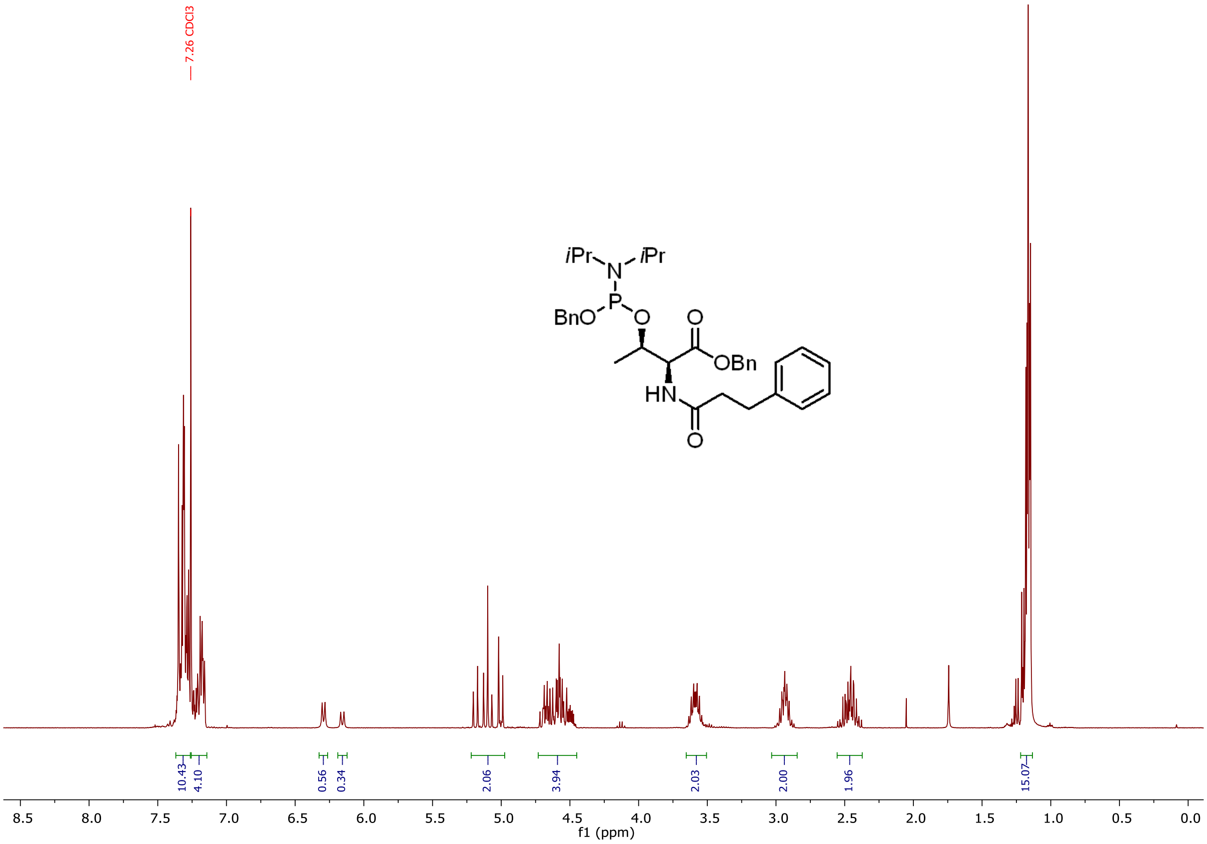

^{13}C NMR (101 MHz, $CDCl_3$) of Compound **8**

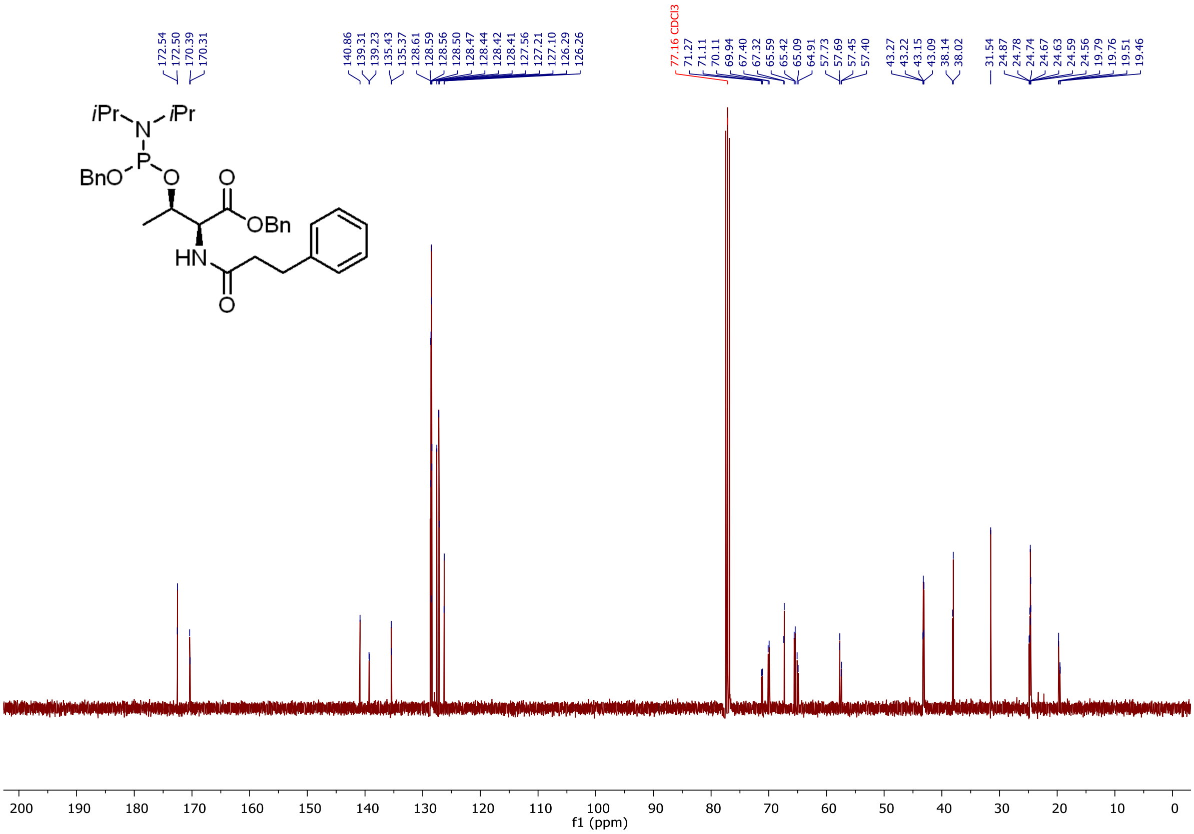

^{1}H NMR (400 MHz, $CDCl_3$) of Compound **9a**

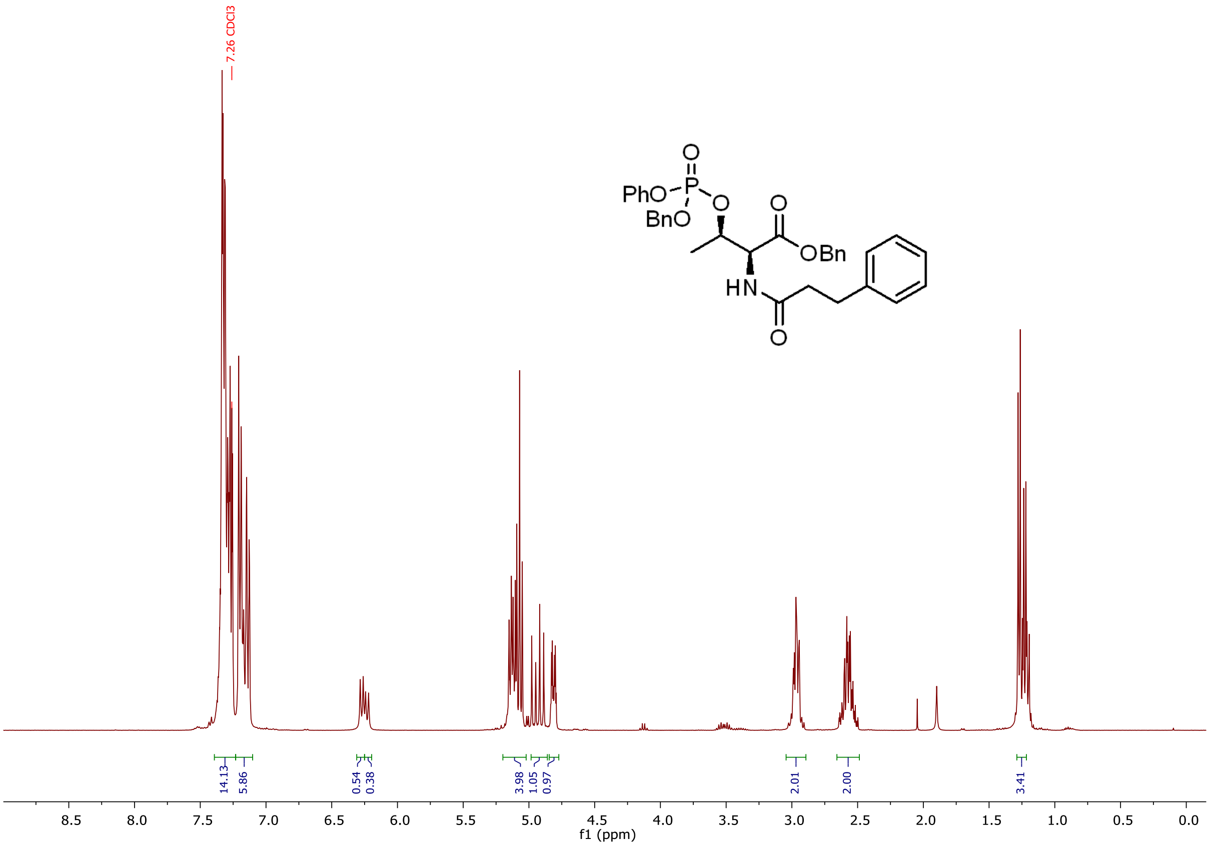

^{13}C NMR (101 MHz, $CDCl_3$) of Compound **9a**

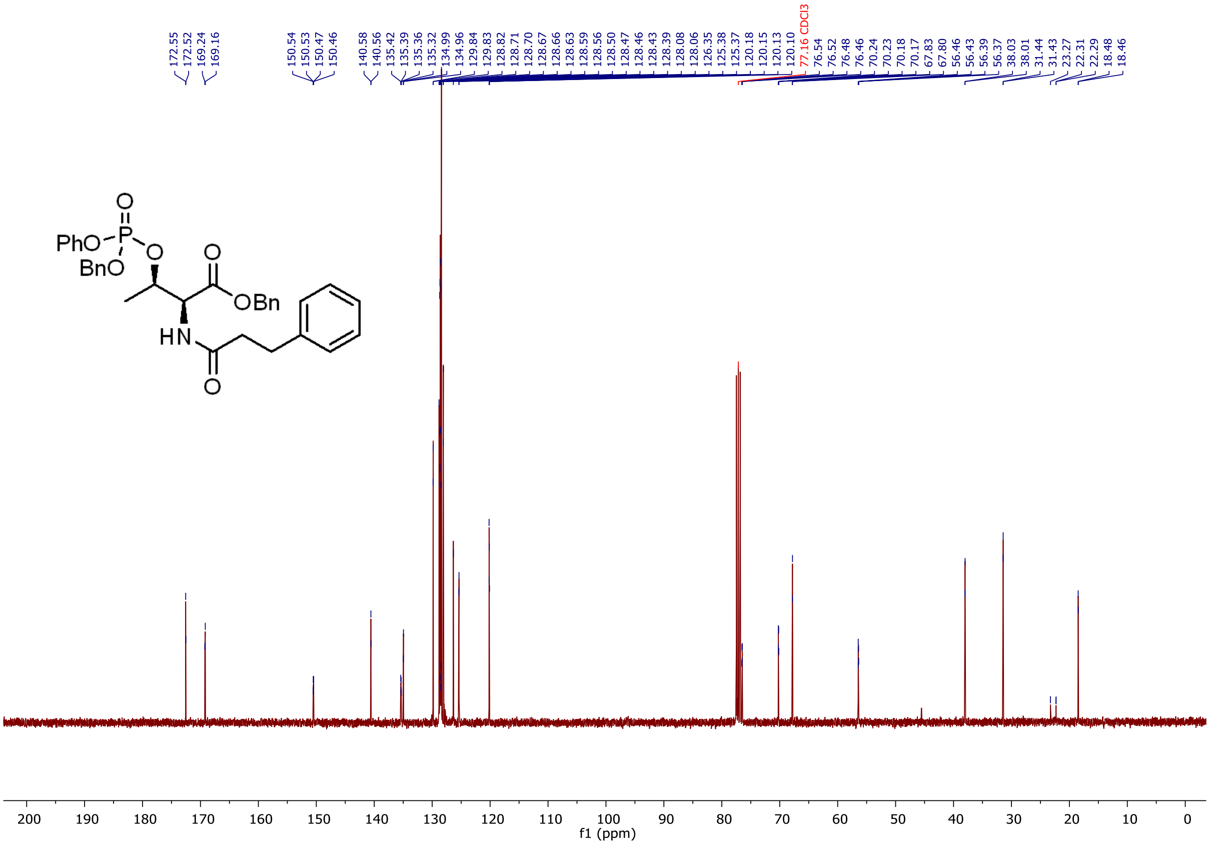

^{1}H NMR (400 MHz, $CDCl_3$) of Compound **9b**

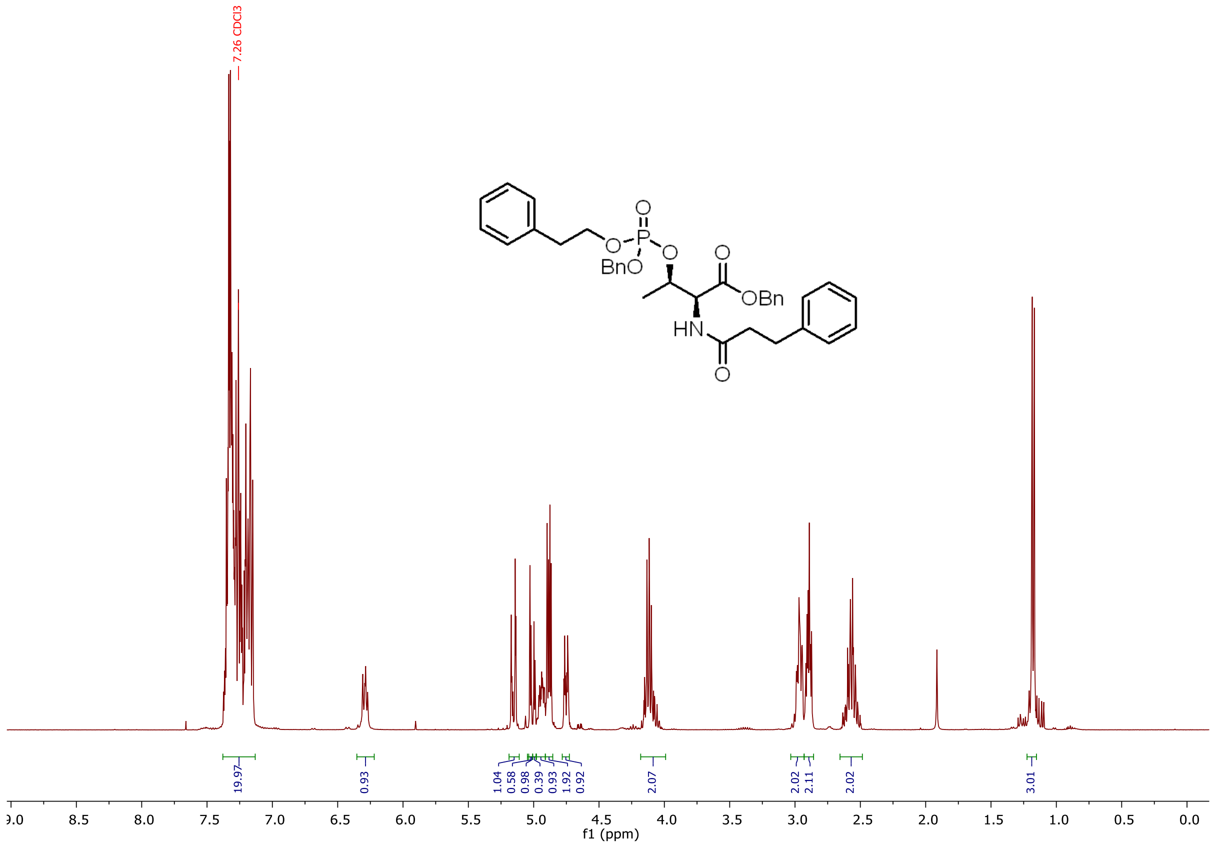

^{13}C NMR (101 MHz, $CDCl_3$) of Compound **9b**

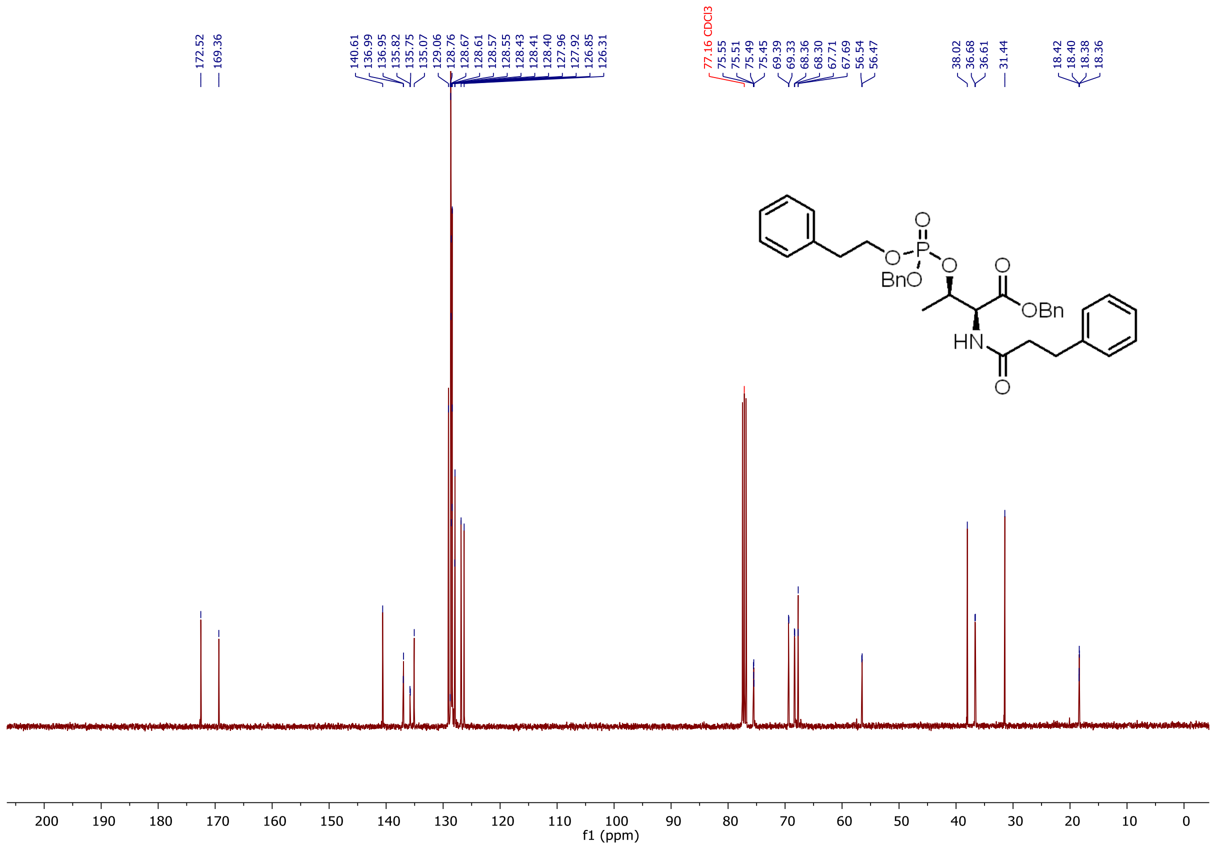

^{1}H NMR (400 MHz, $CDCl_3$) of Compound **9c**

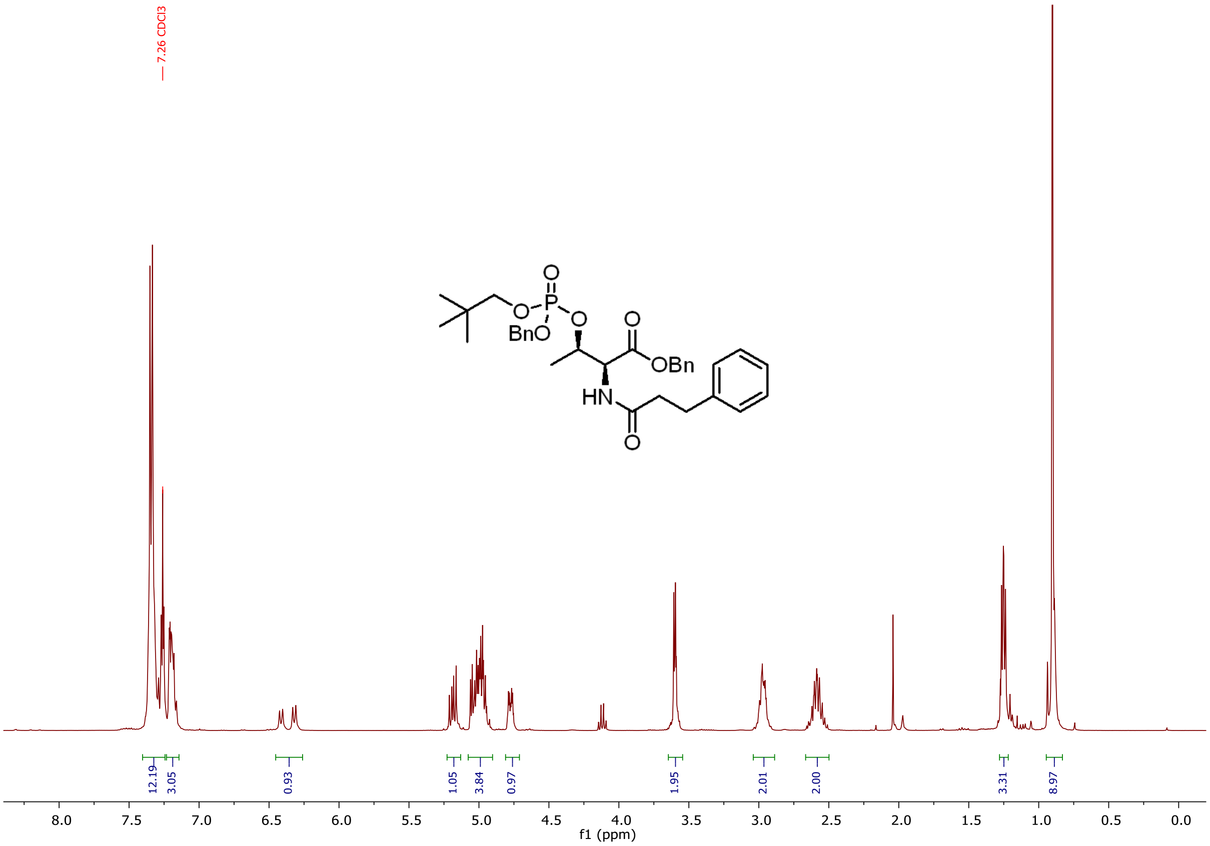

^{13}C NMR (101 MHz, $CDCl_3$) of Compound **9c**

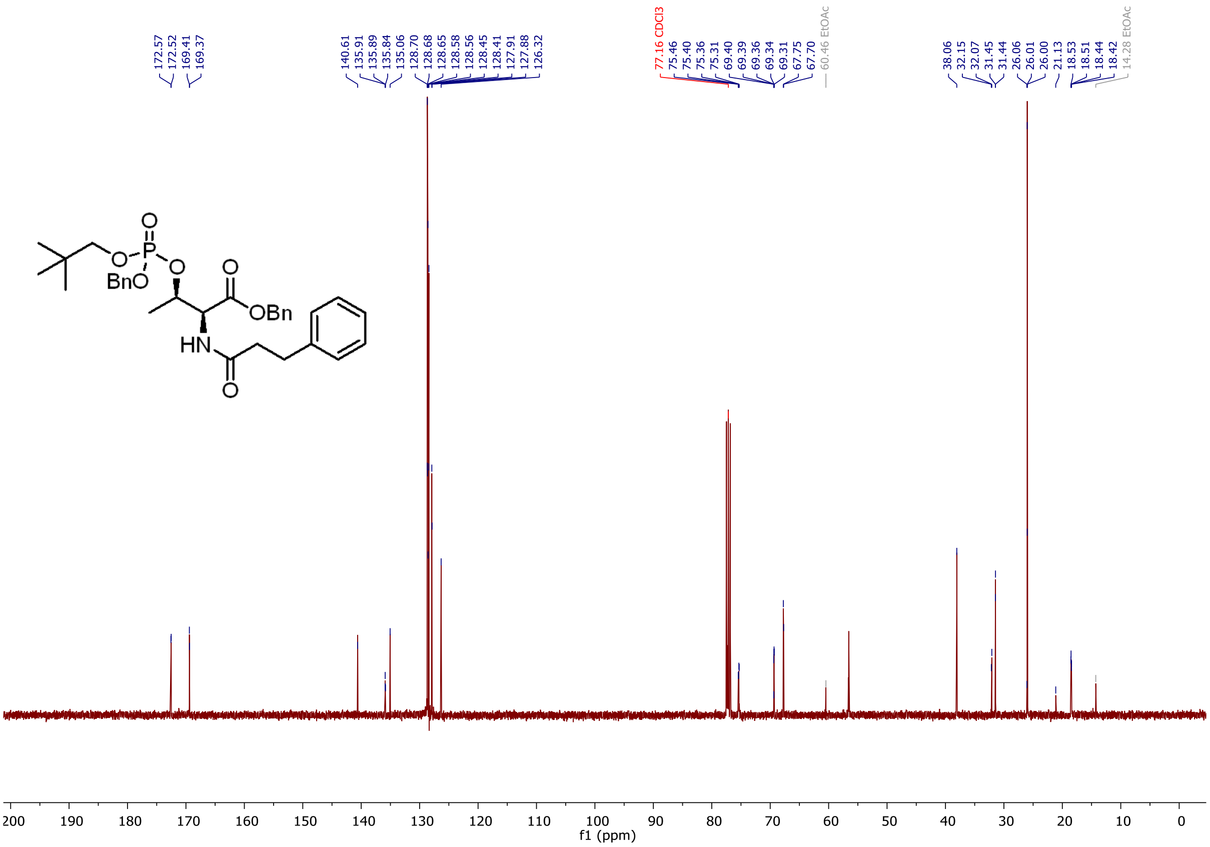

^1H NMR (400 MHz, CD_3OD) of Compound **2a**

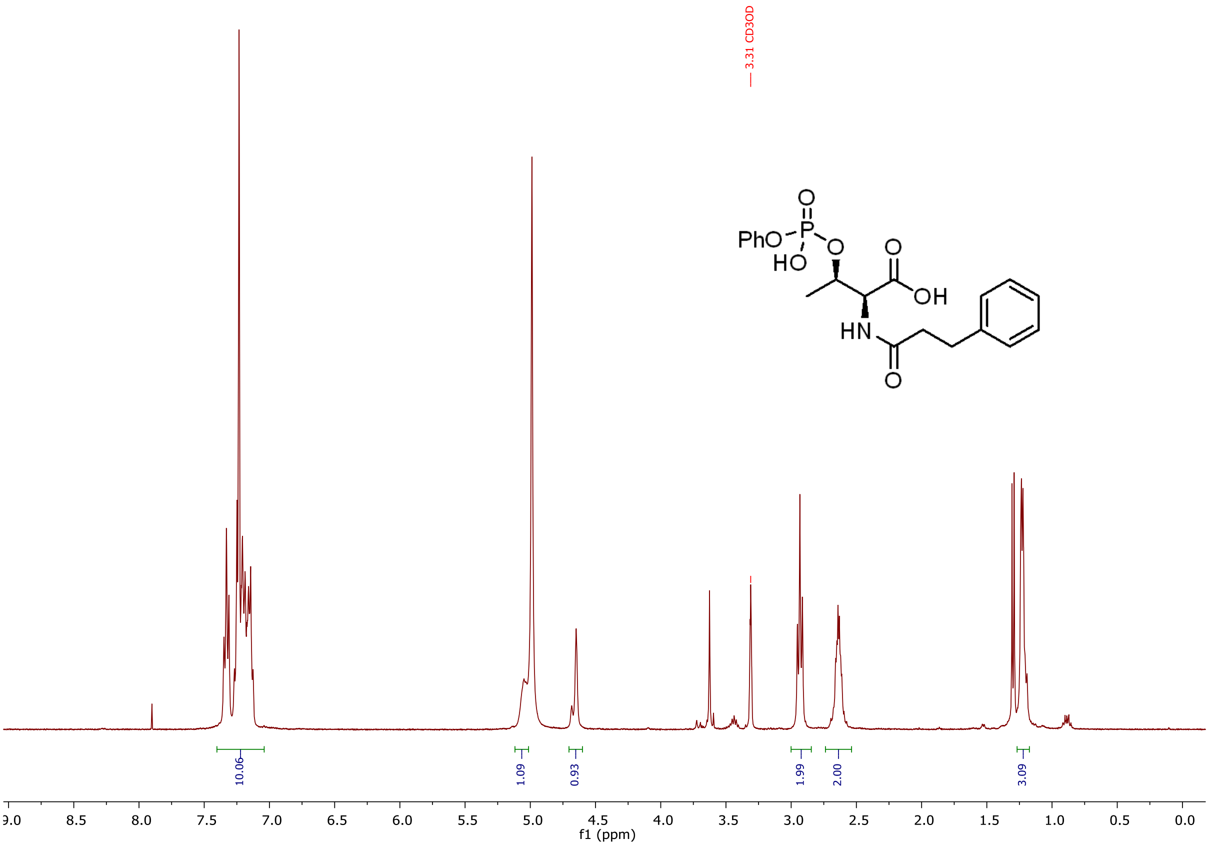

^{13}C NMR (101 MHz, CD_3OD) of Compound **2a**

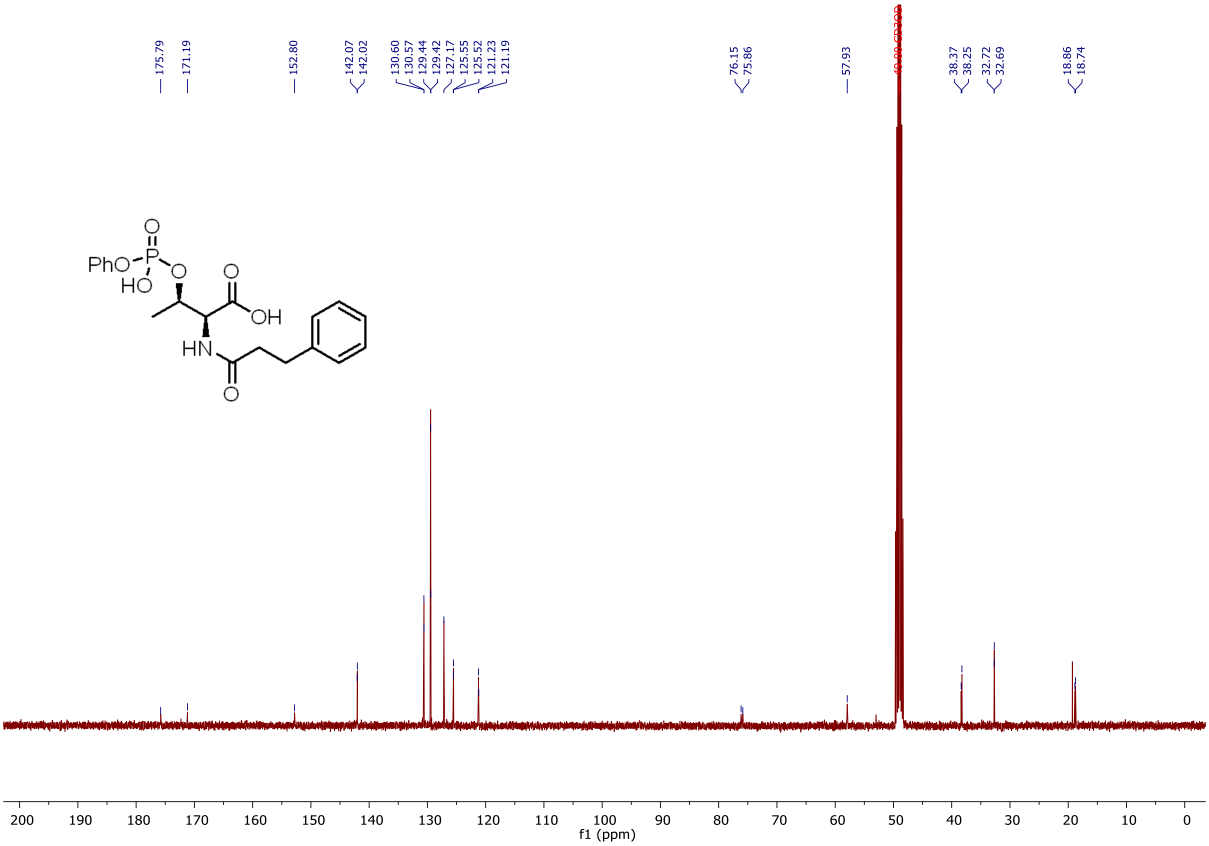

^{1}H NMR (400 MHz, CD_3OD) of Compound **2b**

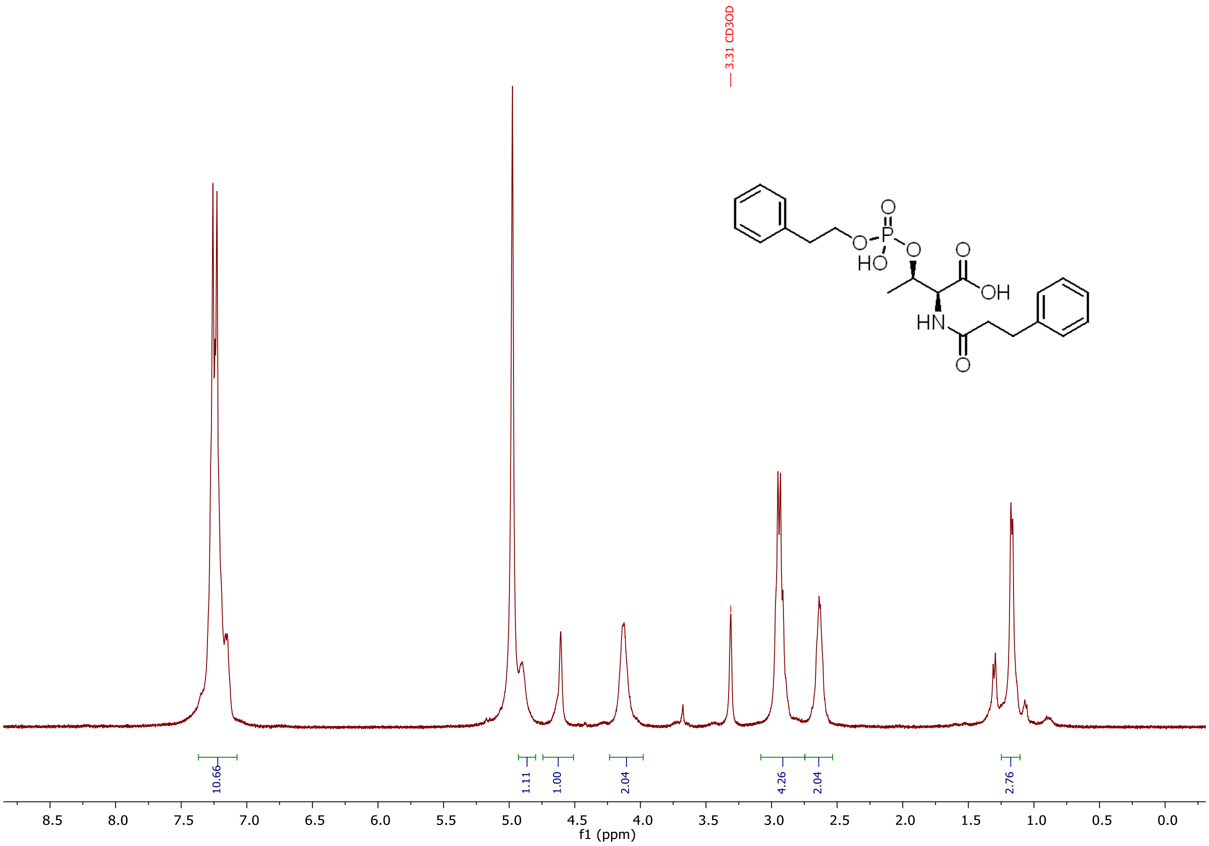

^{13}C NMR (101 MHz, CD_3OD) of Compound **2b**

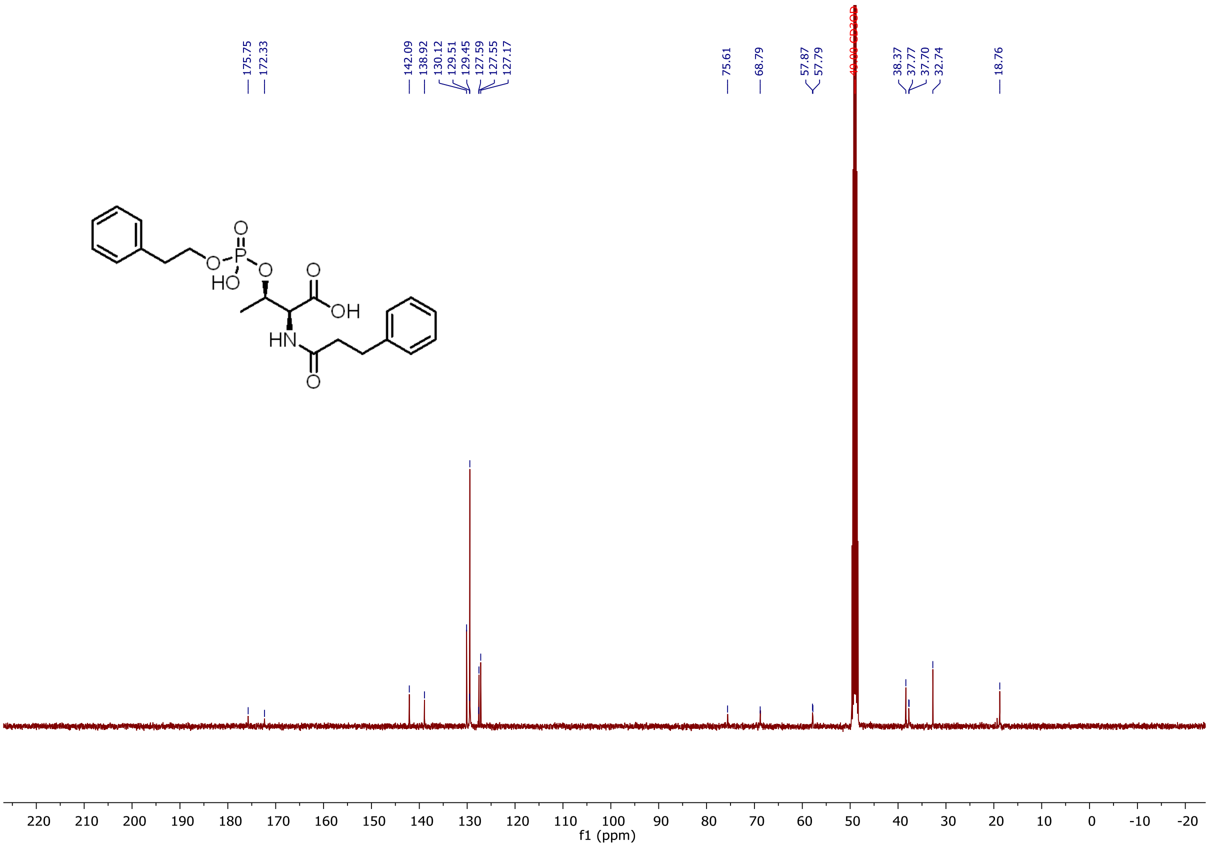

^{1}H NMR (400 MHz, CD_3OD) of Compound **2c**

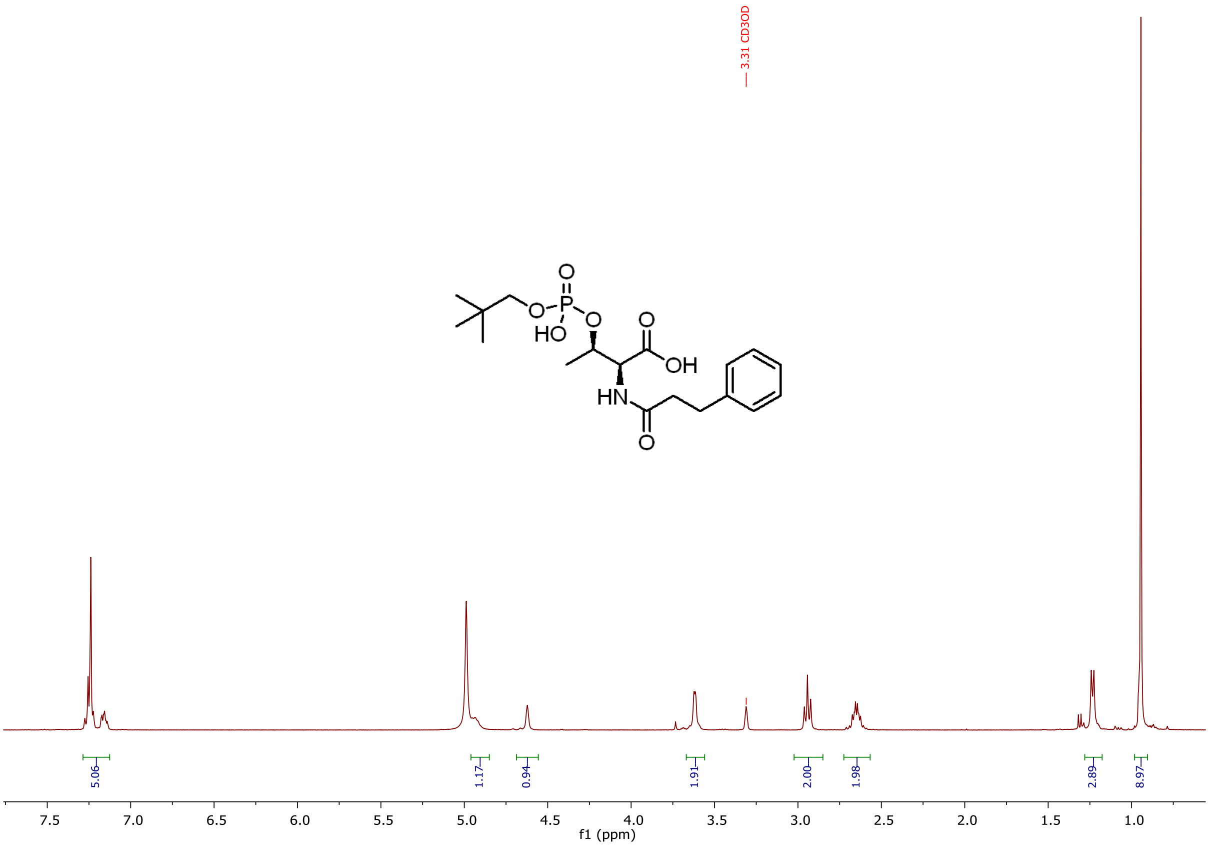

^{13}C NMR (101 MHz, CD_3OD) of Compound **2c**

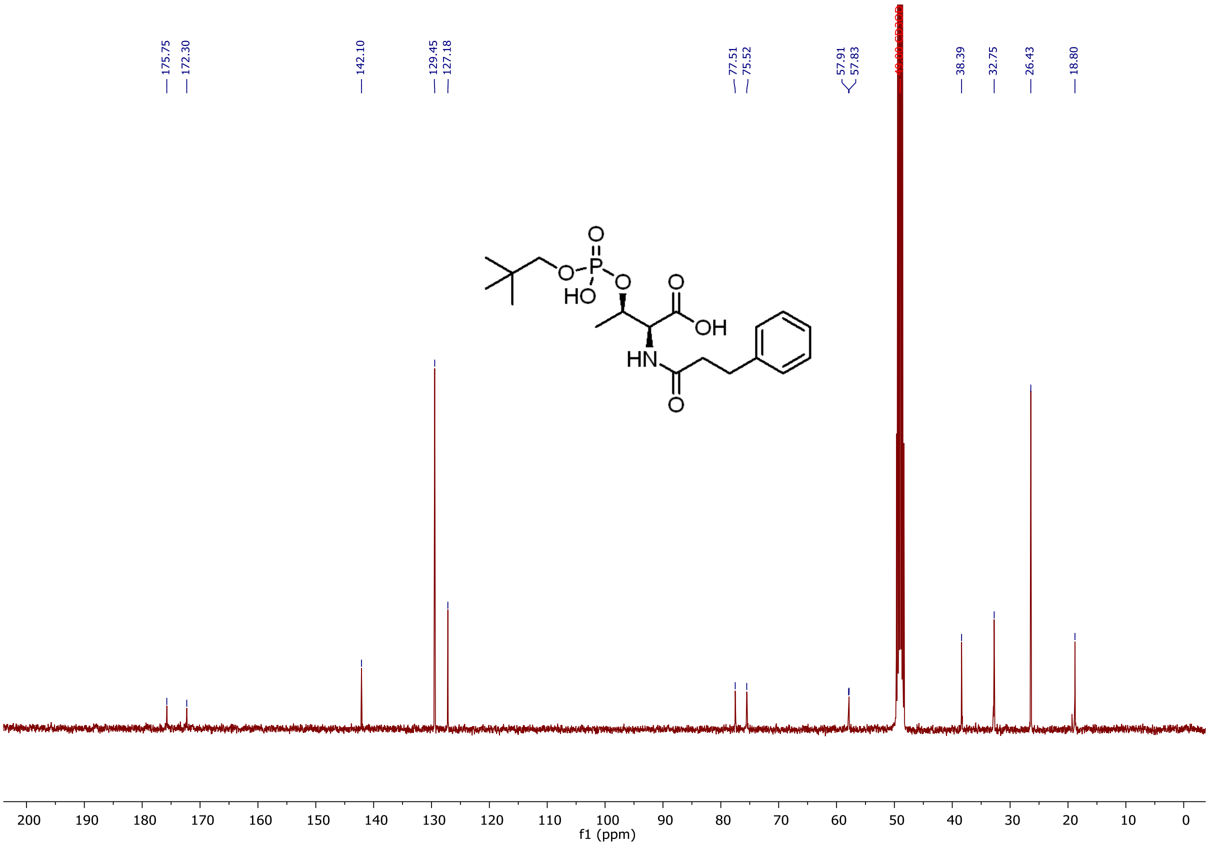

^{1}H NMR (400 MHz, $CDCl_3$) of Compound **11**

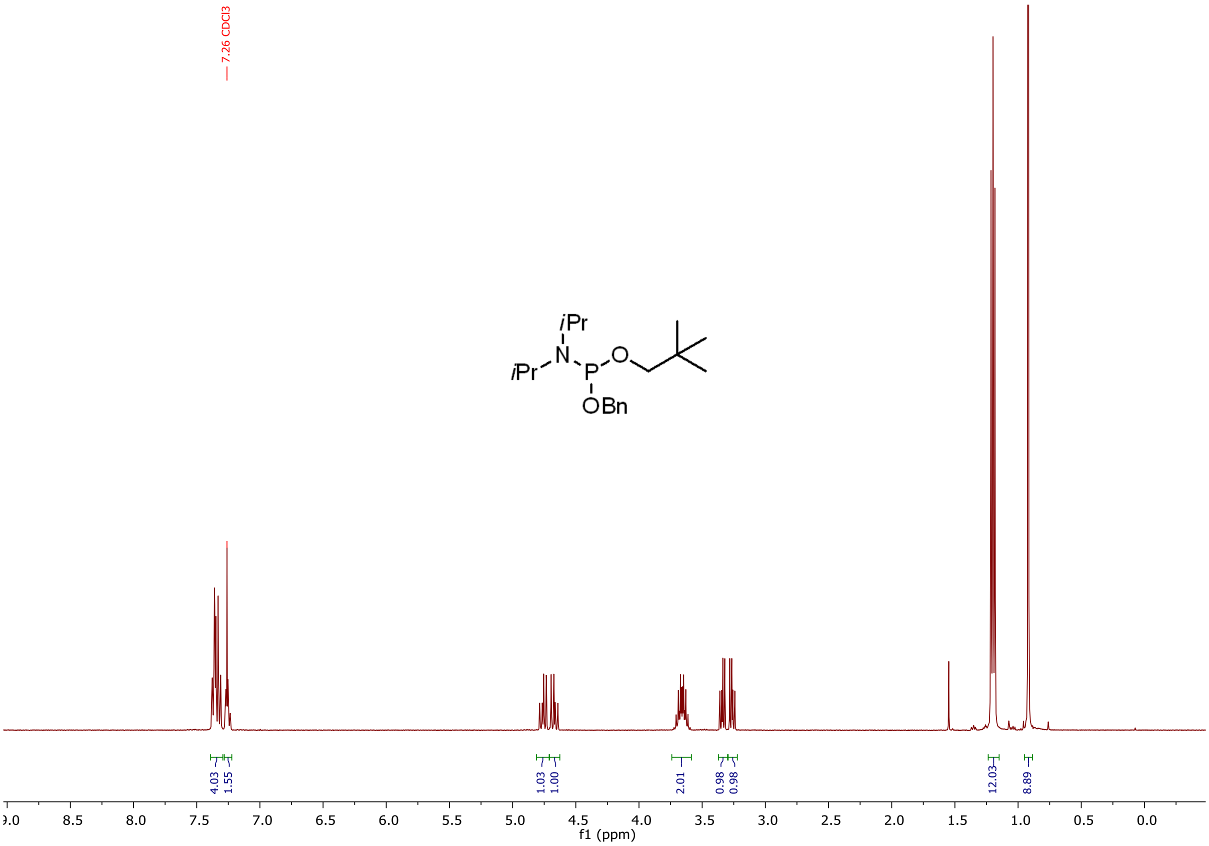

^{13}C NMR (101 MHz, $CDCl_3$) of Compound **11**

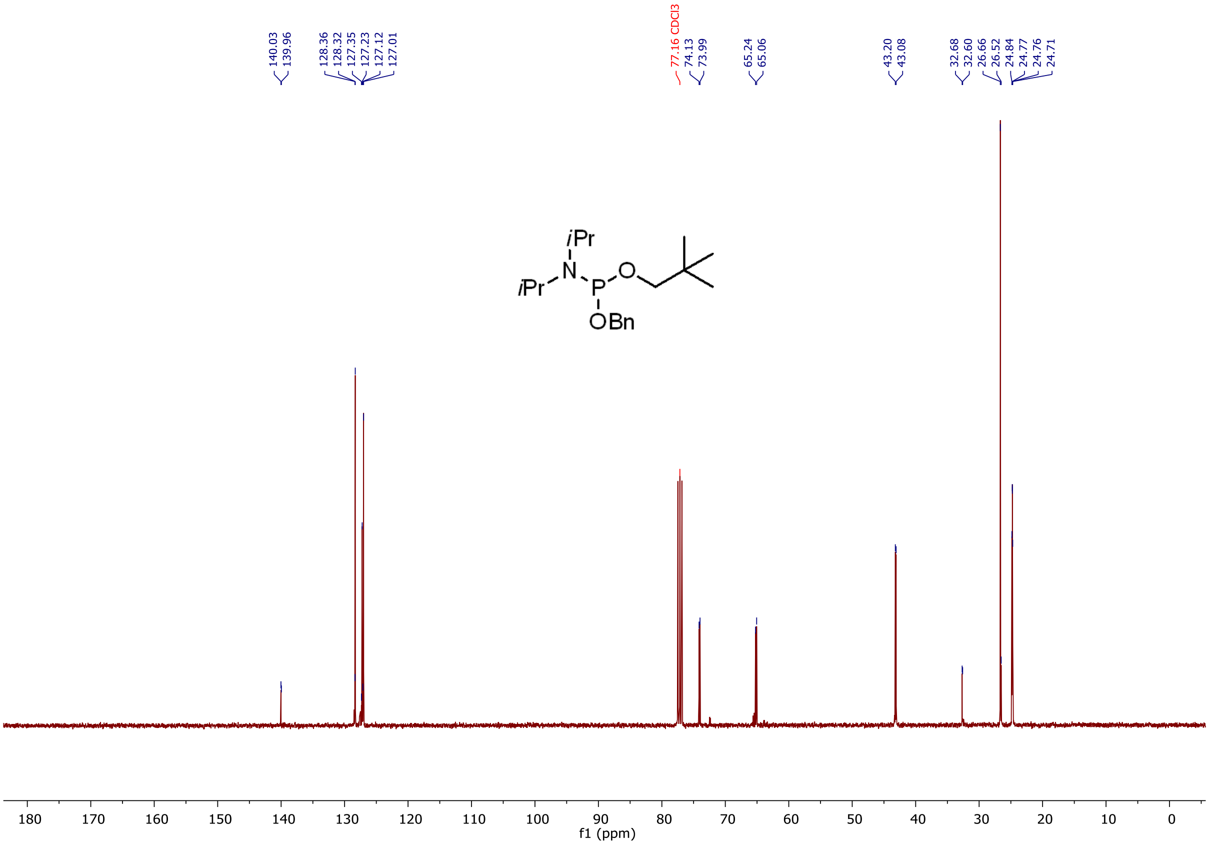

^{1}H NMR (400 MHz, $CDCl_3$) of Compound **10**

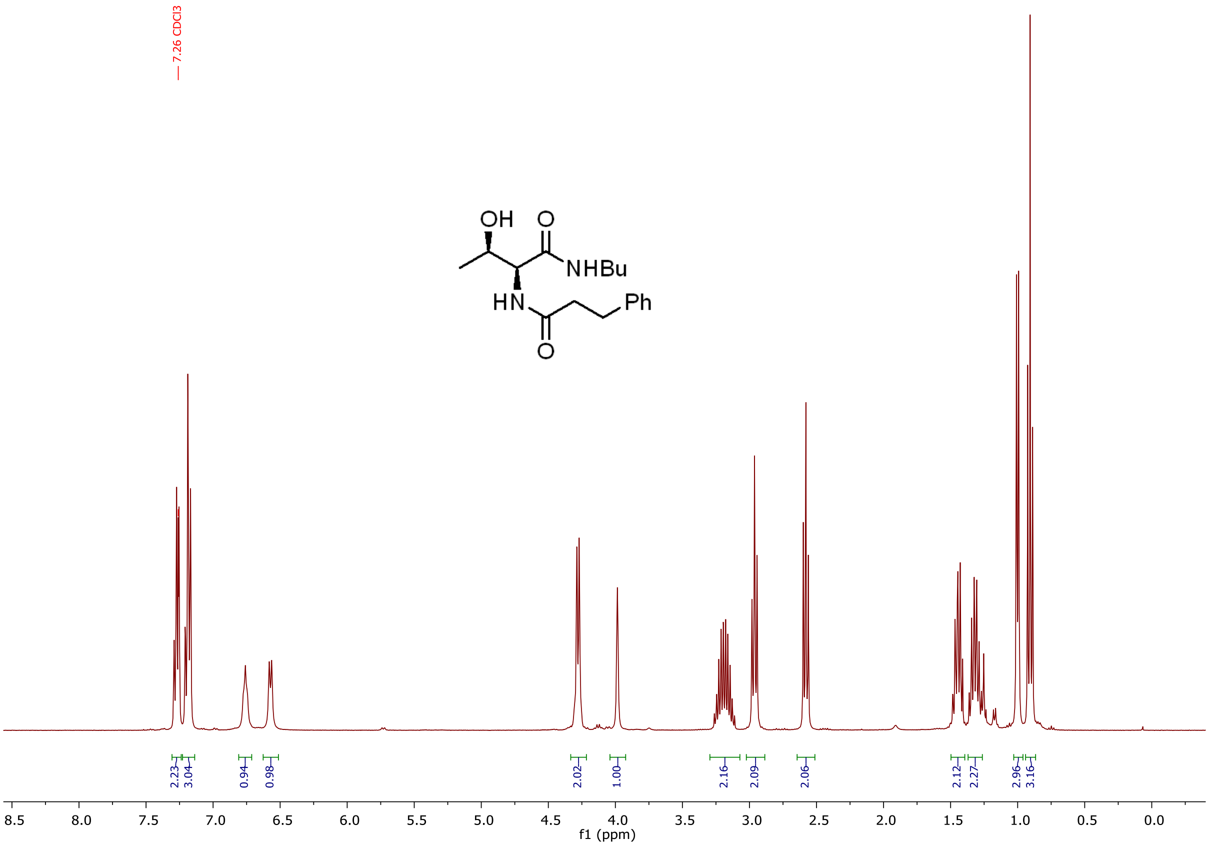

^{13}C NMR (101 MHz, $CDCl_3$) of Compound **10**

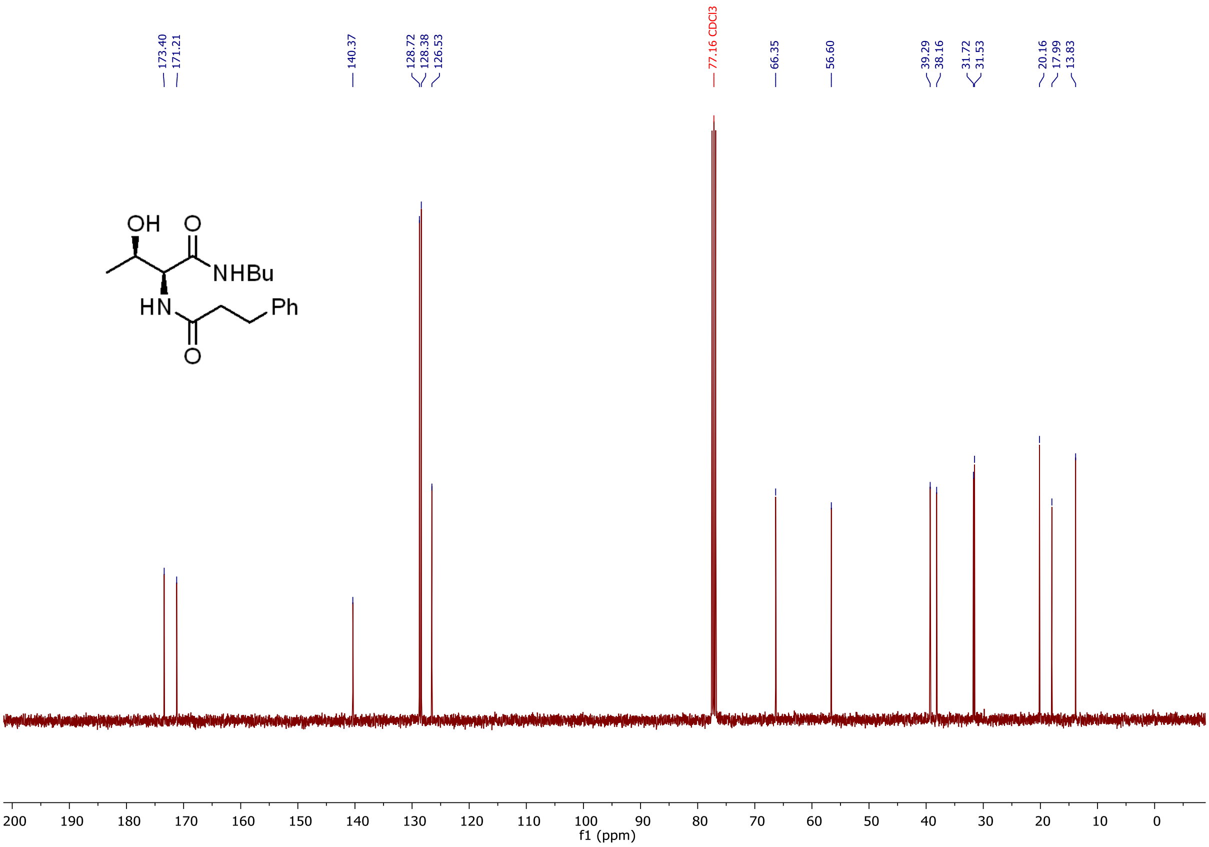

^{1}H NMR (400 MHz, $CDCl_3$) of Compound **12**

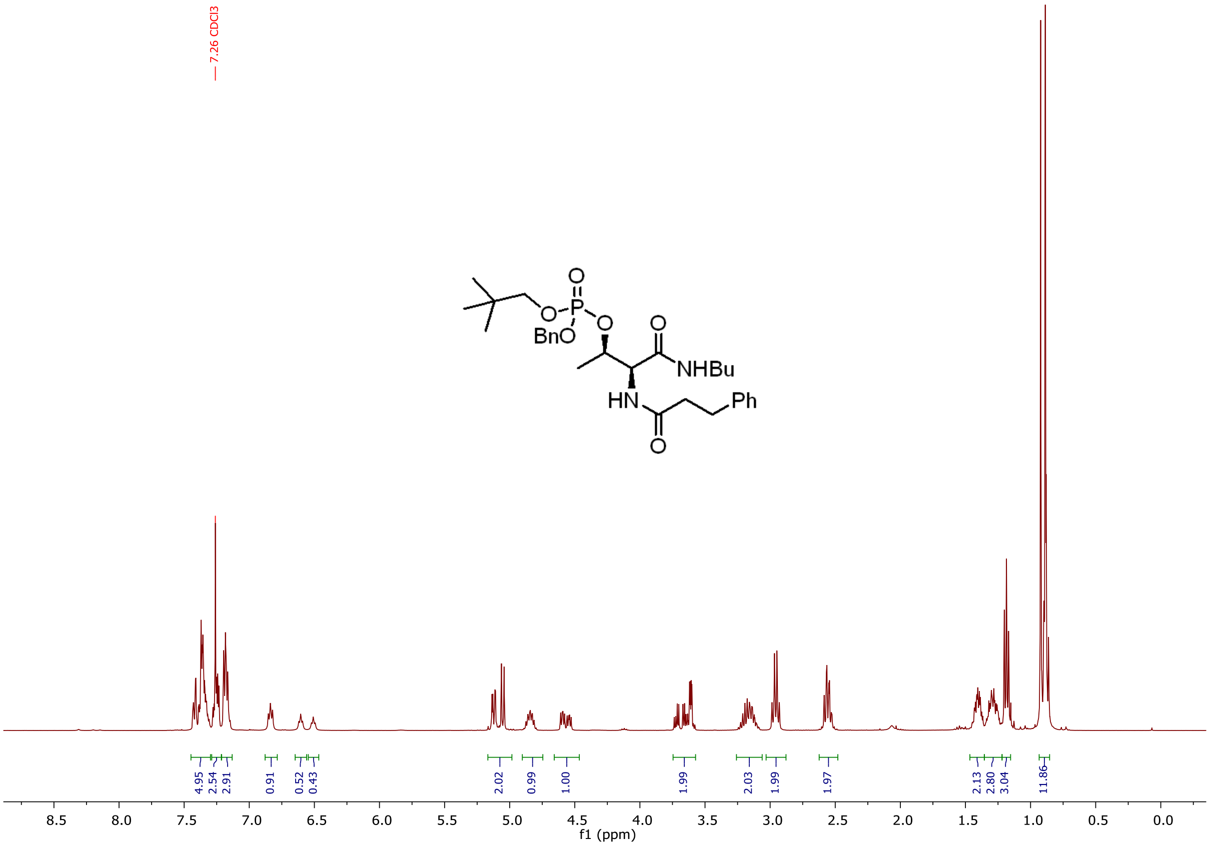

^{13}C NMR (101 MHz, $CDCl_3$) of Compound **12**

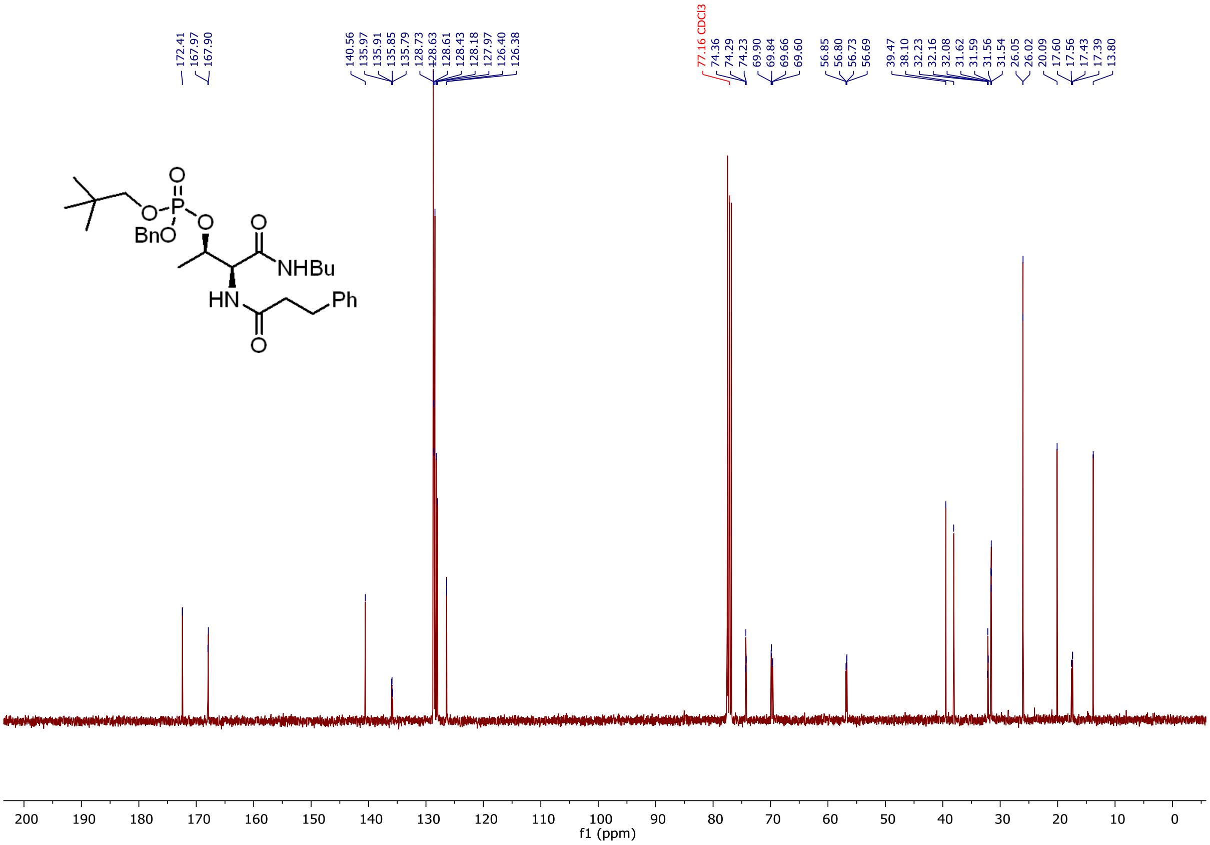

^{1}H NMR (400 MHz, $CDCl_3$) of Compound **2d**

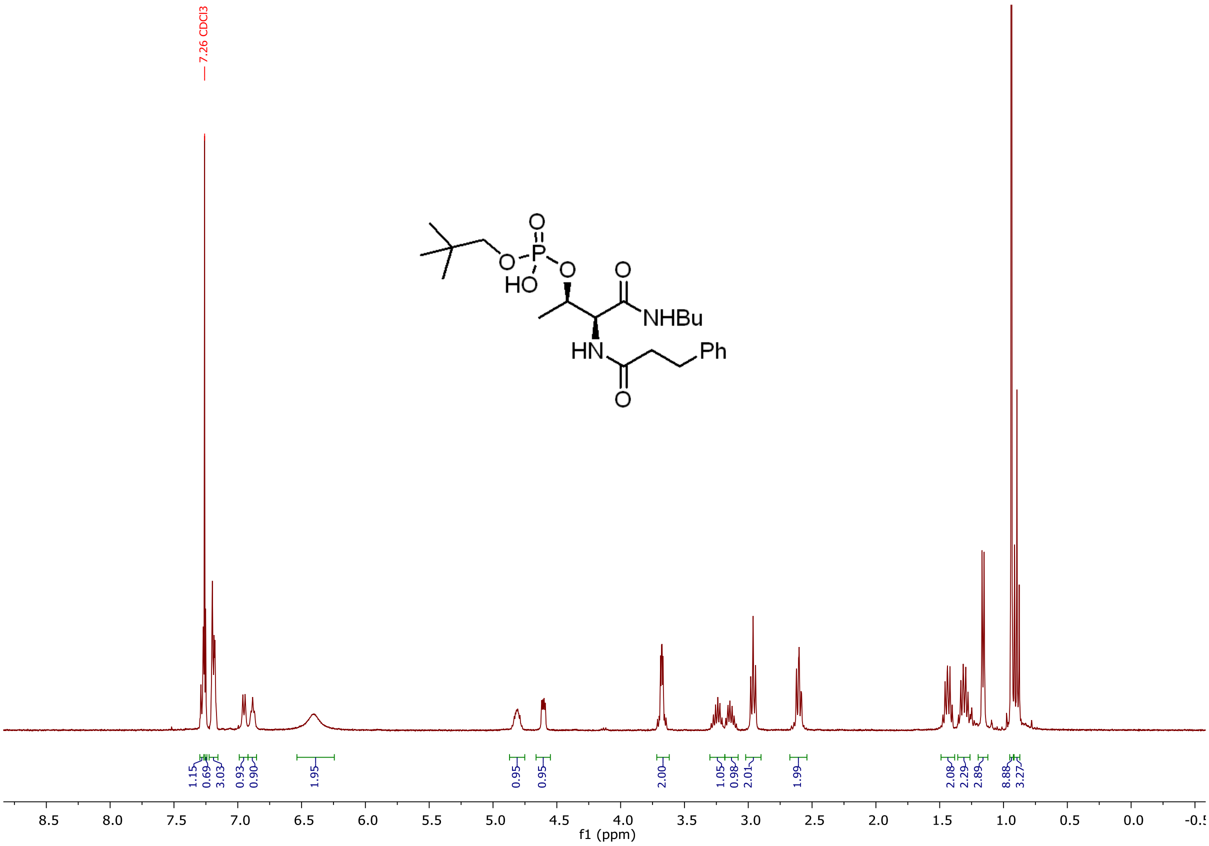

^{13}C NMR (125 MHz, $CDCl_3$) of Compound **2d**

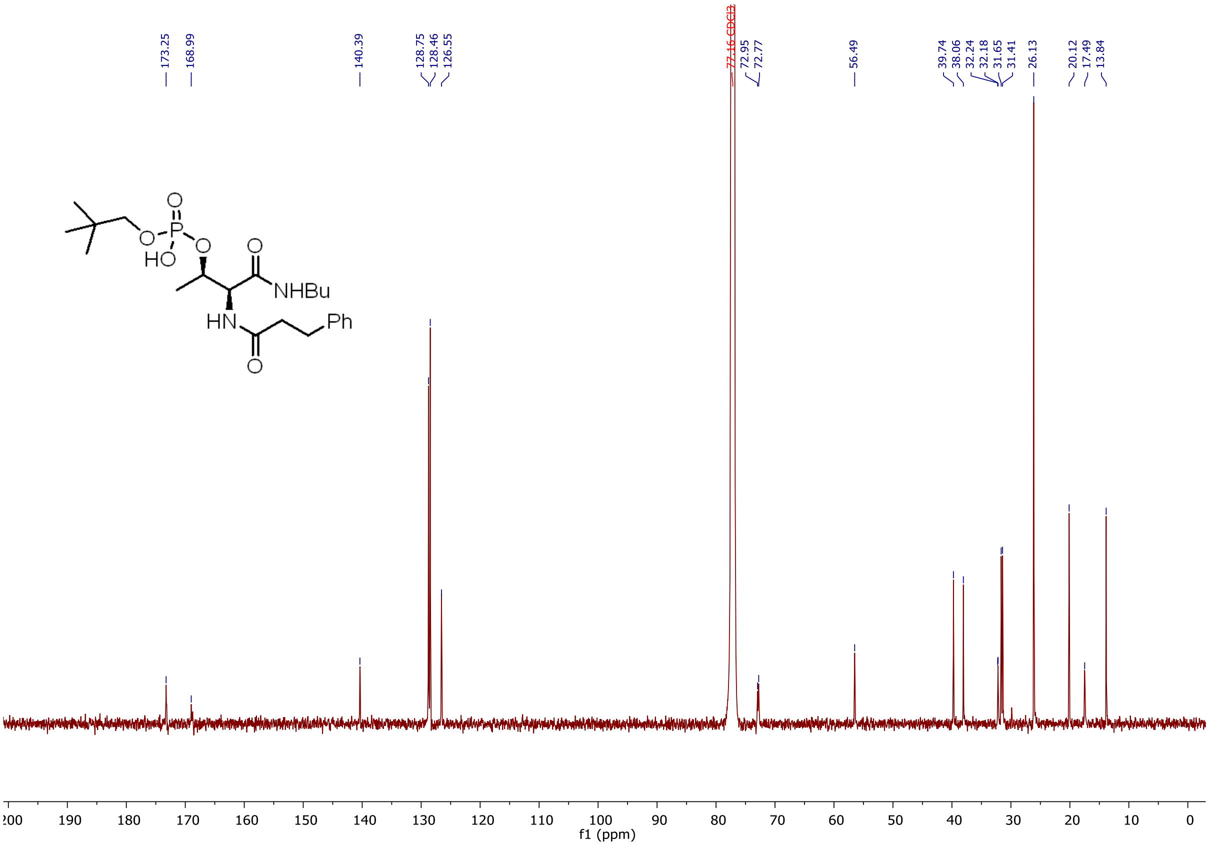

^{1}H NMR (400 MHz, $CDCl_3$) of Compound **13**

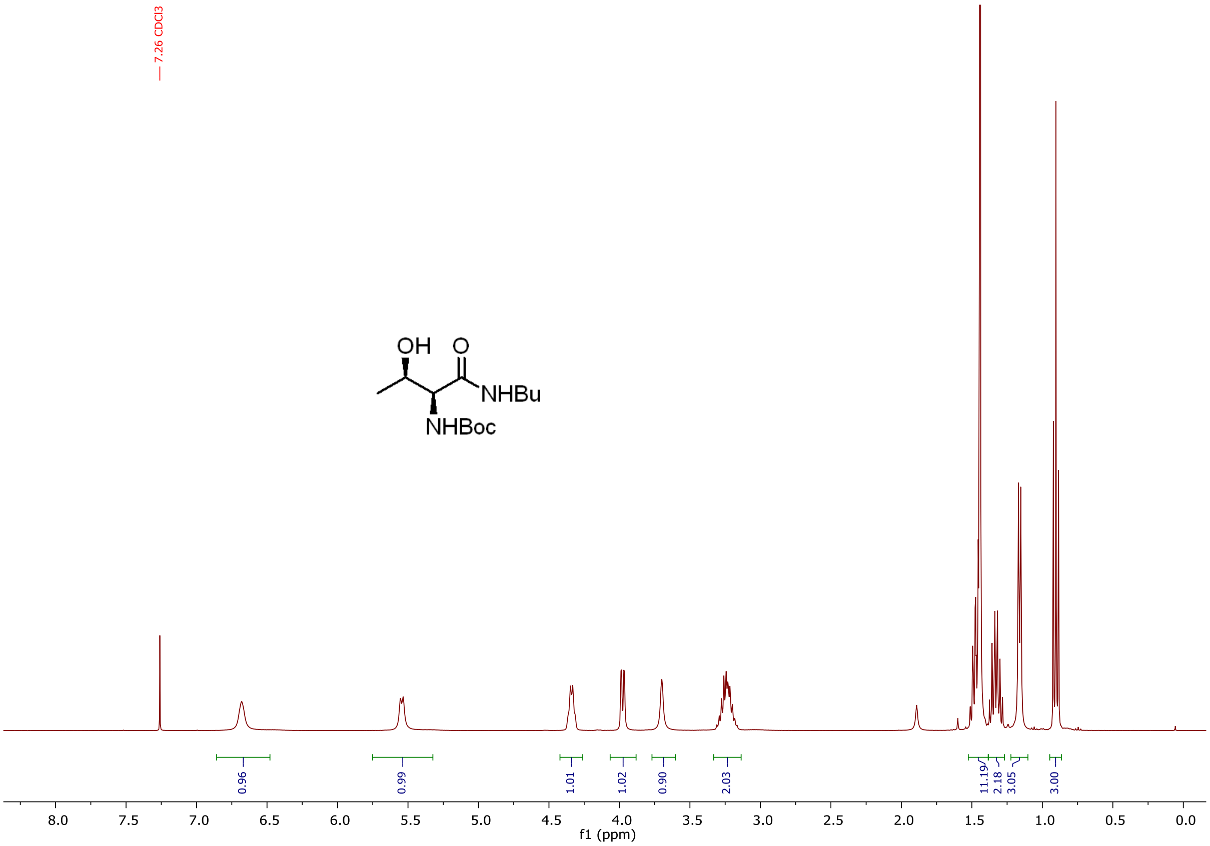

^{13}C NMR (101 MHz, $CDCl_3$) of Compound **13**

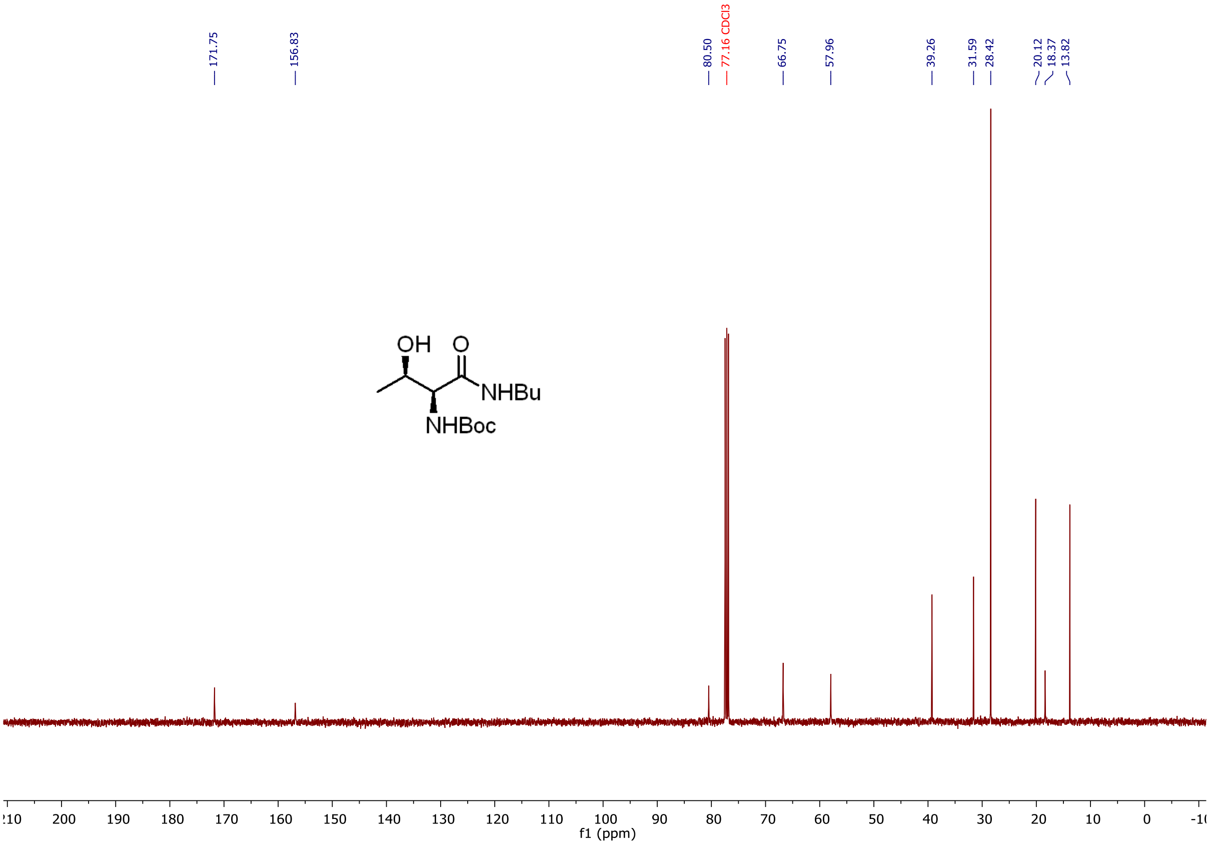

^{1}H NMR (400 MHz, $CDCl_3$) of Compound **14**

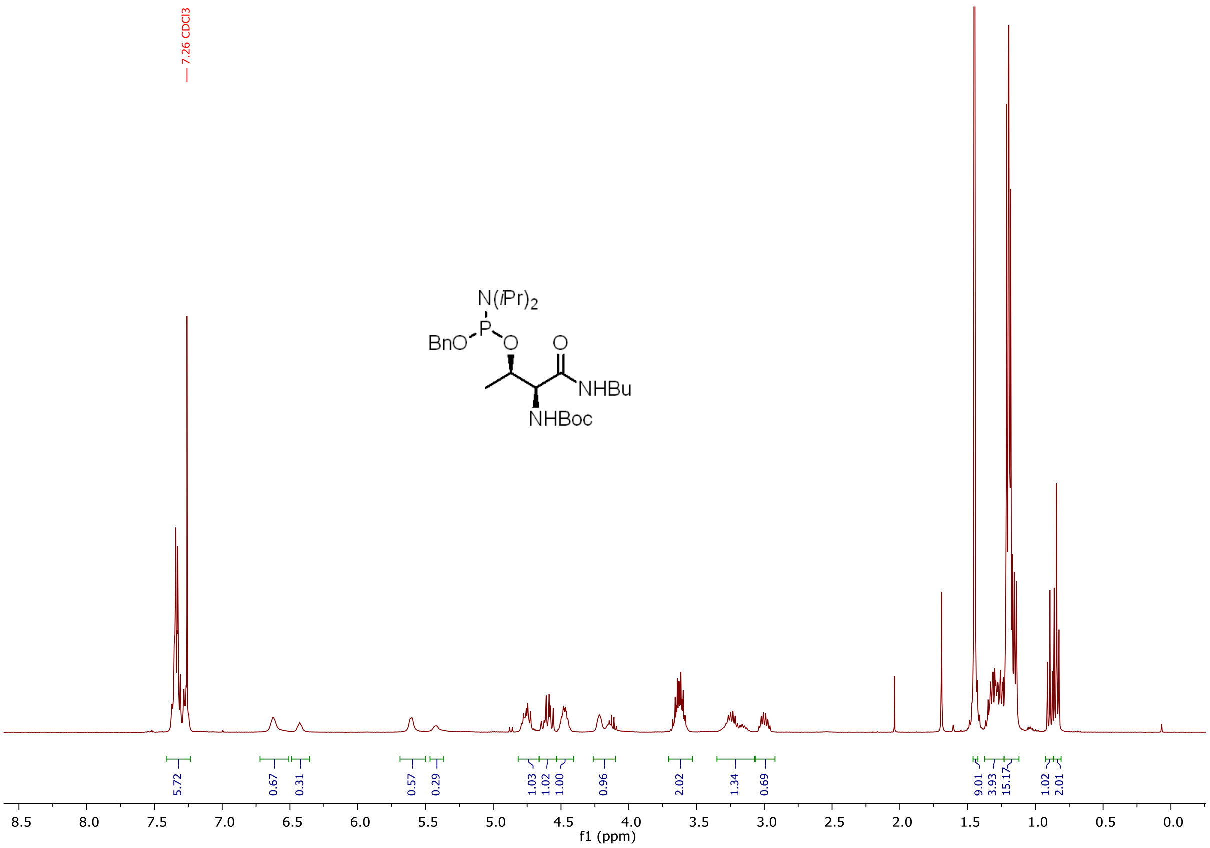

^{13}C NMR (125 MHz, $CDCl_3$) of Compound **14**

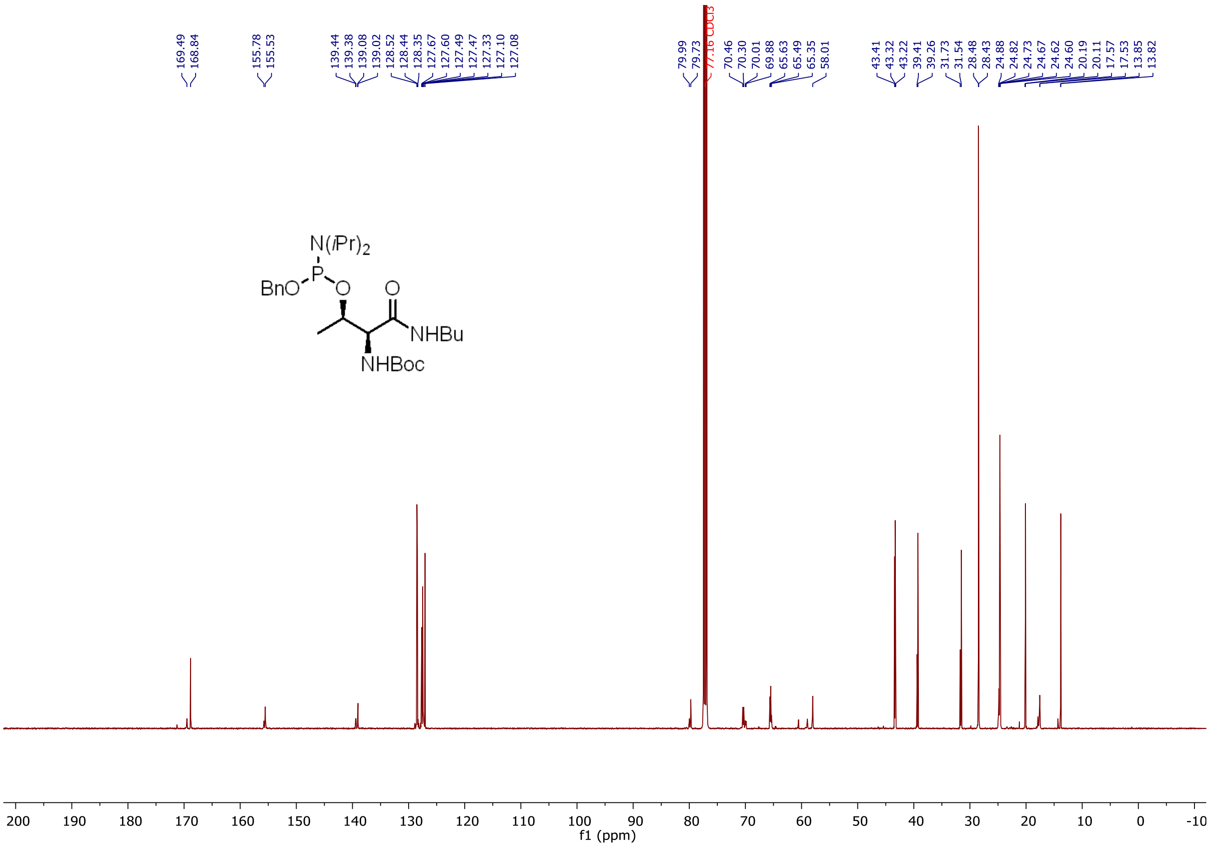

^{1}H NMR (400 MHz, CDCl$_3$) of Compound **15a**

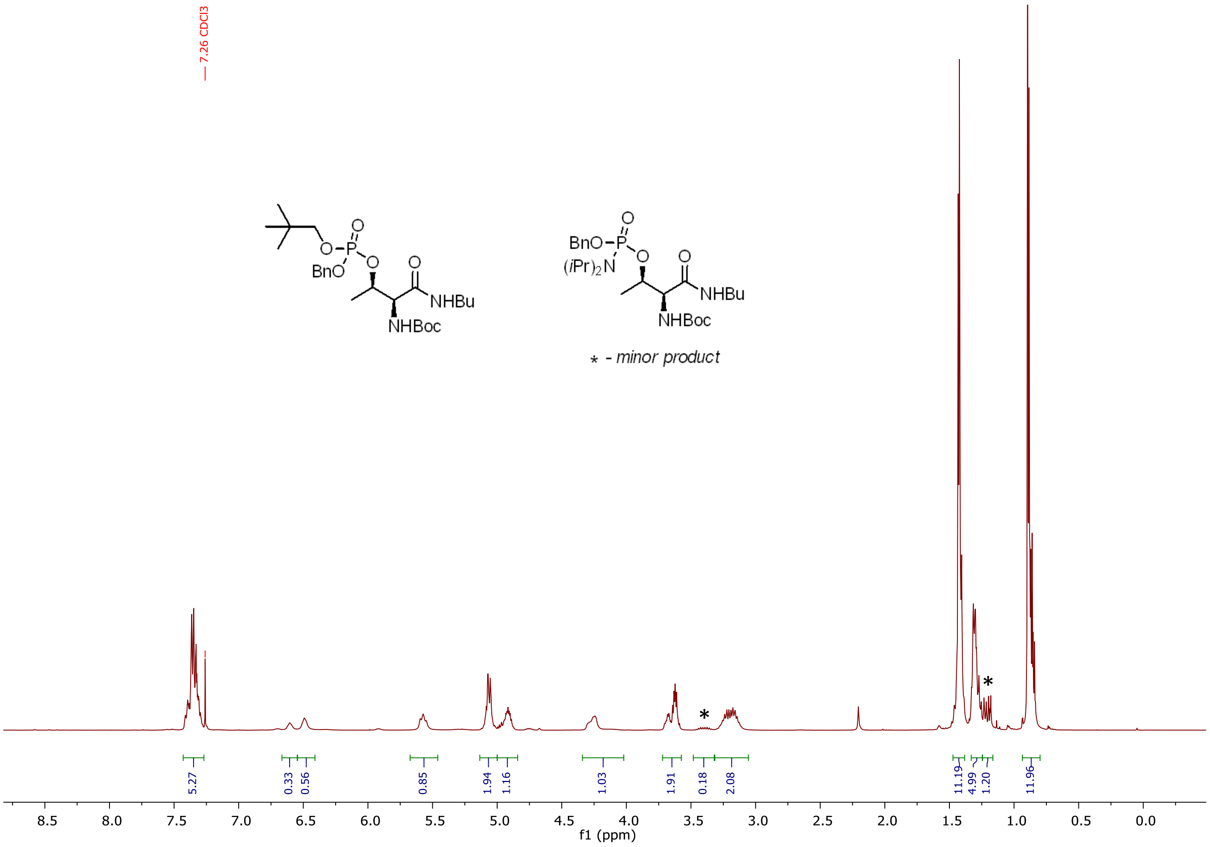

^{13}C NMR (101 MHz, CDCl$_3$) of Compound **15a**

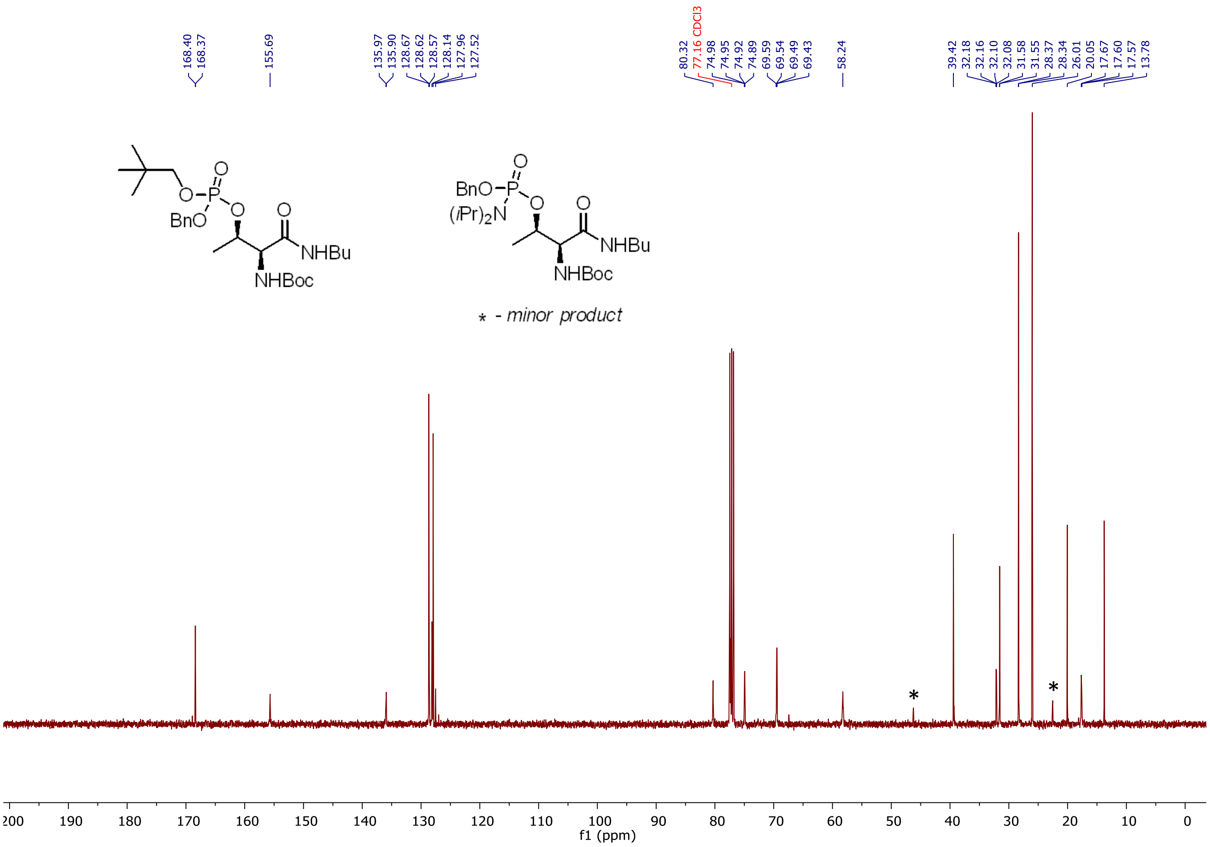

^{1}H NMR (400 MHz, $CDCl_3$) of Compound **16**

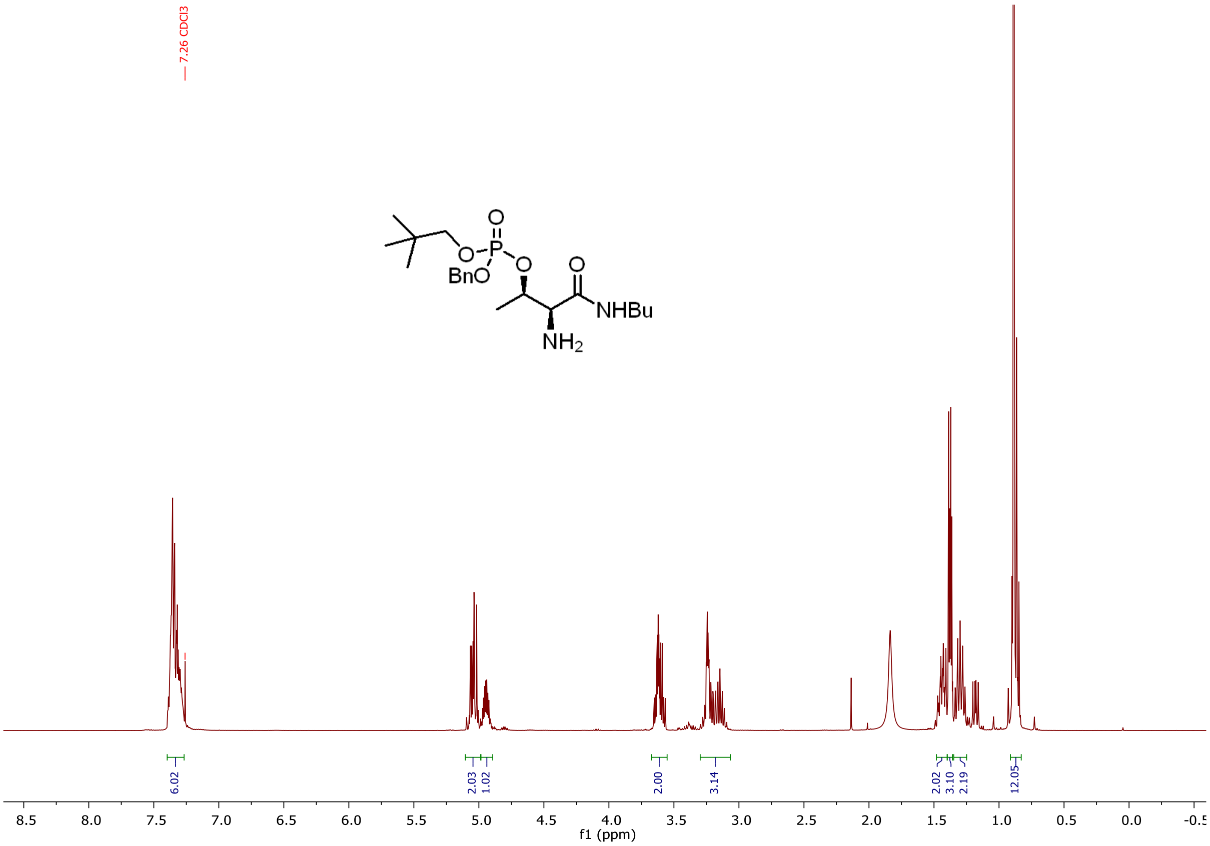

^{13}C NMR (101 MHz, $CDCl_3$) of Compound **16**

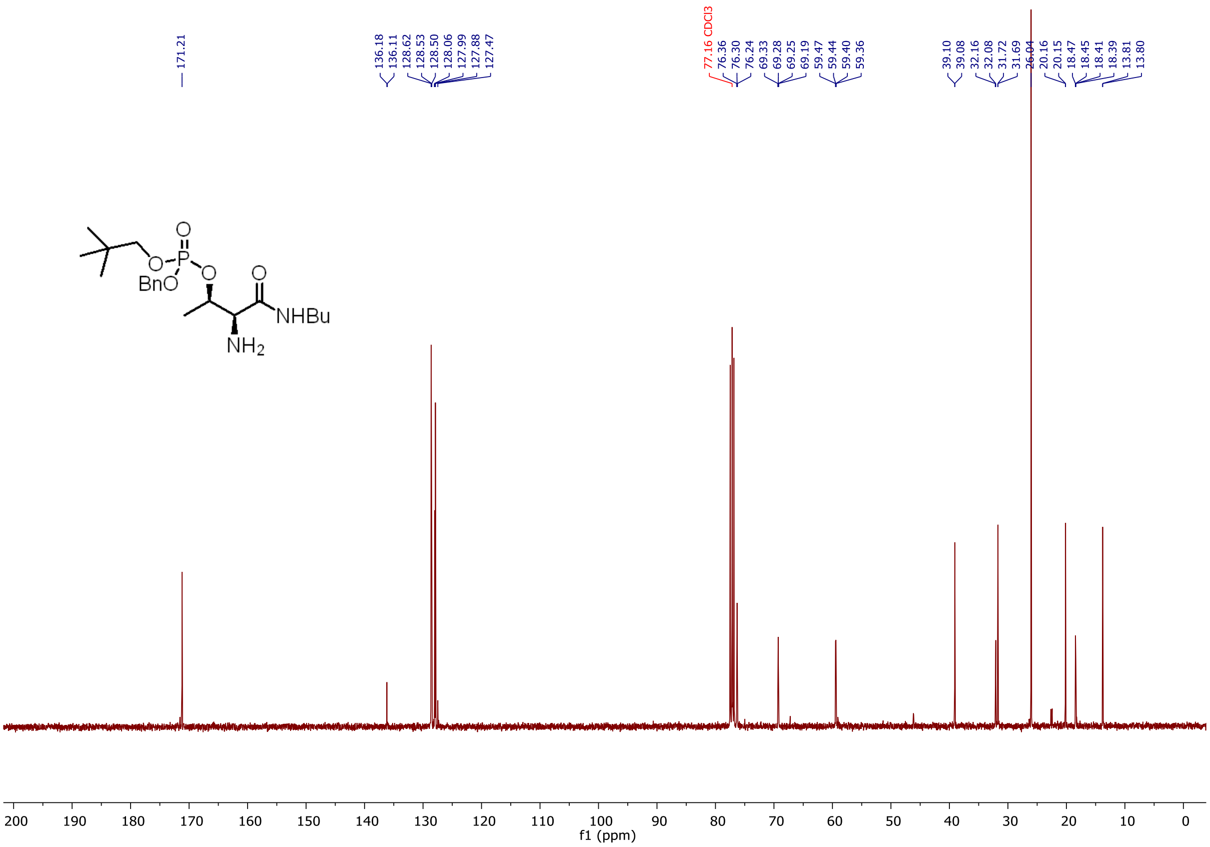

^{1}H NMR (400 MHz, $CDCl_3$) of Compound **17**

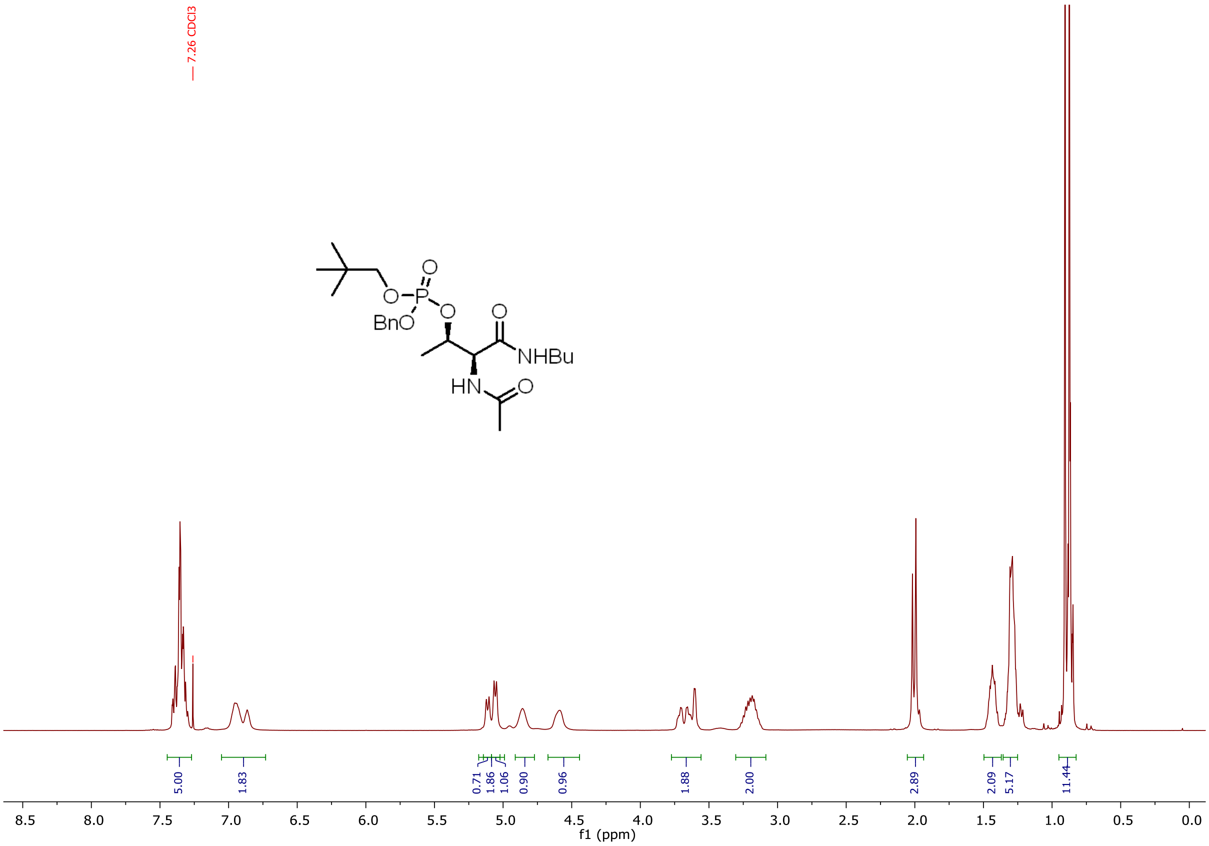

^{13}C NMR (125 MHz, $CDCl_3$) of Compound **17**

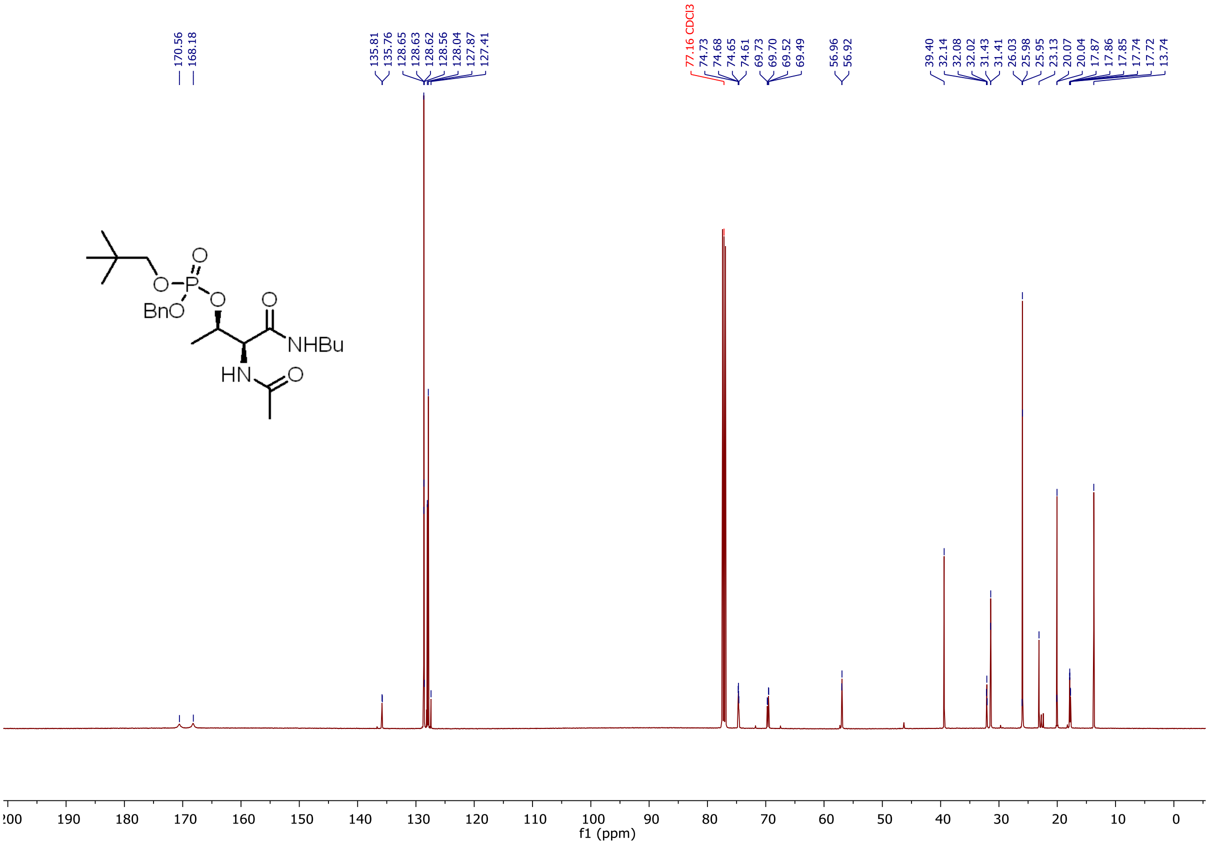

^{1}H NMR (400 MHz, $CDCl_3$) of Compound **18**

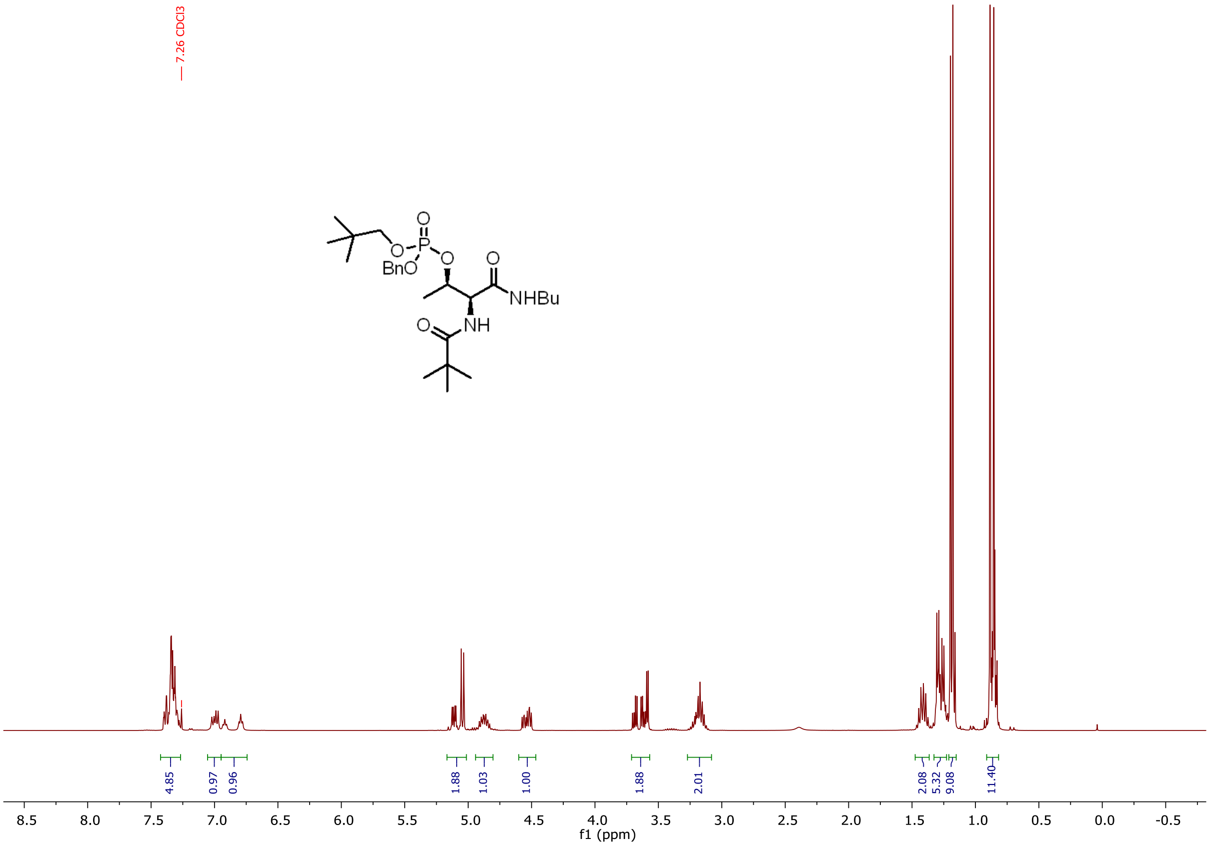

^{13}C NMR (101 MHz, $CDCl_3$) of Compound **18**

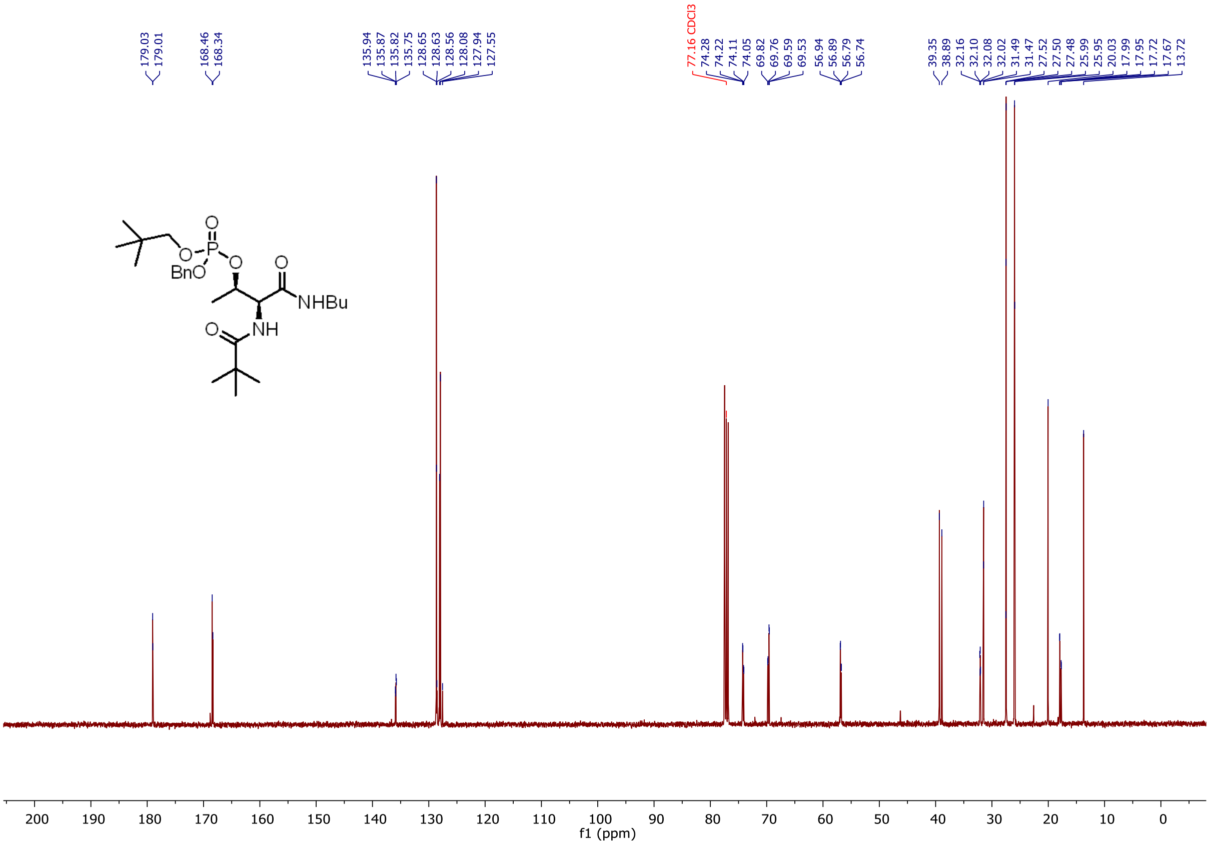

^{1}H NMR (400 MHz, $CDCl_3$) of Compound **2e**

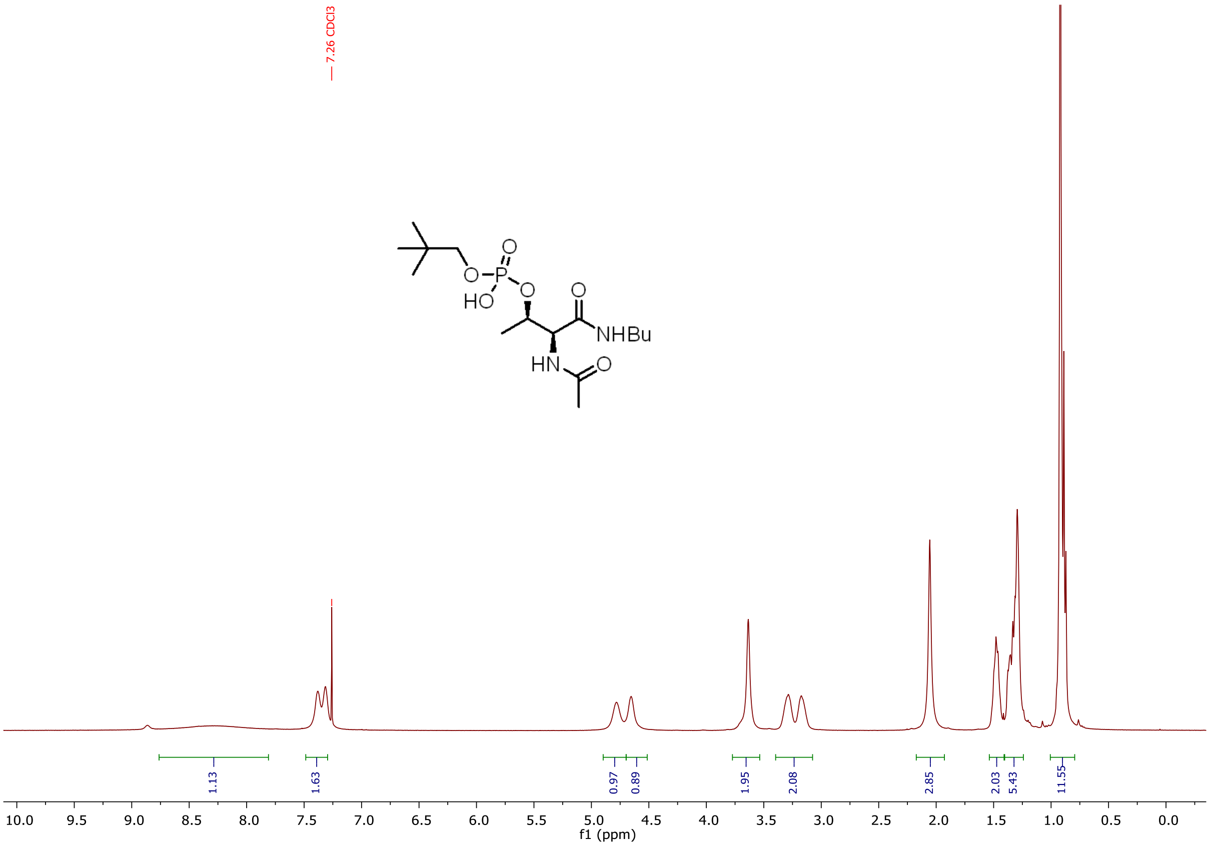

^{13}C NMR (101 MHz, $CDCl_3$) of Compound **2e**

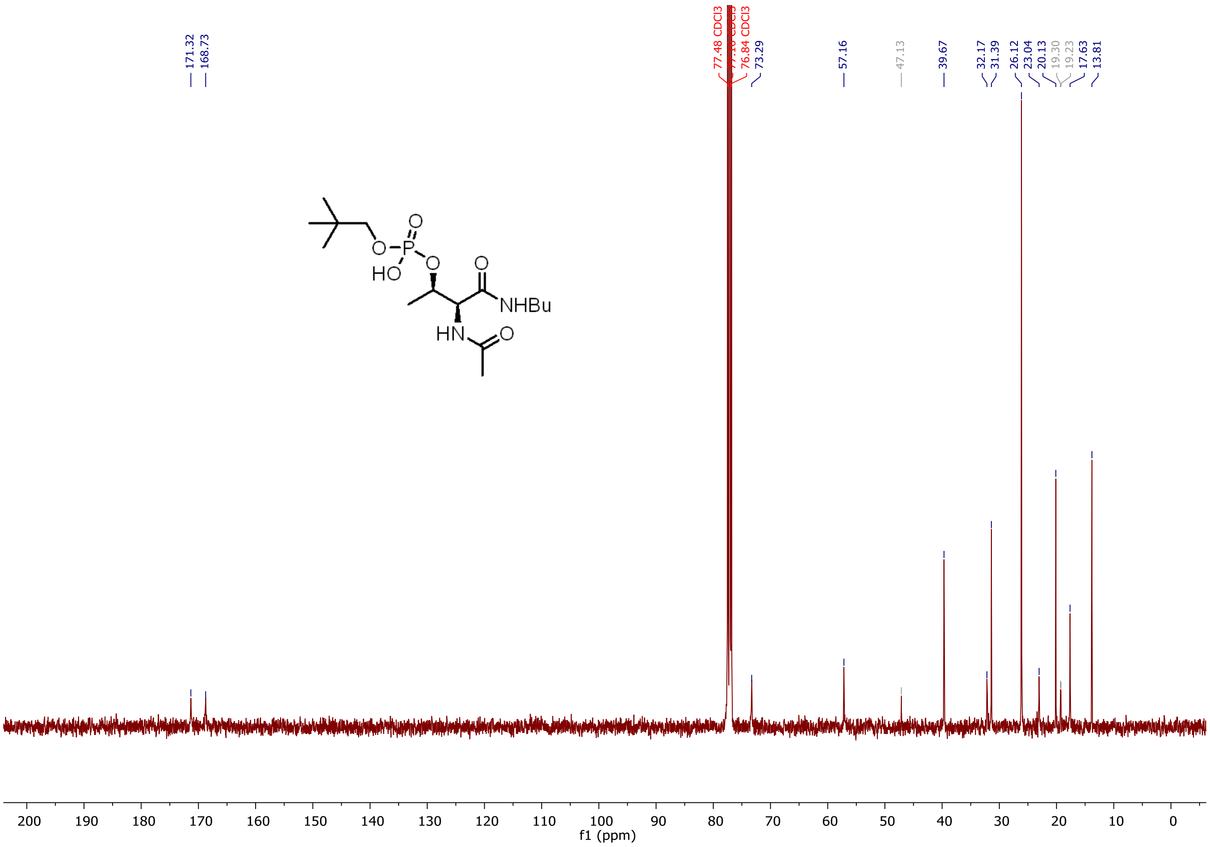

^{1}H NMR (400 MHz, $CDCl_3$) of Compound **2f**

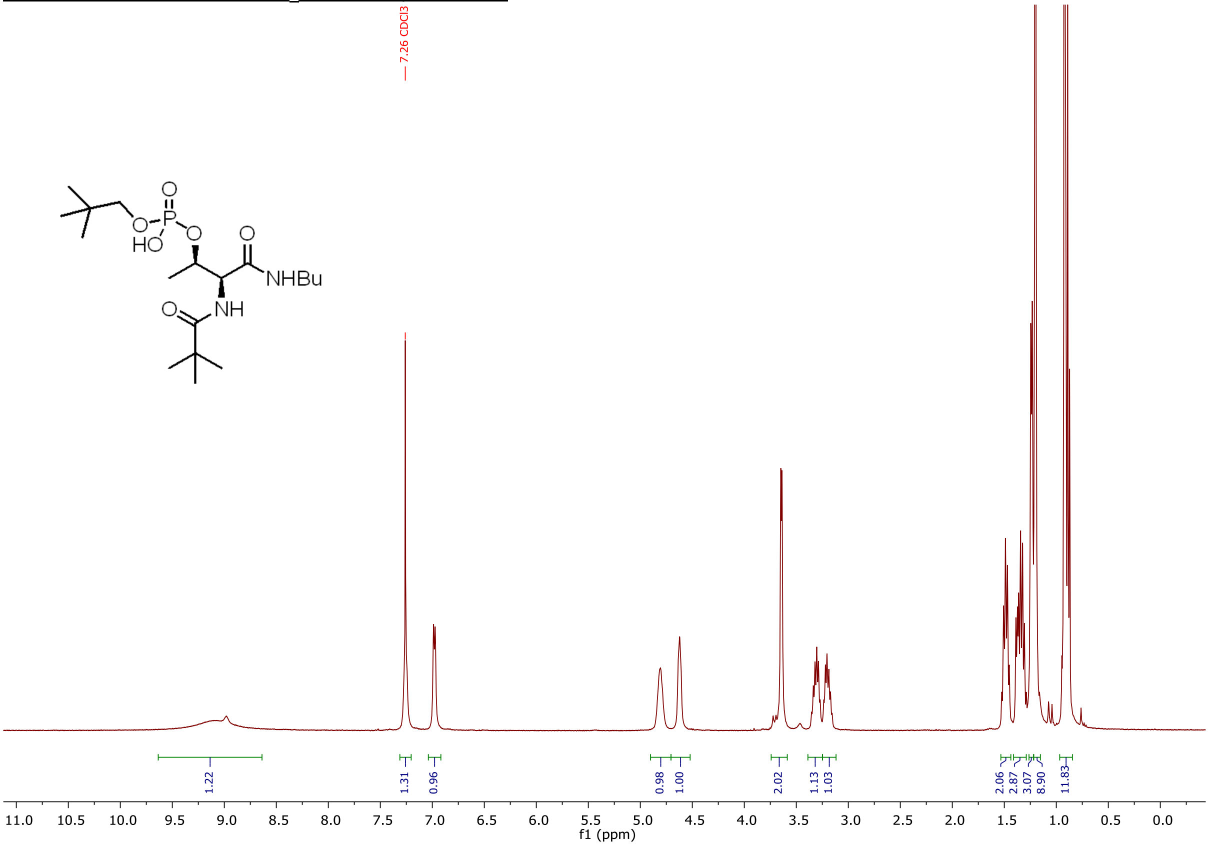

^{13}C NMR (101 MHz, $CDCl_3$) of Compound **2f**

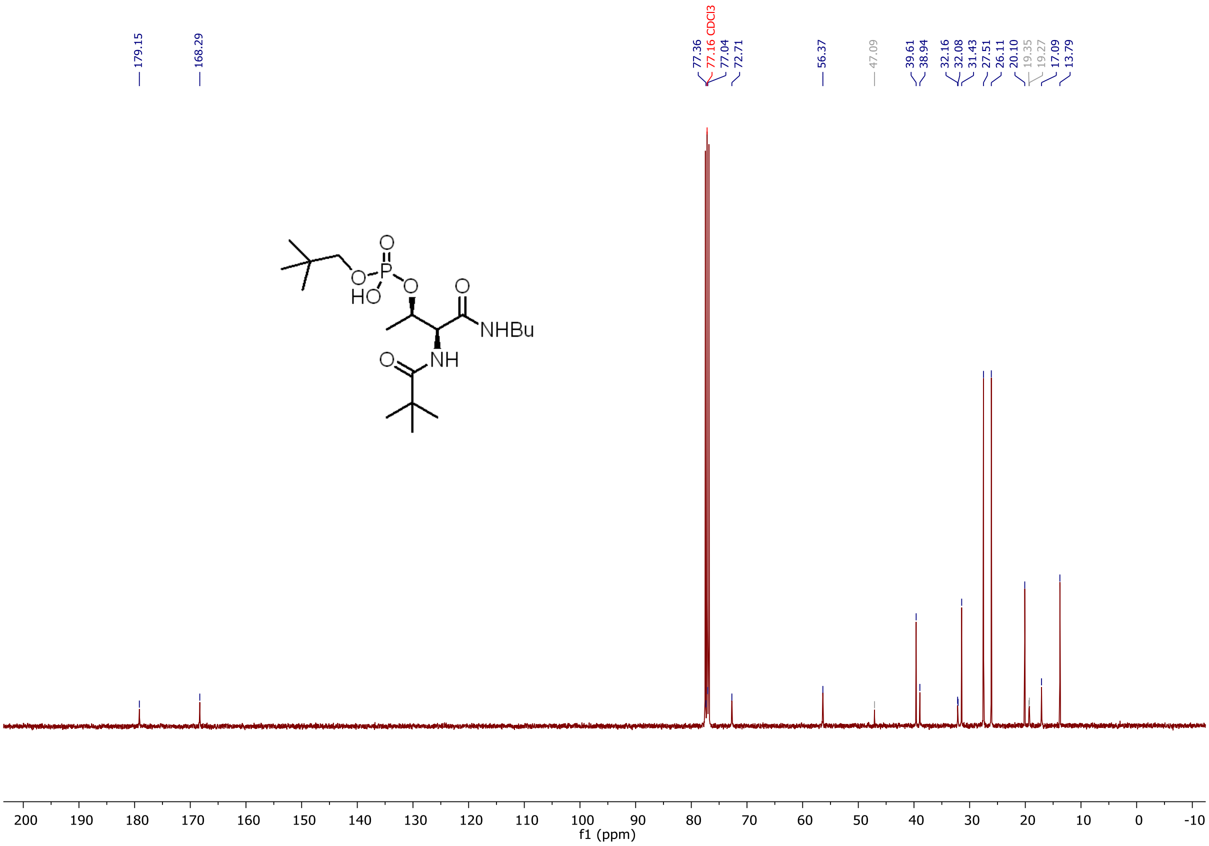

^{1}H NMR (400 MHz, $CDCl_3$) of Compound **19**

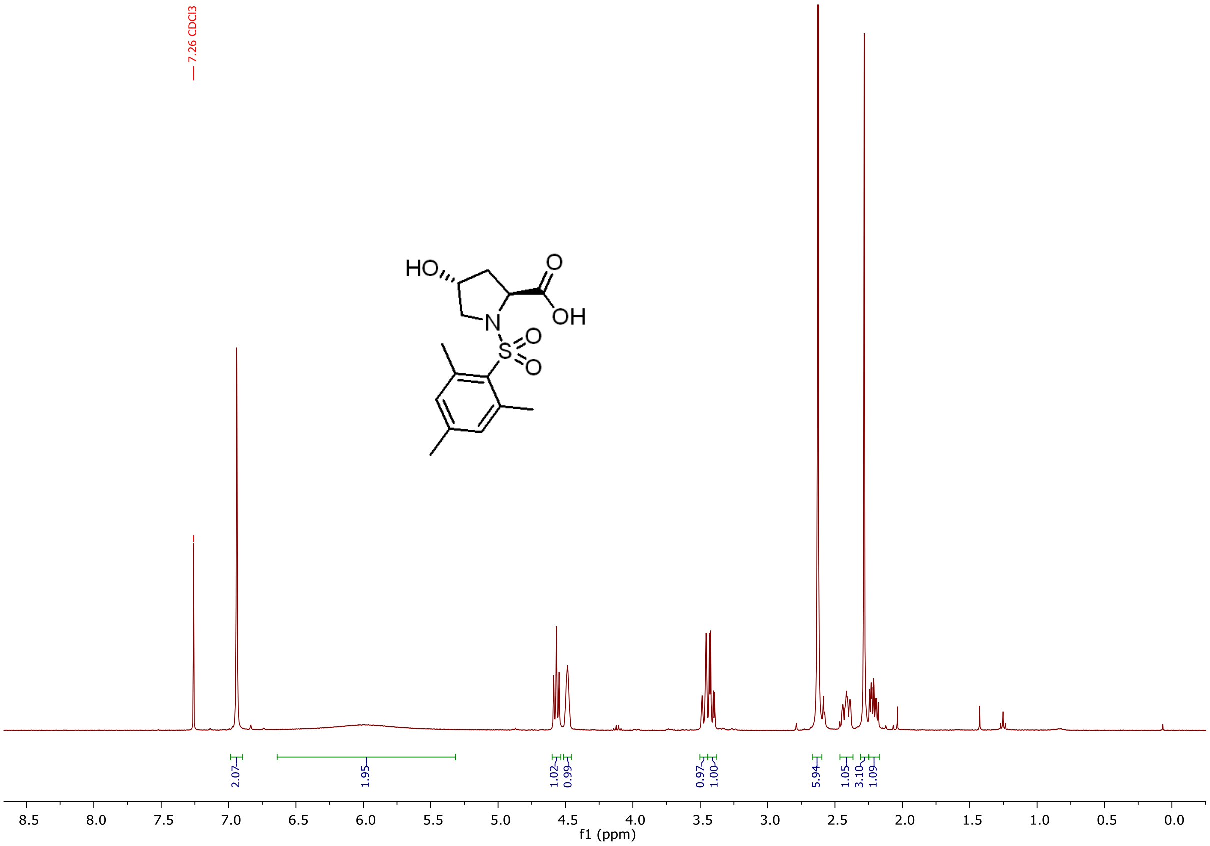

^{13}C NMR (101 MHz, $CDCl_3$) of Compound **19**

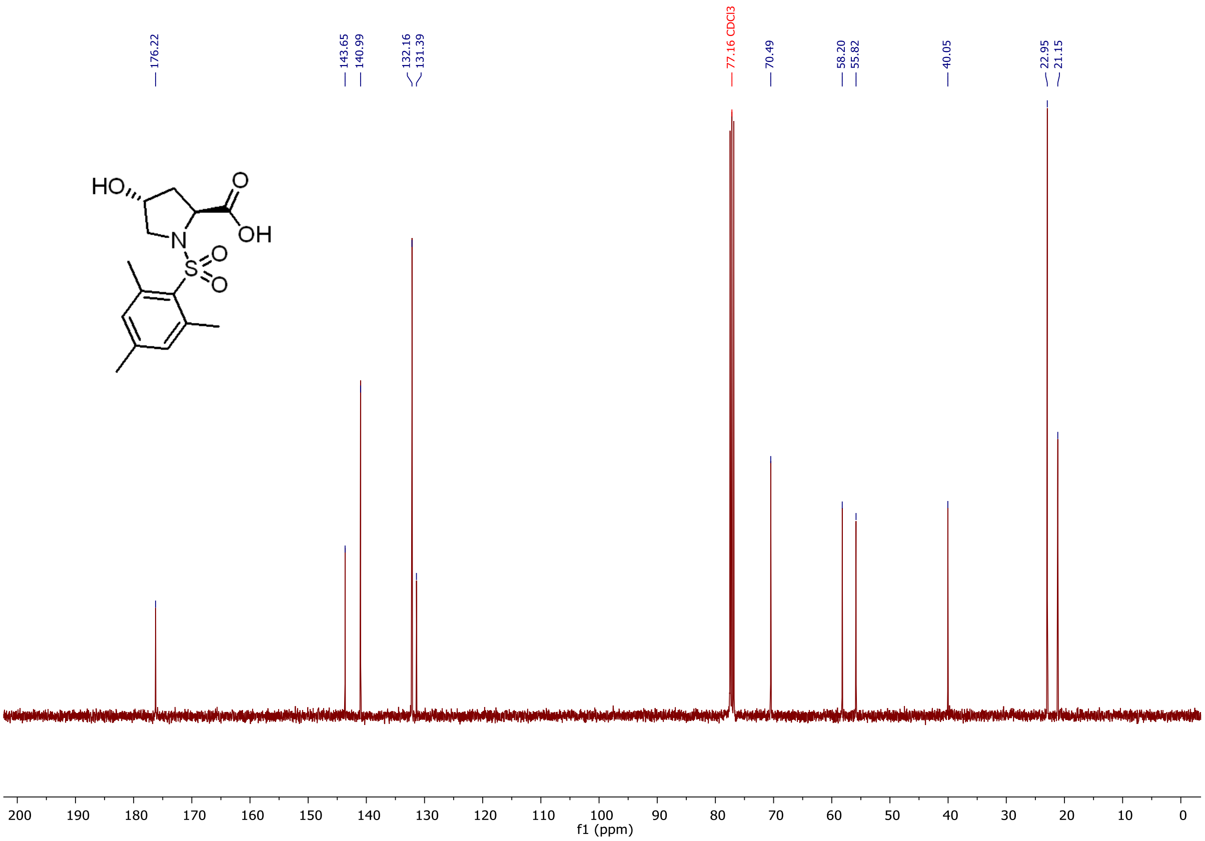

^{1}H NMR (400 MHz, $CDCl_3$) of Compound **20**

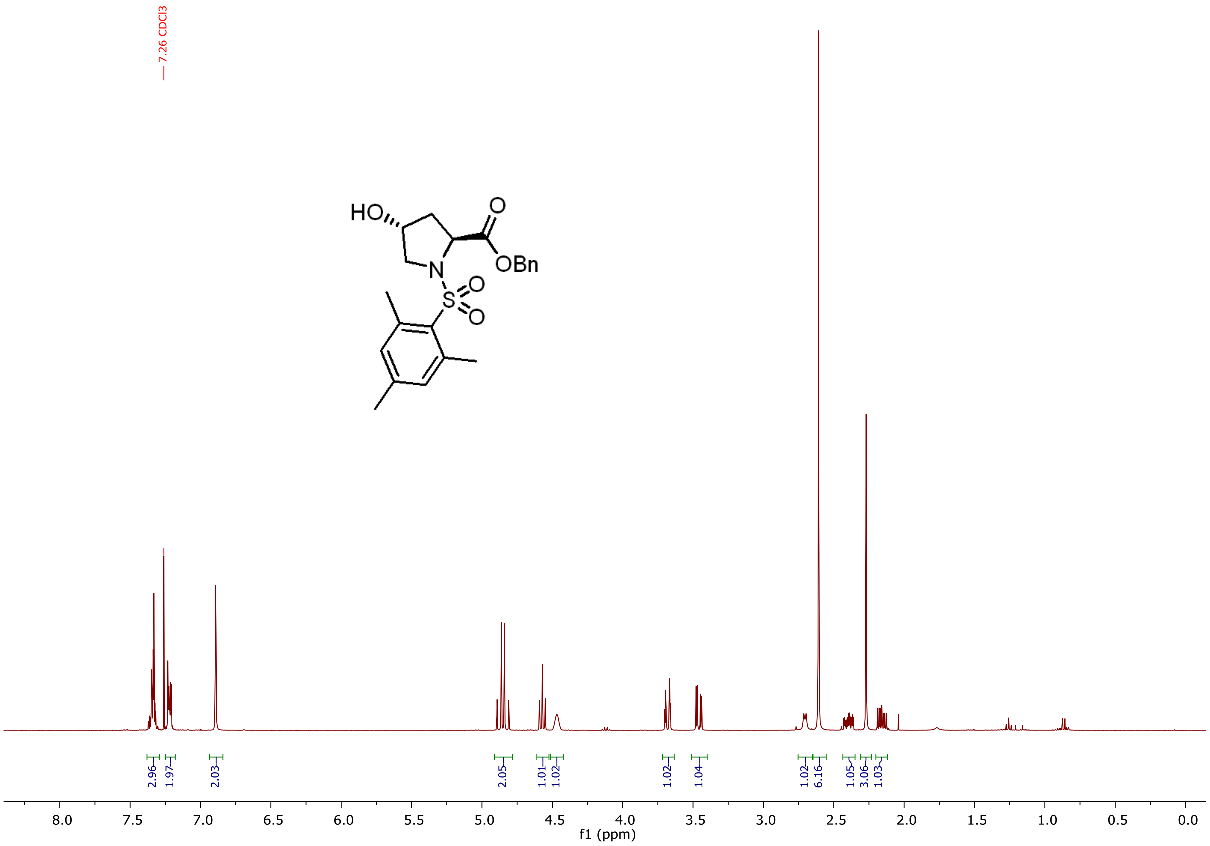

^{13}C NMR (101 MHz, $CDCl_3$) of Compound **20**

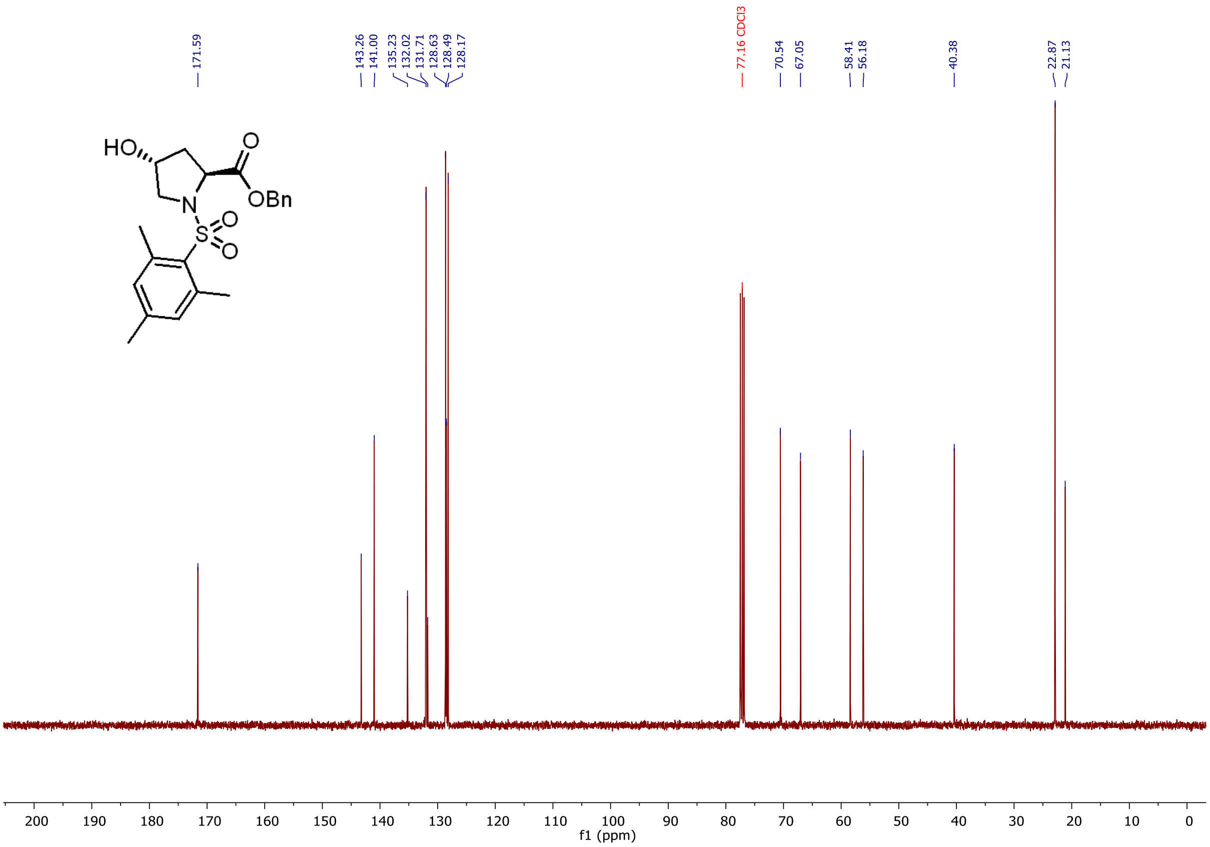

^{1}H NMR (400 MHz, $CDCl_3$) of Compound **21**

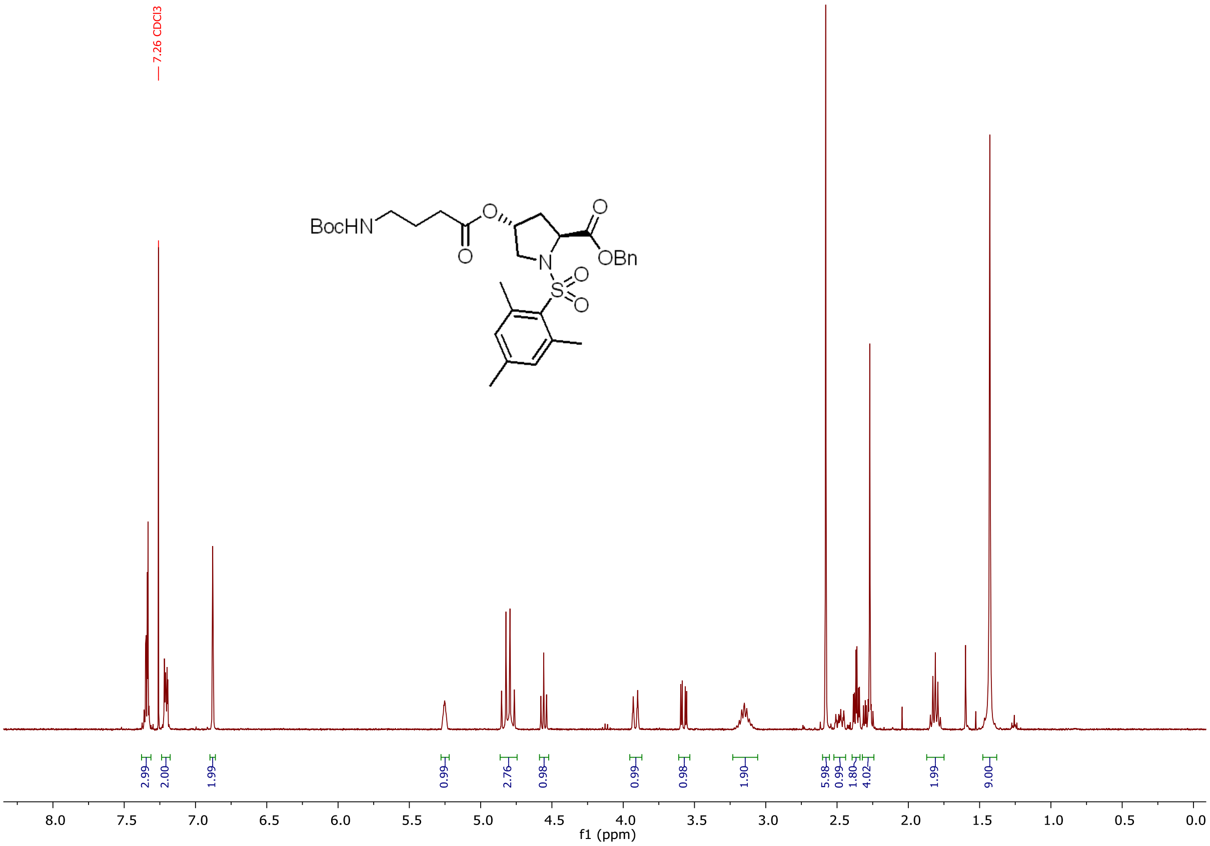

^{13}C NMR (101 MHz, $CDCl_3$) of Compound **21**

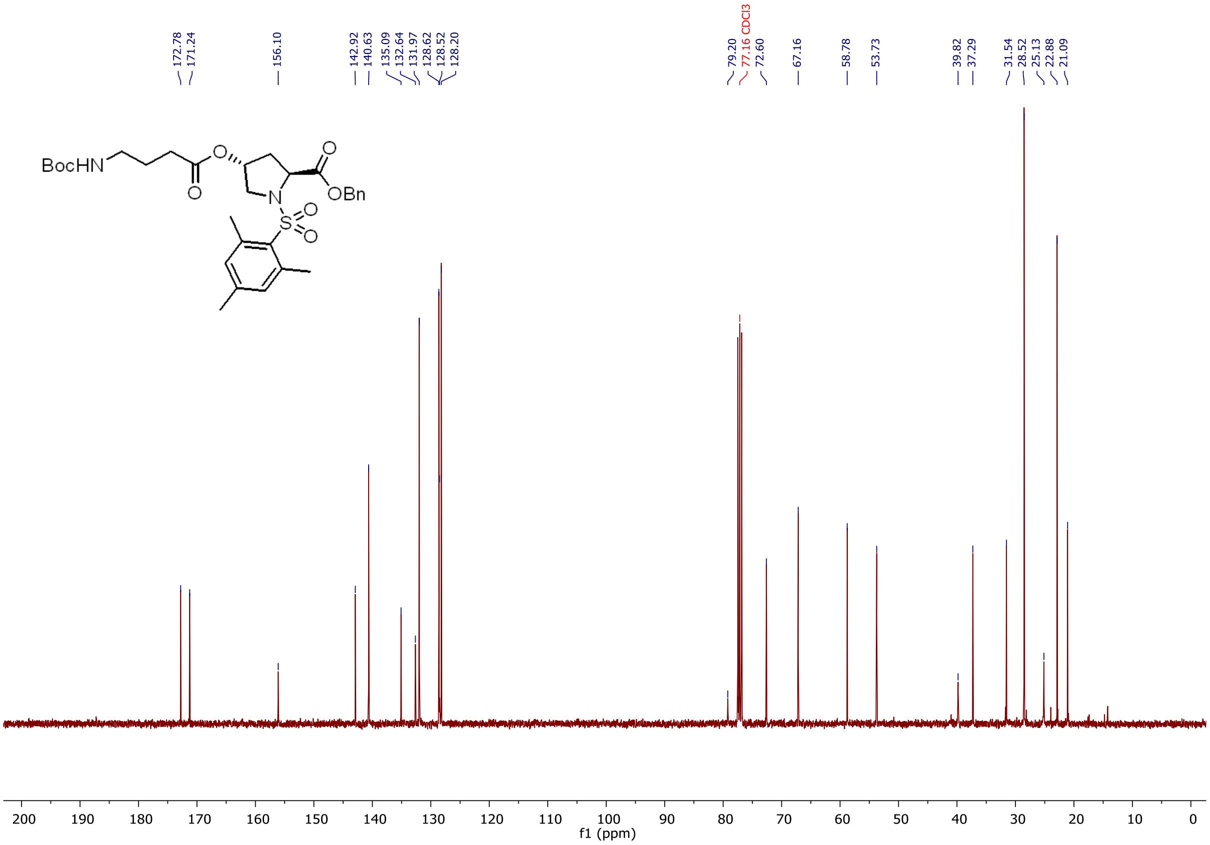

^{1}H NMR (400 MHz, $CDCl_3$) of Compound **3**

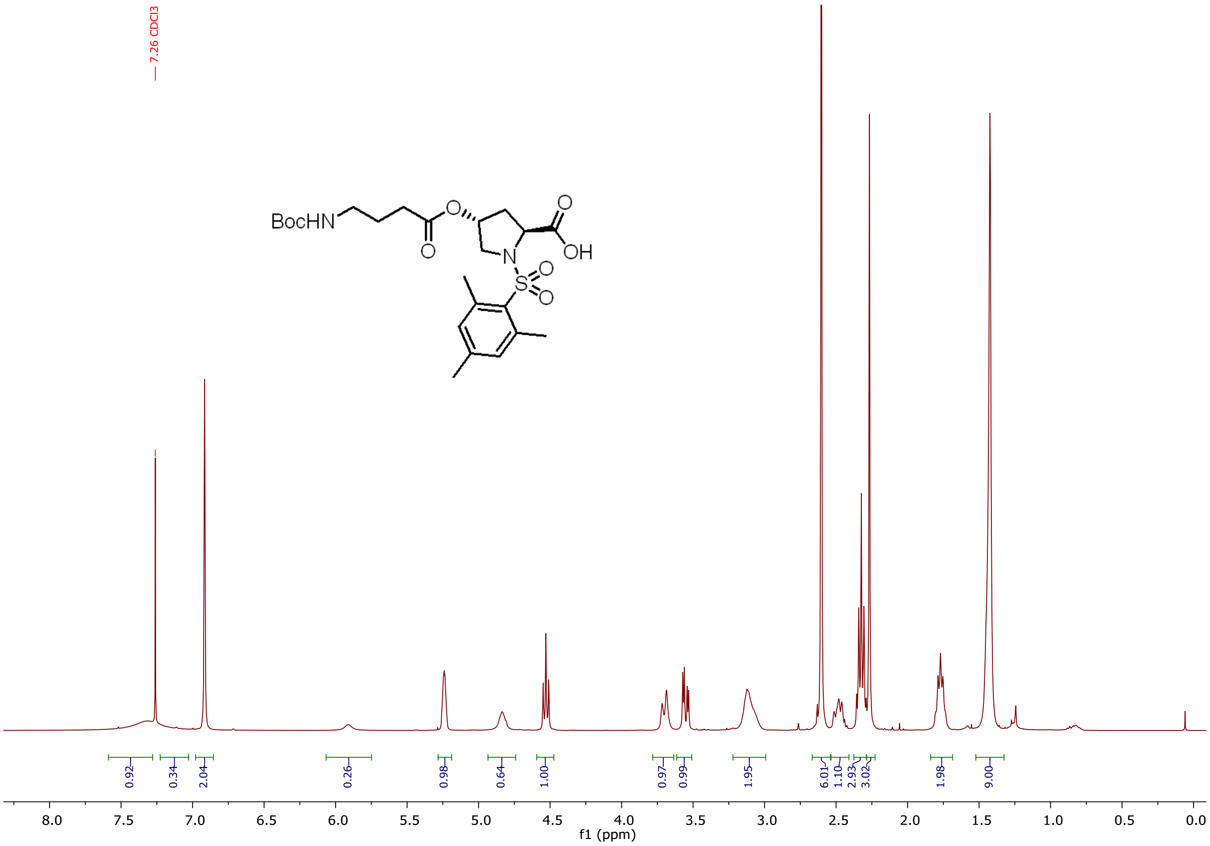

^{13}C NMR (101 MHz, $CDCl_3$) of Compound **3**

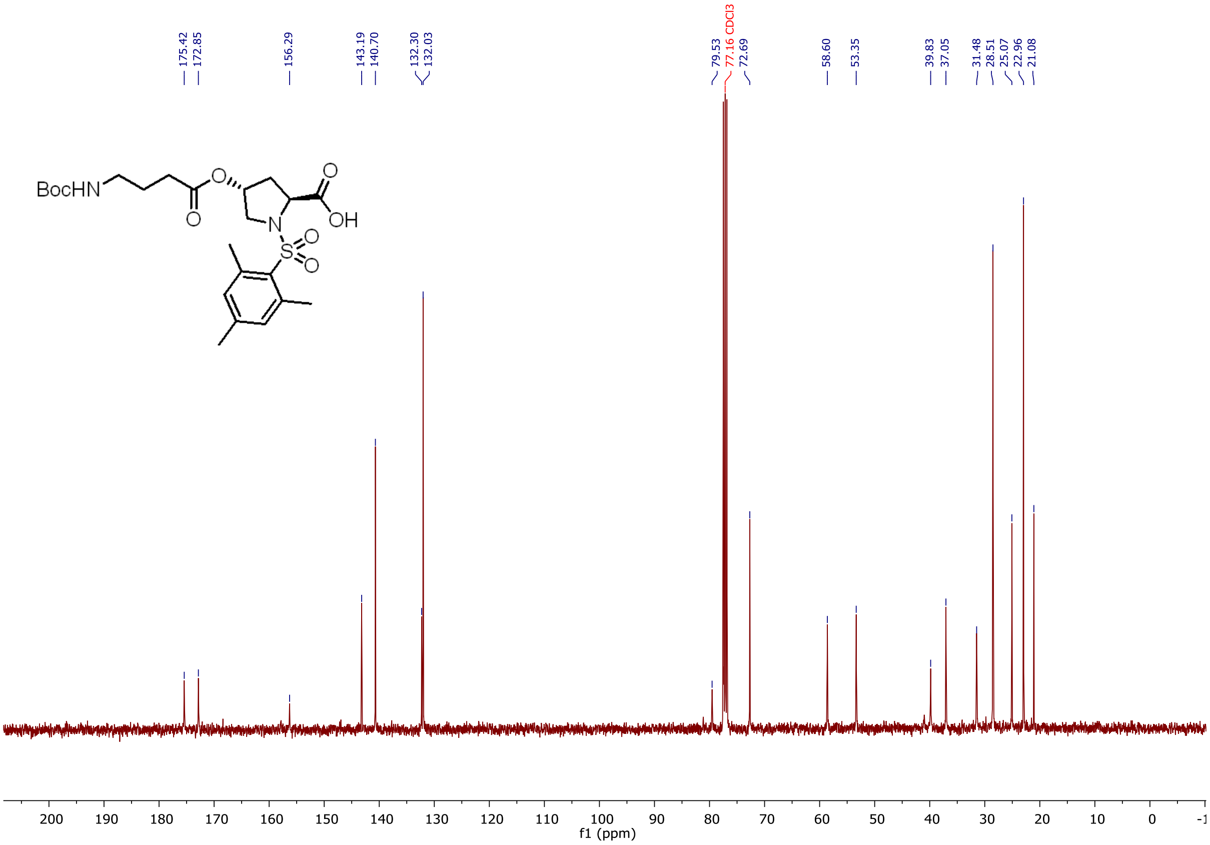

^{1}H NMR (400 MHz, $CDCl_3$) of Compound **22**

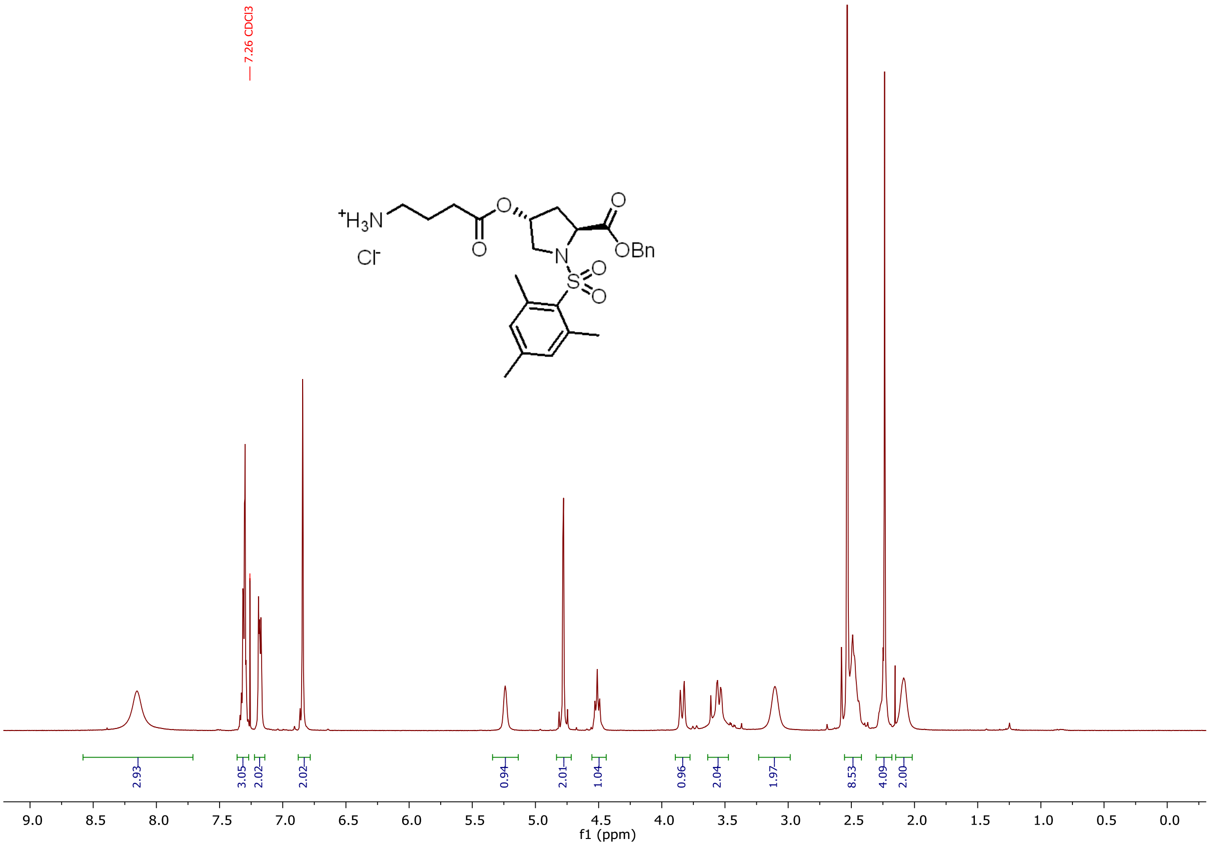

^{13}C NMR (101 MHz, $CDCl_3$) of Compound **22**

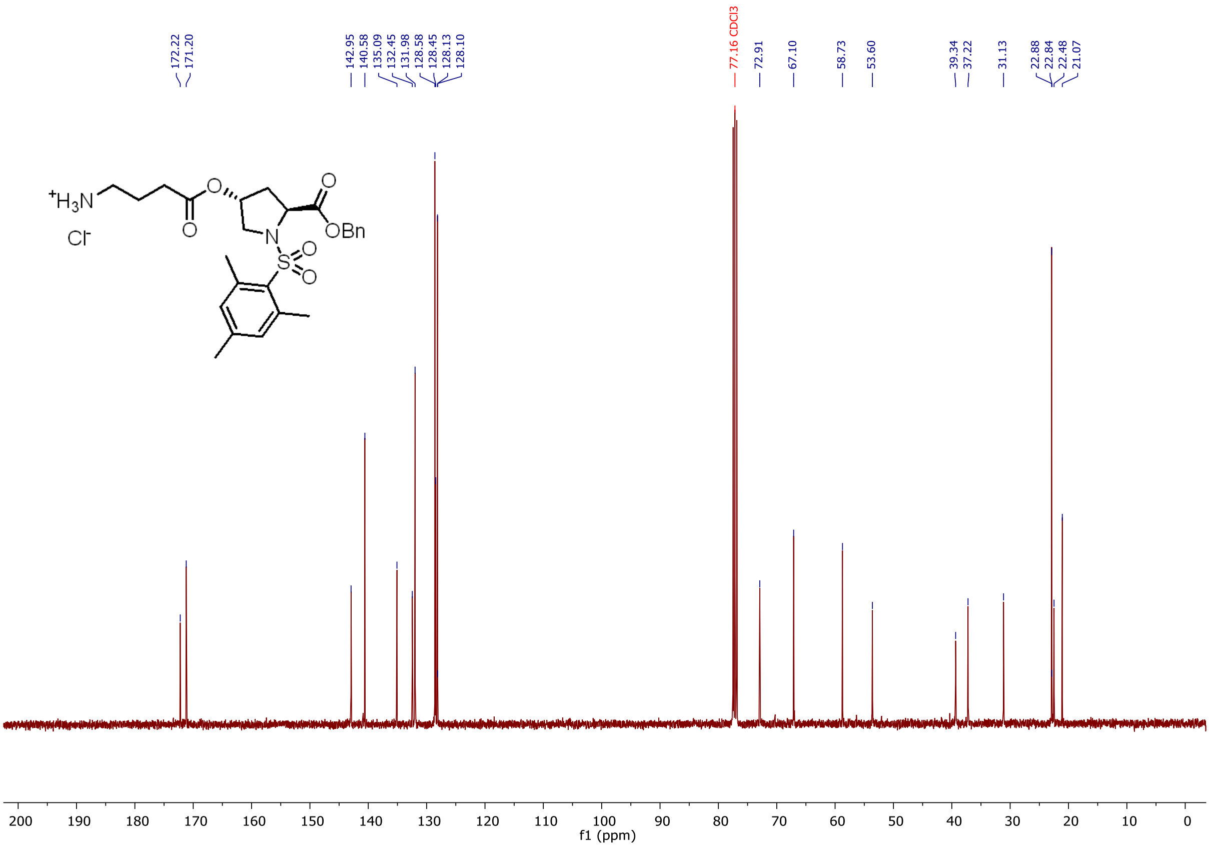

^{1}H NMR (400 MHz, $CDCl_3$) of Compound **23**

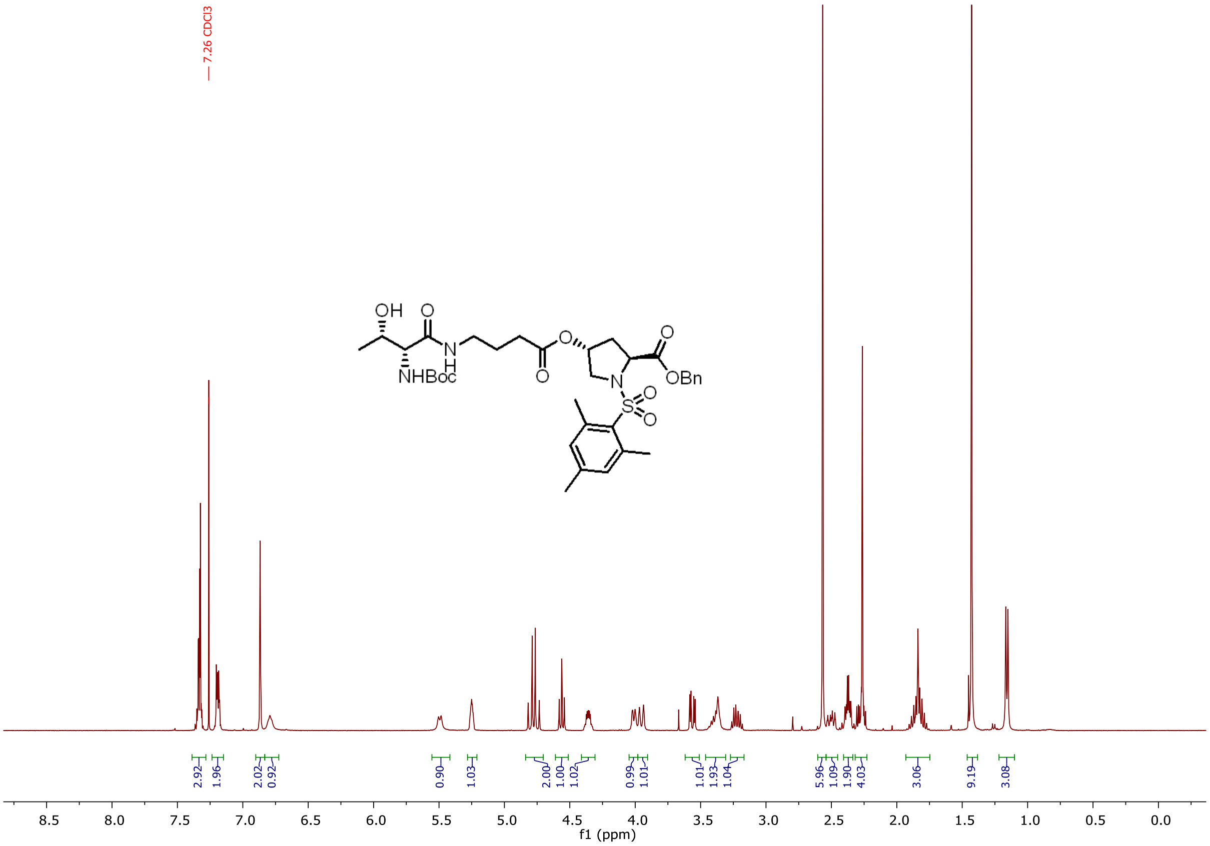

^{13}C NMR (101 MHz, $CDCl_3$) of Compound **23**

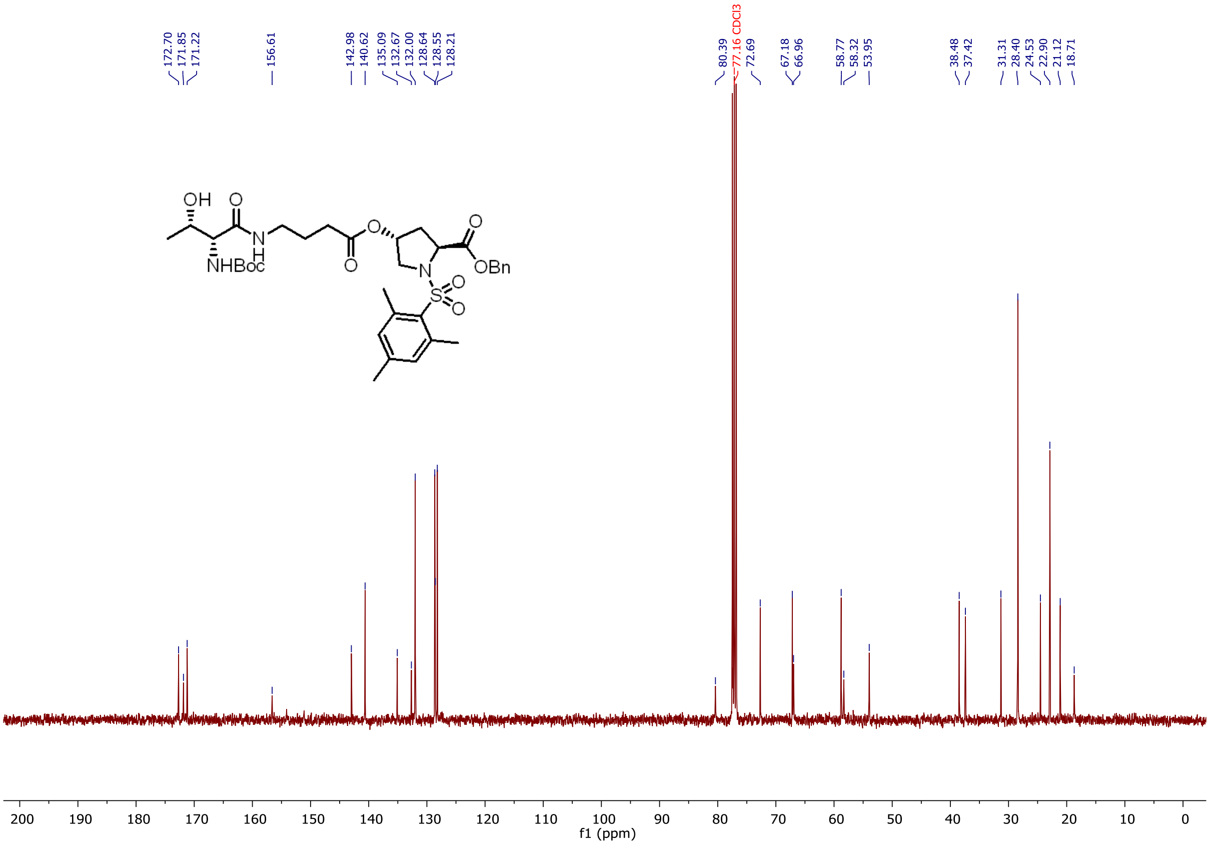

^{1}H NMR (400 MHz, $CDCl_3$) of Compound **24**

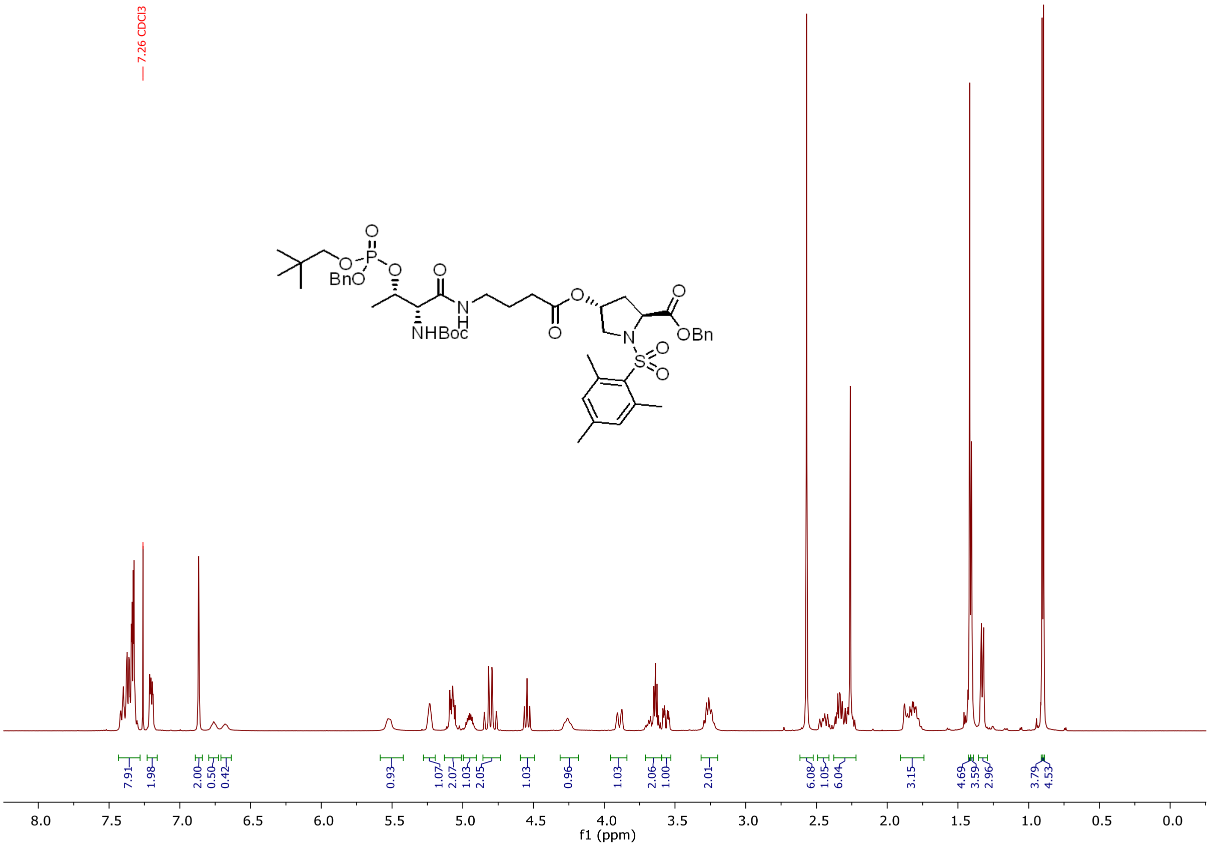

^{13}C NMR (101 MHz, $CDCl_3$) of Compound **24**

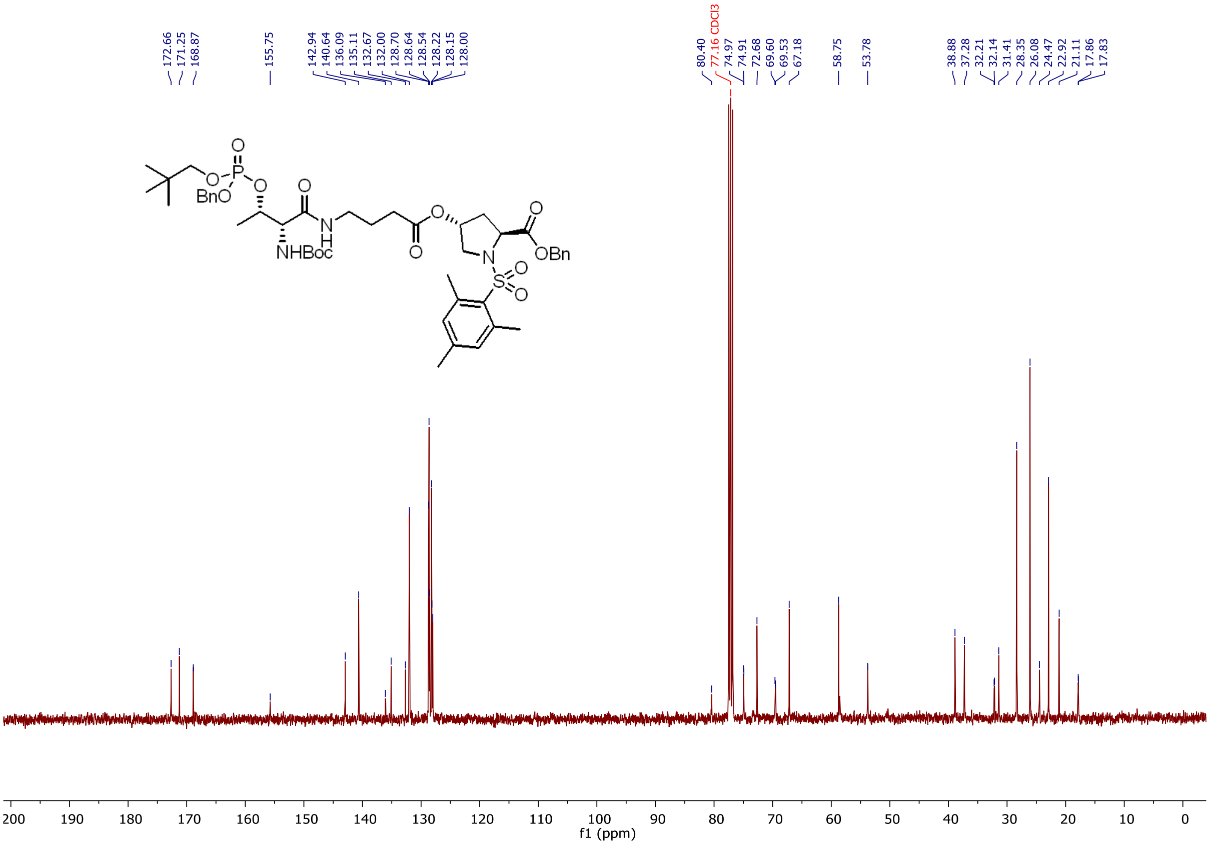

^{1}H NMR (400 MHz, $CDCl_3$) of Compound **25**

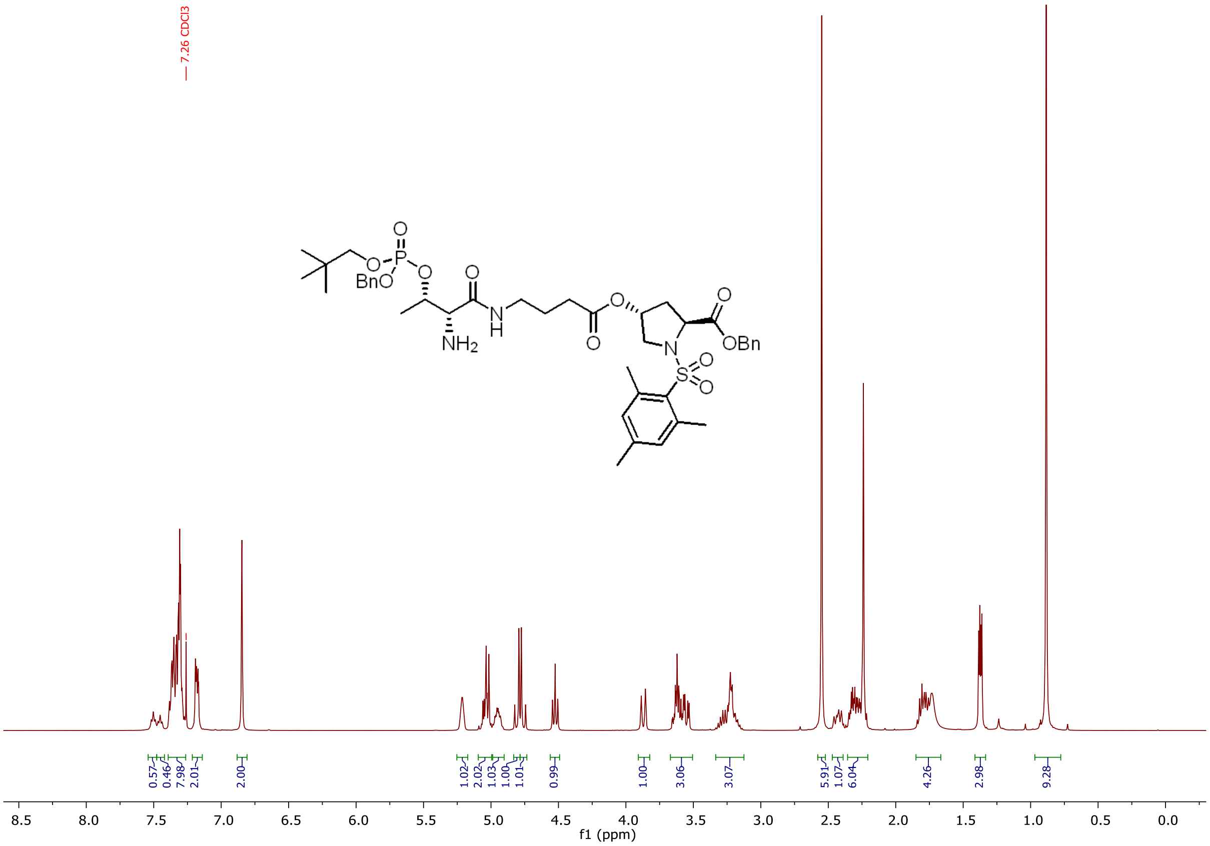

^{13}C NMR (101 MHz, $CDCl_3$) of Compound **25**

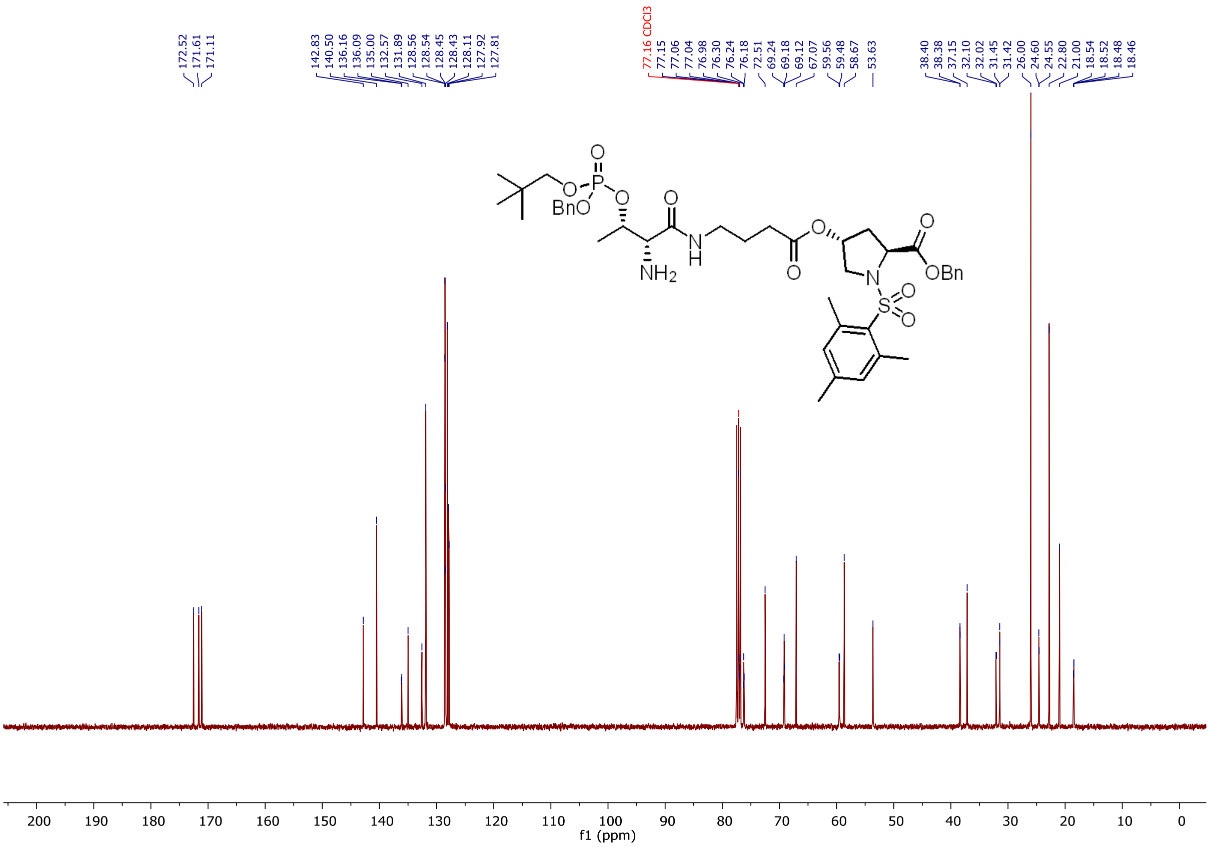

^{1}H NMR (400 MHz, $CDCl_3$) of Compound **26**

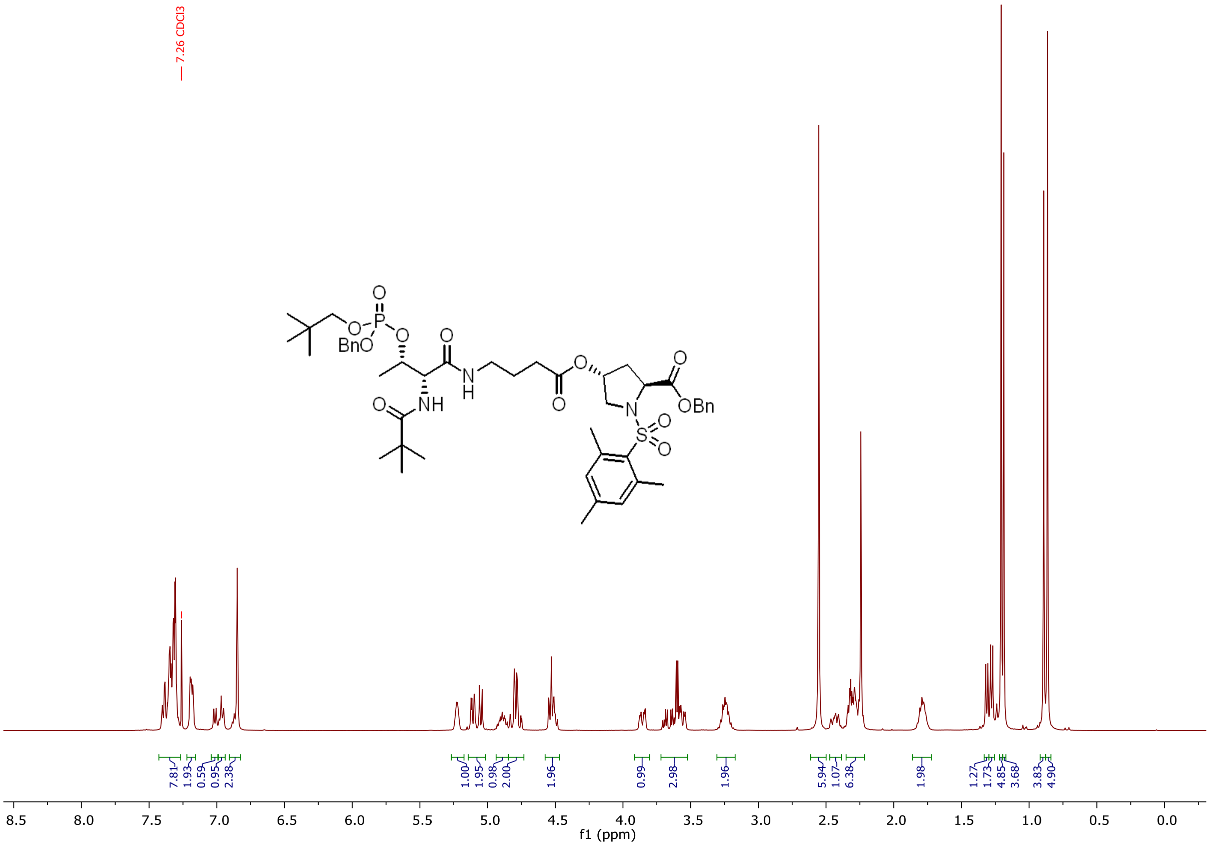

^{13}C NMR (101 MHz, $CDCl_3$) of Compound **26**

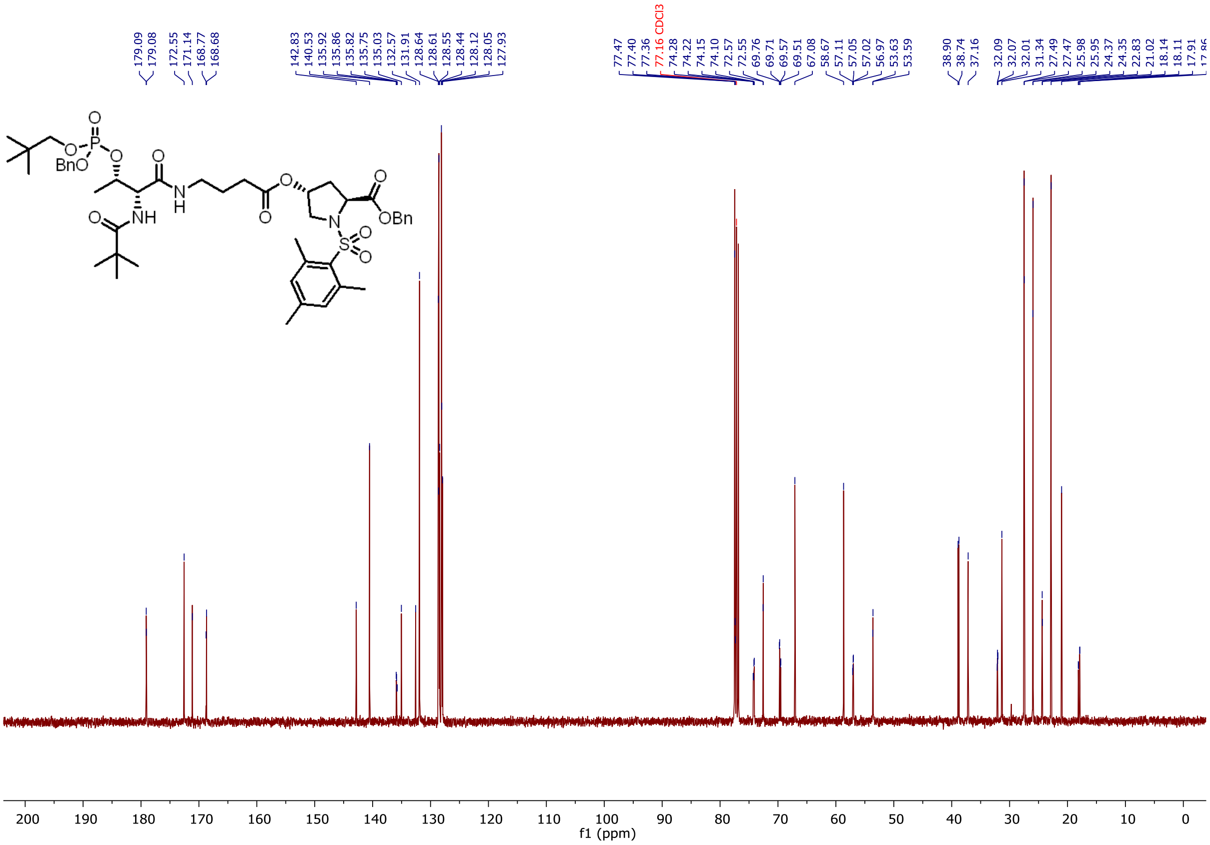

^{1}H NMR (400 MHz, CD_3OD) of Compound **4**

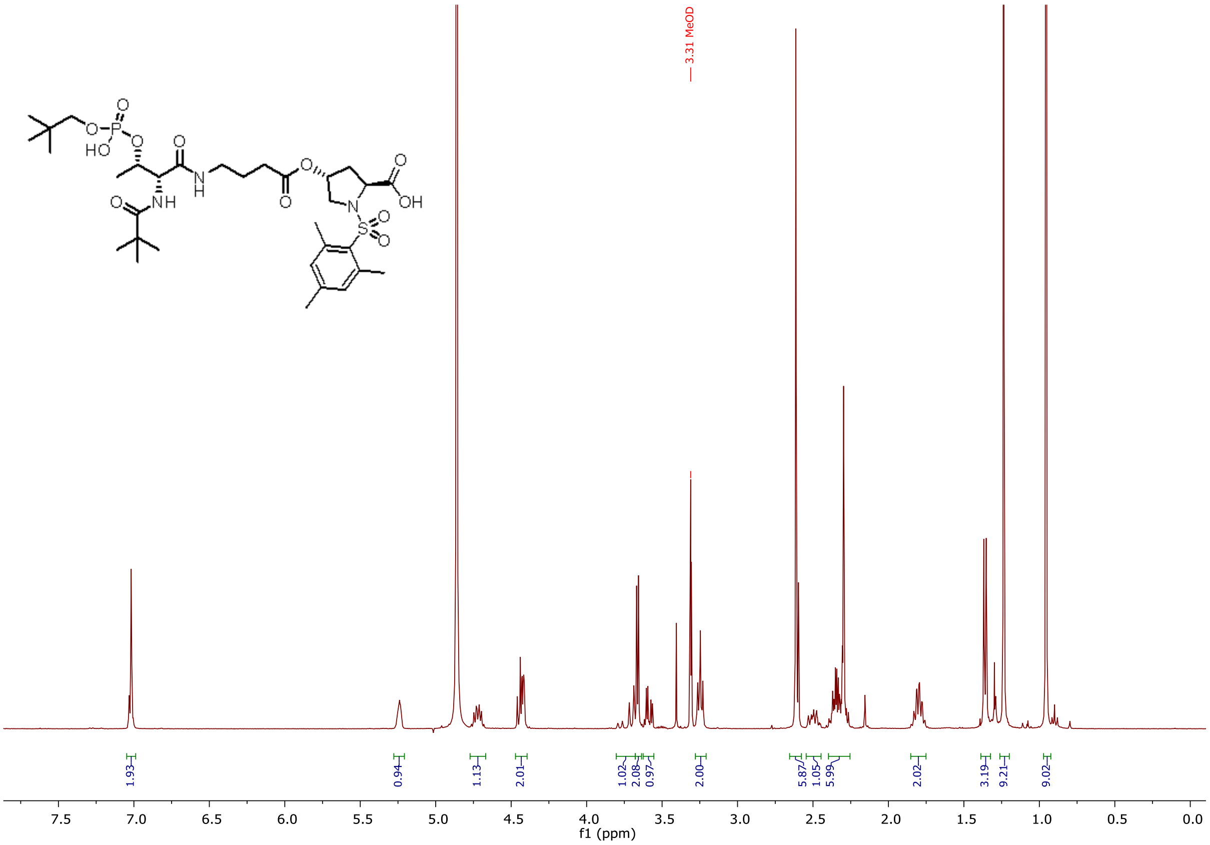

^{13}C NMR (101 MHz, CD_3OD) of Compound **4**

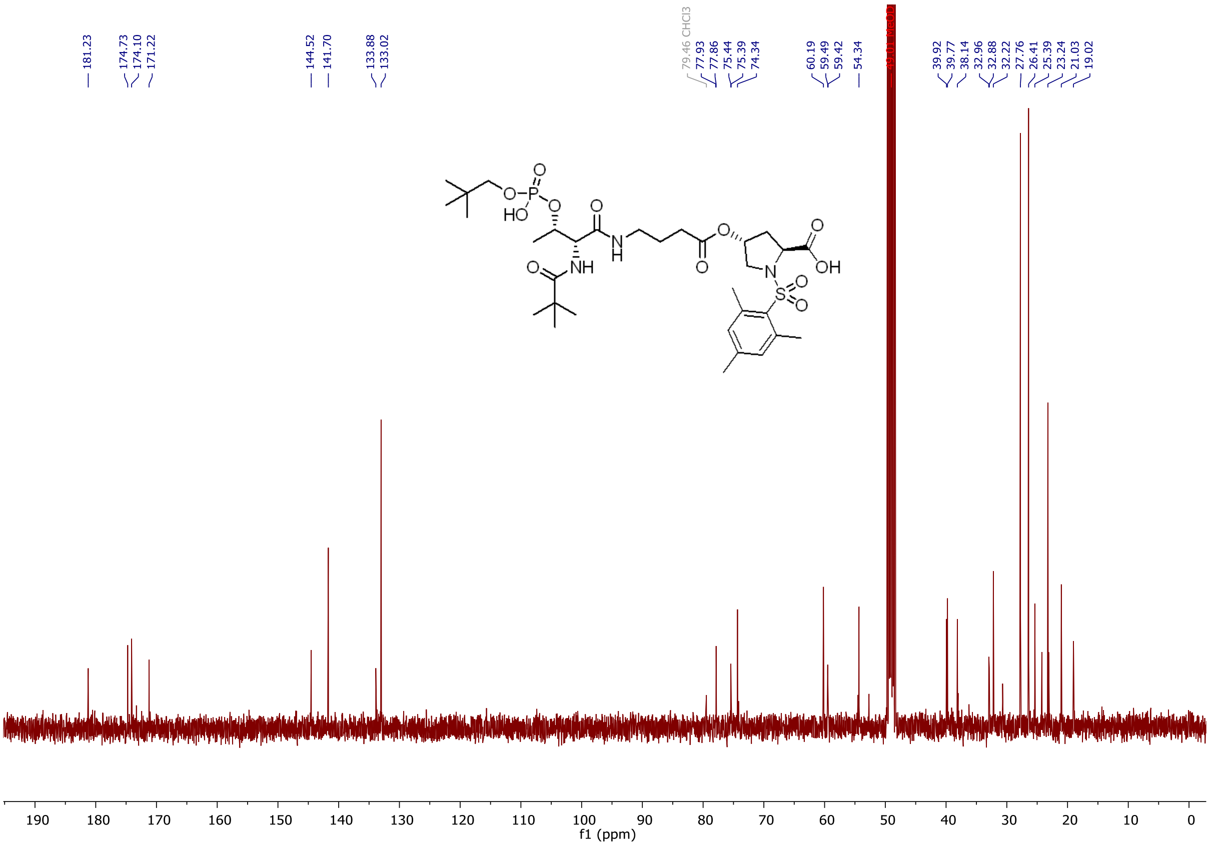

S6 References

[1] M. M. Wootten, B. A. F. Le Bailly, S. Tshepelevitsh, I. Leito, J. Clayden, *Chem. Sci.* **2022**, *13*, 2258–2269.

[2] W. Bannwarth, A. Trzeciak, *Helv. Chim. Acta* **1987**, *70*, 175–186.

[3] T. Ezawa, S. Jung, Y. Kawashima, T. Noguchi, N. Imai, *Tetrahedron Asymmetry* **2017**, *28*, 75–83.

[4] C. M. Dreef-Tromp, A. W. M. Lefeber, G. A. Van Der Marel, J. H. Van Boom, *Synthesis* **1992**, 1269–1272.

[5] D. J. Keith, S. D. Townsend, *J. Am. Chem. Soc.* **2019**, *141*, 12939–12945.

[6] J. Inanaga, K. Hirata, H. Saeki, T. Katsuki, M. Yamaguchi, *Bull. Chem. Soc. Jpn.* **1979**, *52*, 1989–1993.

[7] M. Hatano, K. Moriyama, T. Maki, K. Ishihara, *Angew. Chem. Int. Ed.* **2010**, *49*, 3823–3826.

[8] M. Klussmann, L. Ratjen, S. Hoffmann, V. Wakchaure, R. Goddard, B. List, *Synlett* **2010**, 2189–2192.

[9] M. Rueping, B. J. Nachtsheim, R. M. Koenigs, W. Ieawsuwan, *Chem. Eur. J.* **2010**, *16*, 13116–13126.

[10] R. Jastrzab, *J. Coord. Chem.* **2013**, *66*, 98–113.

[11] S. Tshepelevitsh, A. Kütt, M. Lõkov, I. Kaljurand, J. Saame, A. Heering, P. G. Plieger, R. Vianello, I. Leito, *Eur. J. Org. Chem.* **2019**, 6735–6748.

[12] http://app.supramolecular.org/bindfit, accessed 20/06/22

[13] D. B. Hibbert, P. Thordarson, *Chem. Commun.* **2016**, *52*, 12792–12805.

[14] A. Klamt, *J. Phys. Chem.* **1995**, *99*, 2224–2235.

[15] A. Klamt, V. Jonas, T. Bürger, J. C. W. Lohrenz, *J. Phys. Chem. A* **1998**, *102*, 5074–5085.

[16] F. Eckert, A. Klamt, *AIChE J.* **2002**, *48*, 369–385.

[17] *TURBOMOLE V7.2 2017, a Development of University of Karlsruhe and Forschungszentrum Karlsruhe GmbH, 1989-2007, TURBOMOLE GmbH, since 2007*; Available from http://www.turbomole.com

[18] *BIOVIA COSMOtherm, Release 2021; Dassault Systèmes*. http://www.3ds.com

[19] A. Kütt, S. Tshepelevitsh, J. Saame, M. Lõkov, I. Kaljurand, S. Selberg, I. Leito, *Eur. J. Org. Chem.* **2021**, *2021*, 1407–1419.

[20] M. K. Chantooni, I. M. Kolthoff, *Anal. Chem.* **1979**, *51*, 133–140.

[21] I. M. Kolthoff, M. K. Chantooni, *J. Am. Chem. Soc.* **1975**, *97*, 1376–1381.

[22] I. M. Kolthoff, M. K. Chantooni, *J. Chem. Eng. Data* **1999**, *44*, 124–129.

[23] A. Kütt, T. Rodima, J. Saame, E. Raamat, V. Mäemets, I. Kaljurand, I. A. Koppel, R. Y. Garlyauskayte, Y. L. Yagupolskii, L. M. Yagupolskii, E. Bernhardt, H. Willner, I. Leito, *J. Org. Chem.* **2011**, *76*, 391–395.

[24] S. Espinosa, E. Bosch, M. Rosés, *J. Chromatogr. A* **2002**, *964*, 55–66.
